# Ubiquitin-dependent proteolysis of KNL2 driven by APC/C^CDC20^ is critical for centromere integrity and mitotic fidelity

**DOI:** 10.1093/plcell/koaf164

**Published:** 2025-06-25

**Authors:** Manikandan Kalidass, Venkata Ganesh Jarubula, Maryia Ratnikava, Jothipriya Ramakrishnan Chandra, Samuel Le Goff, Aline V Probst, Silvia Esposito, Klaus D Grasser, Astrid Bruckmann, Jérôme F Gagneux, Reinier F Prosée, Twan Rutten, Veit Schubert, Dmitri Demidov, Esther Lechner, Florian A Steiner, Pascal Genschik, Inna Lermontova

**Affiliations:** Kinetochore Biology, Department of Breeding Research, Leibniz Institute of Plant Genetics and Crop Plant Research (IPK) OT Gatersleben, Corrensstr 3, Seeland 06466, Germany; Kinetochore Biology, Department of Breeding Research, Leibniz Institute of Plant Genetics and Crop Plant Research (IPK) OT Gatersleben, Corrensstr 3, Seeland 06466, Germany; Kinetochore Biology, Department of Breeding Research, Leibniz Institute of Plant Genetics and Crop Plant Research (IPK) OT Gatersleben, Corrensstr 3, Seeland 06466, Germany; Kinetochore Biology, Department of Breeding Research, Leibniz Institute of Plant Genetics and Crop Plant Research (IPK) OT Gatersleben, Corrensstr 3, Seeland 06466, Germany; iGReD, Université Clermont Auvergne, CNRS, INSERM, BP 38, Clermont-Ferrand 63001, France; iGReD, Université Clermont Auvergne, CNRS, INSERM, BP 38, Clermont-Ferrand 63001, France; Cell Biology and Plant Biochemistry, Biochemistry Center, University of Regensburg, Universitätsstrasse 31, Regensburg D-93053, Germany; Cell Biology and Plant Biochemistry, Biochemistry Center, University of Regensburg, Universitätsstrasse 31, Regensburg D-93053, Germany; Institute for Biochemistry I, Center for Biochemistry, University of Regensburg, Universitätsstrasse 31, Regensburg D-93053, Germany; Department of Molecular and Cellular Biology, Institute for Genetics and Genomics in Geneva, Section of Biology, Faculty of Sciences, University of Geneva, Geneva 1211, Switzerland; Department of Molecular and Cellular Biology, Institute for Genetics and Genomics in Geneva, Section of Biology, Faculty of Sciences, University of Geneva, Geneva 1211, Switzerland; Kinetochore Biology, Department of Breeding Research, Leibniz Institute of Plant Genetics and Crop Plant Research (IPK) OT Gatersleben, Corrensstr 3, Seeland 06466, Germany; Kinetochore Biology, Department of Breeding Research, Leibniz Institute of Plant Genetics and Crop Plant Research (IPK) OT Gatersleben, Corrensstr 3, Seeland 06466, Germany; Kinetochore Biology, Department of Breeding Research, Leibniz Institute of Plant Genetics and Crop Plant Research (IPK) OT Gatersleben, Corrensstr 3, Seeland 06466, Germany; Institut de Biologie Moléculaire des Plantes, CNRS, Université de Strasbourg, 12 rue Général Zimmer, Strasbourg Cedex 67084, France; Department of Molecular and Cellular Biology, Institute for Genetics and Genomics in Geneva, Section of Biology, Faculty of Sciences, University of Geneva, Geneva 1211, Switzerland; Institut de Biologie Moléculaire des Plantes, CNRS, Université de Strasbourg, 12 rue Général Zimmer, Strasbourg Cedex 67084, France; Kinetochore Biology, Department of Breeding Research, Leibniz Institute of Plant Genetics and Crop Plant Research (IPK) OT Gatersleben, Corrensstr 3, Seeland 06466, Germany

## Abstract

Kinetochores are large protein complexes that serve as attachment sites for spindle microtubules, ensuring proper chromosome segregation during cell division. KINETOCHORE NULL2 (αKNL2) is a key kinetochore protein required for the incorporation of the centromeric histone variant CENH3. The precise regulation of αKNL2 levels is crucial, but the molecular mechanisms controlling this process remain largely unexplored. In this study, we demonstrated that the Anaphase-Promoting Complex/Cyclosome (APC/C) mediates the ubiquitin-dependent proteolysis of αKNL2 during mitosis. Our findings revealed that αKNL2 accumulates in the presence of 26S proteasome inhibitors, and our yeast 2-hybrid and proteomic screens showed that proteins from the ubiquitin-proteasome pathway interact with KNL2 in Arabidopsis (*Arabidopsis thaliana*) and nematode (*Caenorhabditis elegans*). Arabidopsis αKNL2 directly interacts with Anaphase-Promoting Complex subunit 10 (APC10) and Cell Division Cycle 20.1 (CDC20.1), 2 substrate recognition components of the APC/C. RNAi-mediated depletion of APC/C resulted in the accumulation and mislocalization of endogenous αKNL2. Additionally, mutation or deletion of the D-box1 region, or substitution of residues K336 and K339, impaired αKNL2 degradation. The expression of a proteasome-resistant αKNL2 variant in planta caused severe defects in growth, fertility, and mitotic division. These findings show that APC/C^CDC20^-mediated degradation of αKNL2 is critical for proper kinetochore function and centromere integrity.

## Teaser

Controlled degradation of the kinetochore protein KNL2 during cell division preserves genome stability and promotes normal plant growth and development.

## Introduction

Errors in chromosome segregation lead to the generation of cells with an aberrant number of chromosomes, disrupting normal cell division. The centromere is a specialized region of the chromosome where the kinetochore protein complex assembles, facilitating the separation of sister chromatids via the attachment of microtubules during cell division. In most organisms, kinetochore establishment depends on the centromeric histone variant, CENH3 (Ishii and Akiyoshi 2022; Naish and Henderson 2024), initially described as CENP-A in humans (Earnshaw and Rothfield 1985), which marks active centromeres. Genome replication during the S phase results in the dilution of CENH3; therefore, the incorporation of new CENH3 is required. The deposition of CENH3 at centromeres occurs during anaphase/telophase to mid-G1 in animals (Jansen et al. 2007; Mellone et al. 2011) and mainly during G2 in plants (Lermontova et al. 2007). Although the mechanisms of centromeric chromatin maintenance differ among species, they generally rely on the timely deposition of CENH3, facilitated by licensing factors and histone chaperones (Fujita et al. 2007; Dunleavy et al. 2009; Lermontova et al. 2013; Le Goff et al. 2020).

KINETOCHORE NULL2 (KNL2/MIS18BP1) acts as a licensing factor for CENH3 loading and is essential for centromere establishment (Lermontova et al. 2013; Sandmann et al. 2017). In humans and nematode (*C. elegans*), KNL2 features a C-terminal SANT/MYB domain and an N-terminal SANT-associated (SANTA) domain (Maddox et al. 2007). In contrast, Arabidopsis (*Arabidopsis thaliana*) KNL2, referred to as αKNL2, contains only the N-terminal SANTA domain (Lermontova et al. 2013). However, the C-terminus of αKNL2 in plants and most vertebrates contains a CENPC-like motif responsible for centromere targeting (Kral 2015; French et al. 2017; Hori et al. 2017; Sandmann et al. 2017). Additionally, in plants, the kinetochore protein βKNL2 contains only a SANTA domain localized to centromeres despite lacking a CENPC-like motif (Zuo et al. 2022; Yadala et al. 2024). In *C. elegans*, KNL-2 (*Ce*KNL-2) and CENP-A interact directly, with their chromatin binding being mutually dependent and exhibiting a similar localization pattern spanning the entire chromosomal length (Maddox et al. 2007; de Groot et al. 2021; Prosée et al. 2021). Loss of the KNL2 function leads to defects in chromosome segregation, condensation, and centromere maintenance in Arabidopsis and nematode (Maddox et al. 2007; Lermontova et al. 2013). Overexpression of centromere or kinetochore proteins disrupts the precise spatiotemporal regulation of CENH3 deposition, leading to mislocalization. This process is tightly controlled by ubiquitin-mediated proteolysis (Moreno-Moreno et al. 2011; Shrestha et al. 2017; Wang et al. 2021). We proposed previously that the ubiquitin-proteasome system (UPS) regulates αKNL2 stability in plants, as fluorescence signals in *A. thaliana* transformants expressing full-length αKNL2-EYFP under the 35S promoter were detected after proteasome inhibitor treatment. Furthermore, the absence of αKNL2 from centromeres during metaphase to anaphase suggests a stringent degradation control during mitosis, ensuring proper cell cycle progression (Lermontova et al. 2013).

Despite these findings, the molecular mechanisms governing αKNL2 degradation are still not well understood. Therefore, it is relevant to explore whether similar mechanisms regulate MIS18BP1/KNL2 in other organisms. However, the role of ubiquitination in MIS18BP1/KNL2 regulation in vertebrates and nematode remains unclear. Betts (2010) demonstrated that in humans, MIS18BP1/KNL2 is targeted for degradation by APC/C via its D-box and KEN-box motifs. Mutations in these motifs stabilized KNL2, although its degradation was not essential for CENP-A loading at centromeres. Beyond this, no other studies have reported the proteasome-mediated degradation of MIS18BP1/KNL2, highlighting a significant gap in our understanding of its regulatory mechanisms.

Ubiquitination, the process of attaching the polypeptide ubiquitin to a substrate, is a crucial posttranslational modification in many eukaryotic cells (Hershko and Ciechanover 1998; Ciechanover et al. 2000). The ubiquitin-proteasome pathway involves 3 key steps: activation, conjugation, and ligation, each requiring a specific type of enzyme such as ubiquitin-activating enzymes (E1), ubiquitin-conjugating enzymes (E2), and ubiquitin ligases (E3). Initially, ubiquitin is activated through its attachment to E1 enzymes, after which it is transferred to E2 enzymes. E3 ligases then interact with the E2-ubiquitin complex, facilitating the transfer of ubiquitin to the target protein. Proteins tagged with ubiquitin are eventually recognized and degraded by the 26S proteasome system (Hershko and Ciechanover 1998). Ubiquitination of a substrate protein can be divided into (i) monoubiquitination, which can also occur at multiple Lys residues (called multimonoubiquitination), and (ii) polyubiquitination which takes place when ubiquitin monomers are conjugated by another ubiquitin through any of its 7 Lys residues giving raise to different ubiquitin chain topologies with different outcomes (Kwon and Ciechanover 2017).

During the cell cycle, targeted degradation is critical for regulating steady-state levels of key regulatory proteins and also preventing their mislocalization (Mocciaro and Rape 2012). E3 ubiquitin ligases such as the Anaphase-Promoting Complex or Cyclosome (APC/C) and Cullin-RING ligases (CRLs) regulate protein degradation and cell cycle progression. The APC/C is a large complex composed of 11 to 13 subunits that function during mitosis to facilitate sister chromatid separation and mitotic exit by degrading mitotic substrates (Pesin and Orr-Weaver 2008; Genschik et al. 2014; Willems and De Veylder 2022). Moreover, the APC/C targets the substrate for degradation by binding to at least 1 of the 3 degron motifs: the ABBA motif, the KEN box, or the D-box region. Cell Division Cycle 20 (CDC20), also known as Fizzy (FZY), and Cell Division Cycle 20 Homolog (CDH1), also called Fizzy-related (FZR), serve as substrate-specific activators of the APC/C (Visintin et al. 1997; Yeong 2004). CDC20 primarily functions from prometaphase to anaphase, whereas CDH1 mainly facilitates the exit from mitosis and operates during G1 and early S phases, contributing to the degradation of specific proteins essential for cell cycle regulation (Willems and De Veylder 2022). The interdependent action of these proteins with the APC/C enables precise control over the degradation of critical cell cycle regulators.

KNL2 is required for the CENH3 loading and is tightly regulated at the protein level. This study elucidates the regulation of αKNL2 levels via the APC/C^CDC20^-dependent degradation pathway. We demonstrate that αKNL2 is an unstable protein that undergoes polyubiquitination, leading to its degradation by the 26S proteasome. In *A. thaliana* and *C. elegans*, yeast 2-hybrid (Y2H) and proteomic screenings revealed that KNL2 interacts with proteins involved in the ubiquitin-proteasome pathway, including direct interactions with APC/C coactivators. We specifically showed that αKNL2 accumulates during the prometaphase to anaphase stages in RNAi *APC10* mutants. Further, the mutational analysis identified the D-box1 region of αKNL2 as a critical degron for APC/C^CDC20^-mediated degradation and lysine residues K336 and K339 as essential sites for polyubiquitination of αKNL2. Overaccumulation of degradation-resistant αKNL2 results in severe growth, fertility, and chromosome segregation defects, highlighting the necessity of stringent posttranslational regulation of αKNL2 for proper mitosis.

## Results

### The ubiquitin-proteasome pathway mediates the degradation of polyubiquitinated αKNL2

**Figure 1.**
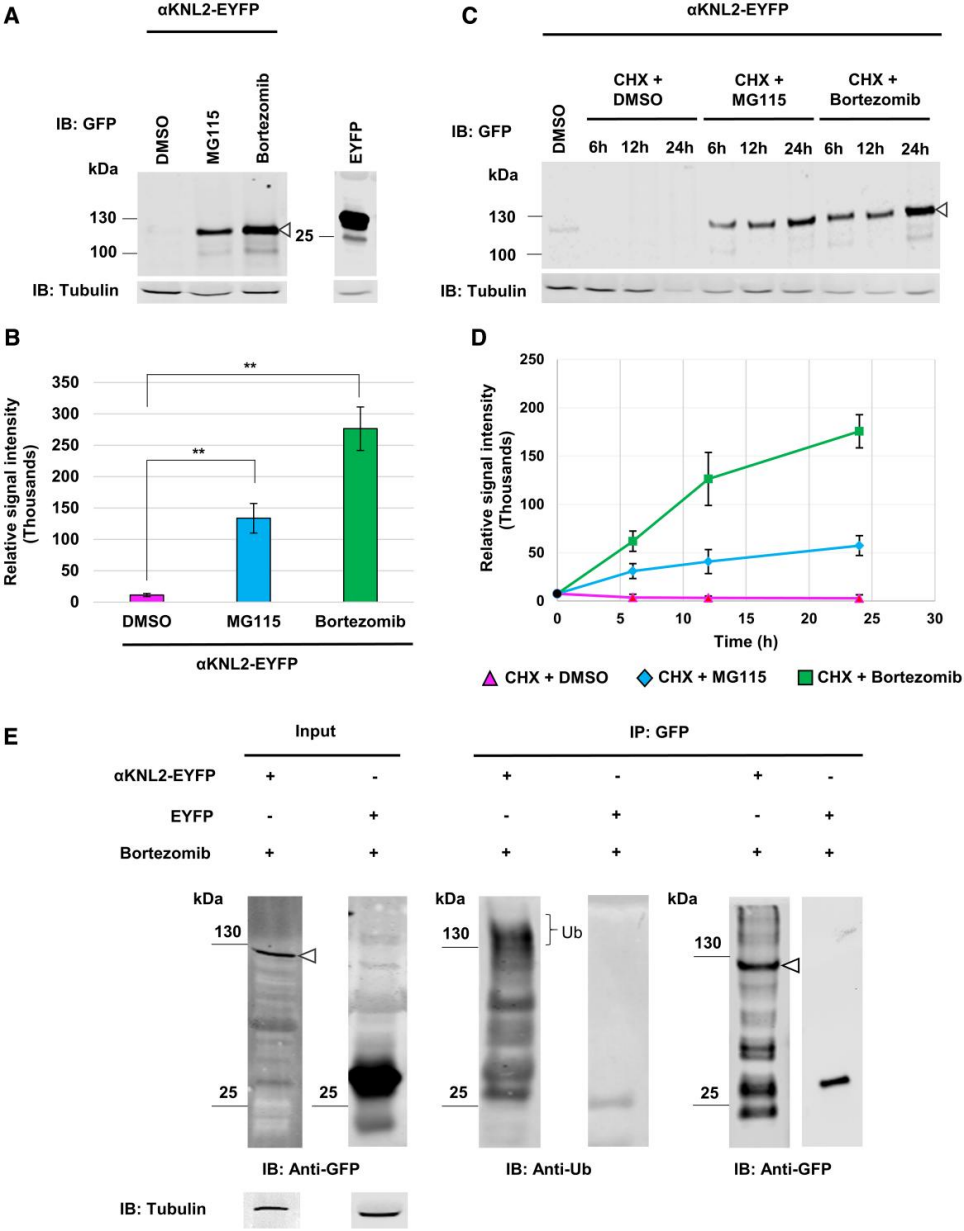
Polyubiquitination and degradation of αKNL2 in vivo. **A and B)** Analysis of αKNL2 protein levels in *N. benthamiana* leaves transiently expressing αKNL2-EYFP in the presence of 26S proteasome inhibitors. **A)** Western blot analysis of total protein extracts from *N. benthamiana* leaves expressing αKNL2-EYFP, treated with DMSO (control), MG115, or bortezomib (100 *µ*M each) 2 d postinfiltration. Anti-GFP antibodies were used for detection; leaves infiltrated with an EYFP-expressing vector served as a negative control. Tubulin was used as a loading control. The triangle symbol (▵) indicates the MW of αKNL2. **B)** Quantification of αKNL2 protein levels in different treatment groups. The ratio of αKNL2 versus tubulin expression was used for the measurement of the αKNL2 levels. Data are presented as means ± SEM (*n* = 3). Significant differences between groups were assessed using Welch's *t*-test and are indicated by ** (*P* < 0.05). **C and D)** Examination of αKNL2 protein levels in *N. benthamiana* leaves expressing αKNL2-EYFP in the presence of 26S proteasome and translational inhibitors. **C)** Western blot analysis of total protein extracts from leaves treated with DMSO (control) or CHX combined with either DMSO, MG115, or bortezomib (100 *µ*M each) at 2 d postinfiltration. Detection was performed using anti-GFP antibodies; leaves infiltrated with an EYFP-expressing vector were used as a negative control. Tubulin served as a loading control. The triangle symbol (▵) indicates the MW of αKNL2. **D)** Quantification of αKNL2 protein levels over time across different treatment groups. The ratio of αKNL2 versus tubulin expression was used for the measurement of the αKNL2 levels. Data are presented as means ± SEM (*n* = 3). **E)** Ubiquitination assay of αKNL2 in *N. benthamiana* leaves expressing αKNL2-EYFP treated with bortezomib 2 d postinfiltration. Leaves expressing EYFP alone served as a control. Total protein extracts were subjected to IP using GFP magnetic agarose beads. Input samples were immunoblotted with anti-GFP (left panel), and immunoprecipitated samples were probed with anti-ubiquitin (Ub) (center panel) or anti-GFP (right panel). Tubulin served as a loading control. The triangle symbol (▵) indicates the MW of αKNL2, and the bracket marks the ubiquitinated form of αKNL2. IB, immunoblot; IP, immunoprecipitation.

In our previous study, we speculated that the αKNL2 protein levels in Arabidopsis could be regulated by the proteasomal system (Lermontova et al. 2013). To explore how αKNL2 undergoes degradation via the 26S proteasome, *Nicotiana benthamiana* plants were infiltrated with *Agrobacterium* (*Agrobacterium tumefaciens*) expressing a plasmid encoding enhanced yellow fluorescent protein (EYFP) fused to the full-length αKNL2 under the control of a 35S promoter (αKNL2-EYFP). At 2 d postinfiltration, leaves expressing αKNL2-EYFP were infiltrated with 26S proteasome inhibitors MG115/bortezomib, or DMSO as a control. After 18 h, total protein extracts were isolated and the αKNL2 levels were assessed by western blot using an anti-GFP monoclonal antibody. The immunoblot (IB) analysis revealed a significant increase in αKNL2 protein levels in the presence of MG115 or bortezomib while αKNL2 protein was barely detected in leaves infiltrated with only DMSO (Fig. 1, A and B). Thus, these results reveal that αKNL2 stability was strongly increased in the presence of 26S proteasome inhibitors.

To further demonstrate the 26S proteasome-mediated degradation of αKNL2, a cycloheximide (CHX) chase assay was performed. CHX partially inhibits the translation machinery, reducing the level of intracellular proteins subjected to proteasome degradation. *N. benthamiana* leaves transiently expressing αKNL2-EYFP were treated with DMSO (control) or CHX in combination with either DMSO, MG115, or bortezomib, and samples were harvested at different time points (6, 12, and 24 h). αKNL2 protein was quickly degraded in samples treated with CHX, revealing a half-life of αKNL2 of less than 6 h. In contrast, αKNL2 protein decay was blocked in the MG115/bortezomib-treated samples compared to DMSO-treated leaves (Fig. 1, C and D), indicating that αKNL2 is an unstable protein that undergoes degradation via the proteasomal pathway.

To determine if αKNL2 is ubiquitinated in planta, total proteins were extracted from leaves of *N. benthamiana* infiltrated with the αKNL2-EYFP treated with bortezomib. Extracts from samples expressing EYFP alone were used as a control. The proteins were immunoprecipitated using GFP beads and then immunoblotted with either a mouse anti-GFP monoclonal antibody or an anti-ubiquitin antibody. The results revealed bands corresponding to the molecular weight (MW) of the αKNL2-EYFP fusion protein and a high MW smear signal in the αKNL2-EYFP sample treated with bortezomib, but not in the EYFP control (Fig. 1E). Thus, this demonstrates that αKNL2 is polyubiquitinated and degraded by the ubiquitin-proteasome pathway.

### Proteomic and Y2H screening identify components of the ubiquitin-proteasome pathway as interactors of KNL2

**Figure 2.**
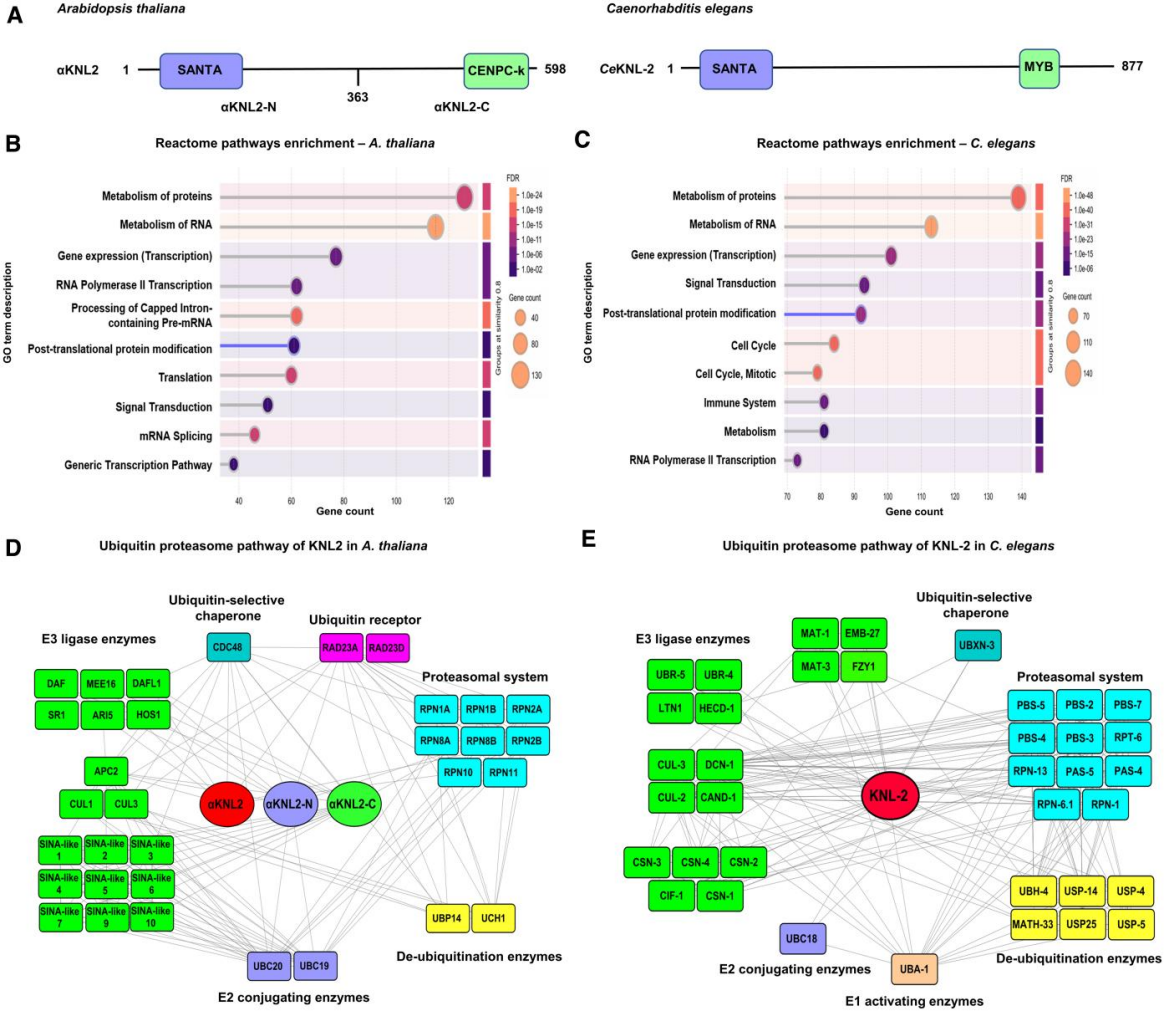
Identification of KNL2 interaction partners reveals a link between KNL2 and ubiquitin-proteasome pathway in *A. thaliana* and *C. elegans.*  **A)** The domain organization of KNL2 in *A. thaliana* and *C. elegans.* In Arabidopsis, the full-length KNL2 protein (αKNL2; 1 to 598 amino acids), as well as its N-terminal (αKNL2-N; 1 to 363 amino acids) and C-terminal (αKNL2-C; 364 to 599 amino acids) fragments, was shown. The KNL2 contains 2 functional domains: the SANTA and CENPC-k motifs. In *C. elegans*, the full-length KNL2 protein (*Ce*KNL-2; 1 to 877 amino acids) was shown, featuring both SANTA and MYB domains. **B and C)** The top 10 GO enrichment terms (Reactome pathways) of the KNL2 interactors in *A. thaliana*  **B)** and *C. elegans*  **C)**. The graph displays the number of genes associated with each GO term (gene count) along with the corresponding enriched GO terms. Dot size indicates gene count, while color represents the false discovery rate, with lighter colors denoting higher significance. The GO term posttranslational protein modification was highlighted (violet). **D and E)** Interaction network illustrating the association of KNL2 with an ubiquitin-proteasome pathway in *A. thaliana*  **D)** and *C. elegans*  **E)**. Proteins identified as interactors of KNL2 are categorized based on functional annotations. The nodes represent KNL2 interactors, and the edges between the nodes indicate interactions between the proteins. The node colors indicate the different functional groups of the ubiquitin-proteasome pathway. The network was constructed using STRING and Cytoscape programs.

KNL2 is known to function as a licensing factor for CENH3 loading and kinetochore assembly in various eukaryotes (Fujita et al. 2007; Maddox et al. 2007; Lermontova et al. 2013). Nevertheless, it is still unclear whether KNL2 possesses different functions or undergoes protein-level modifications that influence centromeric homeostasis. To gain deeper insights into KNL2 function and explore its degradation mechanisms, we aimed to identify KNL2 interactors in *A. thaliana* and *C. elegans*, providing a broader perspective on KNL2 regulation in plants and animals. To achieve this, the full-length Arabidopsis αKNL2 protein (αKNL2, 1 to 598 aa), along with its N-terminal (αKNL2-N; 1 to 363 aa) and C-terminal (αKNL2-C; 364 to 599 aa) fragments (Fig. 2A), were employed for Y2H library screening. Additionally, the N- and C-terminal fragments fused to the GS tag were transformed into Arabidopsis cell culture and used for affinity purification combined with mass spectrometry (AP-MS). In *C. elegans*, the full-length, HA-tagged KNL-2 protein (*Ce*KNL-2, 1 to 877 aa) was used for immunoprecipitation-mass spectrometry (IP-MS) (Fig. 2A).

αKNL2, αKNL2-N, and αKNL2-C fused to the *GAL4* DNA-binding domain (BD) were used as baits for screening against an Arabidopsis cDNA library from 11 plant tissues fused to the *GAL4* activation domain (AD). While performing the Y2H library screening, we selected only the clones where a gene encoding a αKNL2 interactor was in-frame with the *GAL4* DNA AD. This yielded 51, 85, and 173 positive colonies for the αKNL2, αKNL2-N, and αKNL2-C, respectively. Considering redundant positive clones, 38 potential direct interactors for αKNL2, 54 for αKNL2-N, and 76 for αKNL2-C were identified (Supplementary Fig. S1A). As Y2H screening mainly identifies direct interactors and could not detect proteins in a larger complex with αKNL2 in planta, an AP-MS experiment was conducted using Arabidopsis culture cells. Because full-length αKNL2 was difficult to express and purify in Arabidopsis culture cells due to its instability, expression vectors carrying either αKNL2-N or αKNL2-C fused to a GS tag were generated. Three independent affinity purifications were performed for each construct to identify potential αKNL2 interactors. A total of 399 proteins were precipitated with the N-terminal part of αKNL2 in all 3 purifications, while in the case of the C-terminal part of αKNL2, 97 proteins were identified in at least 2 purifications (Supplementary Fig. S1B). Venn-plot analysis showed that 50 proteins were precipitated with both N- and C-terminal parts of αKNL2 (Supplementary Fig. S1B), suggesting that they may either establish multiple interactions with αKNL2 or be components of a larger complex. Moreover, 6 interactors were detected in both AP-MS and Y2H assays, including RAD23A and RAD23D belonging to the ubiquitination pathway (Supplementary Fig. S1, C and D). To investigate and compare the functions of KNL2 across eukaryotes, IP-MS was conducted on worm embryonic lysates using a strain expressing HA-tagged *Ce*KNL-2 from its endogenous locus. Following the HA tag pulldown, 530 proteins were coprecipitated with *Ce*KNL-2 (Supplementary Data Set 1). Using an ortholog identifier, the proteins identified as *Ce*KNL-2 interactors were analyzed for orthologs in Arabidopsis. Notably, 274 proteins (54.09%) were found to have orthologs in Arabidopsis. Among these, 31 are associated with the ubiquitin-proteasome pathway, including anaphase-promoting complex components such as APC8 (*Ce*MAT-3), and CDC20.1 (*Ce*FZY-1) (Supplementary Data Set 1).

To better understand the role of the interacting proteins in linking the known function of KNL2, a Gene Ontology (GO) enrichment analysis was performed. Interestingly, Reactome pathway enrichment analysis revealed that both αKNL2 and *Ce*KNL-2 are mostly involved in similar processes, including transcription, posttranslational modification, metabolism, and the cell cycle (Fig. 2, B and C). Further, GO analysis of Arabidopsis αKNL2 interactors identified through Y2H library screening revealed their involvement in processes such as ubiquitin-dependent pathways, posttranslational modifications, and mechanisms of epigenetic regulation (Supplementary Fig. S2A). Similarly, GO analysis of proteins precipitated with αKNL2 and *Ce*KNL-2 highlighted their roles in functional categories such as cellular transport, cell division, spindle microtubule attachment to the kinetochore, chromatin organization, and gene silencing pathways (Supplementary Fig. S2, B and C). Additionally, network enrichment analysis revealed that αKNL2 and *Ce*KNL-2 interactors are involved in pathways including ubiquitination, spliceosome activity, SUMOylation, transcription, DNA replication, RNA metabolism, and signaling (Supplementary Figs. S3 and S4). A subnetwork was generated showing 32 αKNL2 interactors in Arabidopsis and 37 *Ce*KNL-2 interactors associated with the ubiquitin-proteasome pathway (Fig. 2, D and E), supporting the hypothesis that KNL2 undergoes ubiquitin-dependent proteasomal degradation in both *A. thaliana* and *C. elegans*.

### KNL2 interacts with CDC20.1 and APC10

To investigate the regulation of αKNL2 via ubiquitination, we selected UBC19 and UBC20, identified through Y2H library screening with αKNL2-N, as well as APC2, CUL1, and CUL3, identified via AP-MS as components of complexes precipitated with αKNL2-N. These candidates were chosen based on their established roles in ubiquitin-mediated regulation and cell cycle control, indicating their potential involvement in αKNL2 turnover. Interestingly, all these putative interactors were identified with αKNL2-N, supporting our previous hypothesis that the proteolytic degradation of αKNL2 may be regulated through this region (Lermontova et al. 2013). A bimolecular fluorescence complementation (BiFC) assay was performed to validate these interactions. The αKNL2, αKNL2-N, and αKNL2-C were fused to the N-terminus of Venus (VEN_n_), while the selected proteins were fused to the C-terminus of Venus (VEN_c_), and vice versa. αKNL2/αKNL2-N^VENn^ exhibited interactions with UBC19/20^VENc^ and APC2^VENc^, whereas αKNL2-C^VENn^ interacted only with APC2^VENc^. In all cases, fluorescence resided in the nucleoplasm though occasionally also in the nucleolus and small nuclear speckles (Supplementary Fig. S5A). Similar results were obtained when the direction of VENUS fusion was changed (Supplementary Table S1). Interestingly, the number of fluorescent nuclei increased significantly when interactions between αKNL2/αKNL2-N and their binding proteins were scored after treatment with a proteasome inhibitor MG115 (Supplementary Fig. S5B), which is in line with the previous results showing that the proteasome degradation pathway regulates αKNL2.

To further confirm the interaction between αKNL2 fragments and proteins involved in the ubiquitin-proteasome pathway, a Y2H cotransfection assay was conducted. The yeast strains expressing αKNL2, αKNL2-N, or αKNL2-C^BD^ as bait and UBC19/UBC20^AD^ as prey did not grow on the selective triple dropout (TDO) medium. This suggests that UBC19 and UBC20 may interact with αKNL2 weakly or transiently, making their detection challenging in the direct transformation assay. However, interactions were confirmed between αKNL2-N^BD^ and APC2^AD^ (Supplementary Fig. S6A and Table S2). None of the yeast strains coexpressing the selected proteins with the empty vector grew on the selective medium except for a few constructs showing autoactivation of proteins (Supplementary Table S2). Moreover, neither of the 2 αKNL2 fragments nor the full-length αKNL2 exhibited any interaction with CUL1 or CUL3 in both interaction approaches (Supplementary Figs. S5A and S6B), implying that αKNL2 is regulated by the APC/C complex.

**Figure 3.**
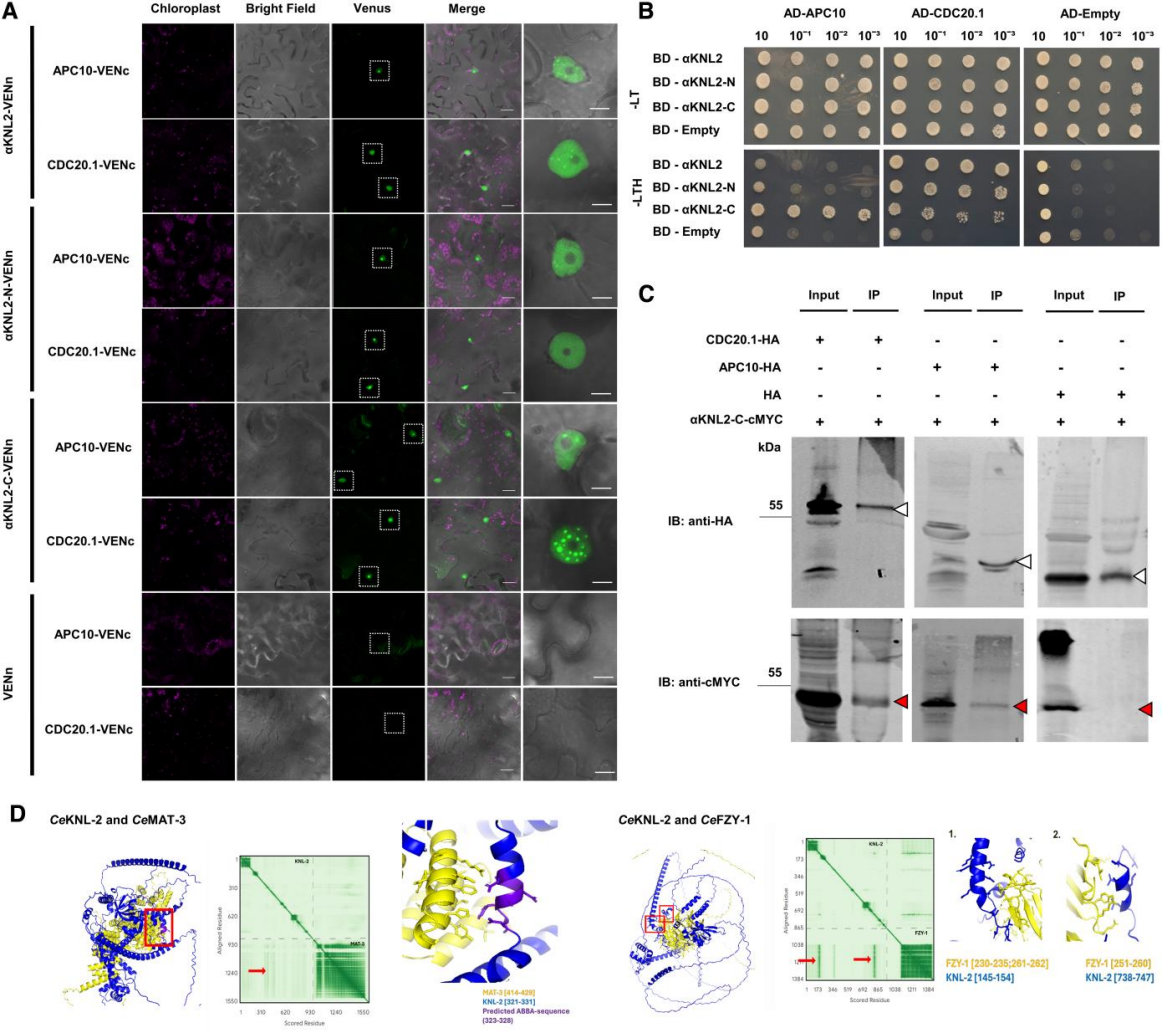
Direct interaction of KNL2 with APC/C proteins. **A)** BiFC analysis showing the interactions between αKNL2/αKNL2-N/αKNL2-C fused to VENn and APC10/CDC20.1 fused to VENc. Venus fluorescence in the nucleus was shown in white dotted boxes. Scale bars represent 50 *µ*m. The right panel displays an enlarged image of the corresponding BiFC signals in the nucleus, but may not always correspond to the exact nuclei shown in the overview panel. Scale bars represent 5 *µ*m. **B)** Y2H assay testing interactions between αKNL2 (bait) and APC10/CDC20.1 (prey). Zygotes expressing both prey and bait were selected on -LT medium (double dropout: YNB without leucine and tryptophan). Protein–protein interactions were assessed on -LTH medium (TDO: YNB without leucine, tryptophan, and histidine). The strength of the protein–protein interactions was evaluated using a drop dilution assay. AD, activation domain; BD, binding domain. **C)** Co-IP interactions between αKNL2 and CDC20.1/APC10. *N. benthamiana* leaves were infiltrated with constructs containing CDC20.1-HA and αKNL2-C-cMYC (Lanes 1, 2), APC10-HA and αKNL2-C-cMYC (Lanes 3, 4), as well as HA and αKNL2-C-cMYC (Lanes 5, 6). Total protein extracts were precipitated with HA magnetic beads, and the samples were analyzed before (input) and after (IP) immunoprecipitation by immunoblotting with HA and c-MYC antibodies. The triangle (▵) marks the MW of CDC20.1, APC10, and empty control while the red arrowhead (▴) indicates the MW of αKNL2-C. IB, immunoblot; IP, immunoprecipitation. **D)** AlphaFold 3 prediction between *C. elegans* KNL-2 (blue) and MAT-3 or FZY-1 (yellow). The left panels present the predictions of the complexes formed between the protein pairs. Red boxes highlight the areas of interaction. The middle panels display heat maps of the interactions, with arrows indicating the specific sites of interaction. The right panels provide a close-up view of the red boxes in the left panels, highlighting the precise locations where the interaction is predicted. *Ce*MAT-3 (weakly) is predicted to interact with one of the predicted APC/C-specific degron motifs (purple), whereas *Ce*FZY-1 (strongly) predicted to interact with *Ce*KNL-2.

In Arabidopsis, the APC/C ligase, composed of 11 core subunits including APC2, which has been identified as an interactor of αKNL2, recognizes substrates via coactivator proteins such as APC10, CDC20.1, or CDH1.1 (da Fonseca et al. 2011). Therefore, BiFC direct interaction analysis was employed to test the interactions of αKNL2 with coactivators of APC/C. This analysis confirmed the interaction of αKNL2/αKNL2-N/αKNL2-C^VENn/VENc^ with CDC20.1^VENc/VENn^ and APC10^VENc/VENn^ in the nucleoplasm and some nuclear foci when CDC20.1/APC10 interact with αKNL2/αKNL2-C (Fig. 3A; Supplementary Table S1). Conversely, none of the αKNL2 fragments interacted with CDH1.1. Furthermore, Y2H assays revealed interactions of αKNL2/αKNL2-N/αKNL2-C^BD^ with CDC20.1^AD^ and αKNL2-C^BD^ with APC10^AD^. In contrast, no interactions were observed between αKNL2 fragments and CDH1.1, supporting the BiFC results (Supplementary Fig. S6 and Table S2). To further confirm the direct interaction between αKNL2 and APC/C proteins, a co-immunoprecipitation (Co-IP) assay was performed. Fusion proteins CDC20.1-HA or APC10-HA were coexpressed with αKNL2-C-cMYC, and the total proteins were precipitated by HA magnetic beads. Western blot analysis with anti-cMYC antibody revealed that αKNL2-C-cMYC was detectable when coexpressed with CDC20.1-HA or APC10-HA, but not with empty-HA (Fig. 3C). This finding is consistent with BiFC and Y2H results, which demonstrate a physical interaction between αKNL2 and APC/C coactivators, suggesting that the APC/C-CDC20 complex may facilitate αKNL2 degradation.

In line with the above results, IP data for *Ce*KNL-2 revealed the presence of APC/C complex proteins, including *Ce*MAT-1, *Ce*MAT-3, *Ce*EMB-27, and *Ce*FZY-1. Using AlphaFold 3, we predicted interactions between *Ce*KNL-2 and these selected candidate proteins. The results suggest that *Ce*KNL-2 interacts with *Ce*MAT-1, *Ce*EMB-27, and *Ce*MAT-3 (weak), through KEN and ABBA motifs, which are APC/C-specific degrons (Fig. 3D; Supplementary Fig. S7). Additionally, AlphaFold 3 indicates a strong interaction between *Ce*FZY-1 and *Ce*KNL-2 (Fig. 3D). These findings suggest that *Ce*KNL-2 may also be regulated via an APC/C-mediated degradation mechanism.

### αKNL2 is a substrate of the APC/C complex

**Figure 4.**
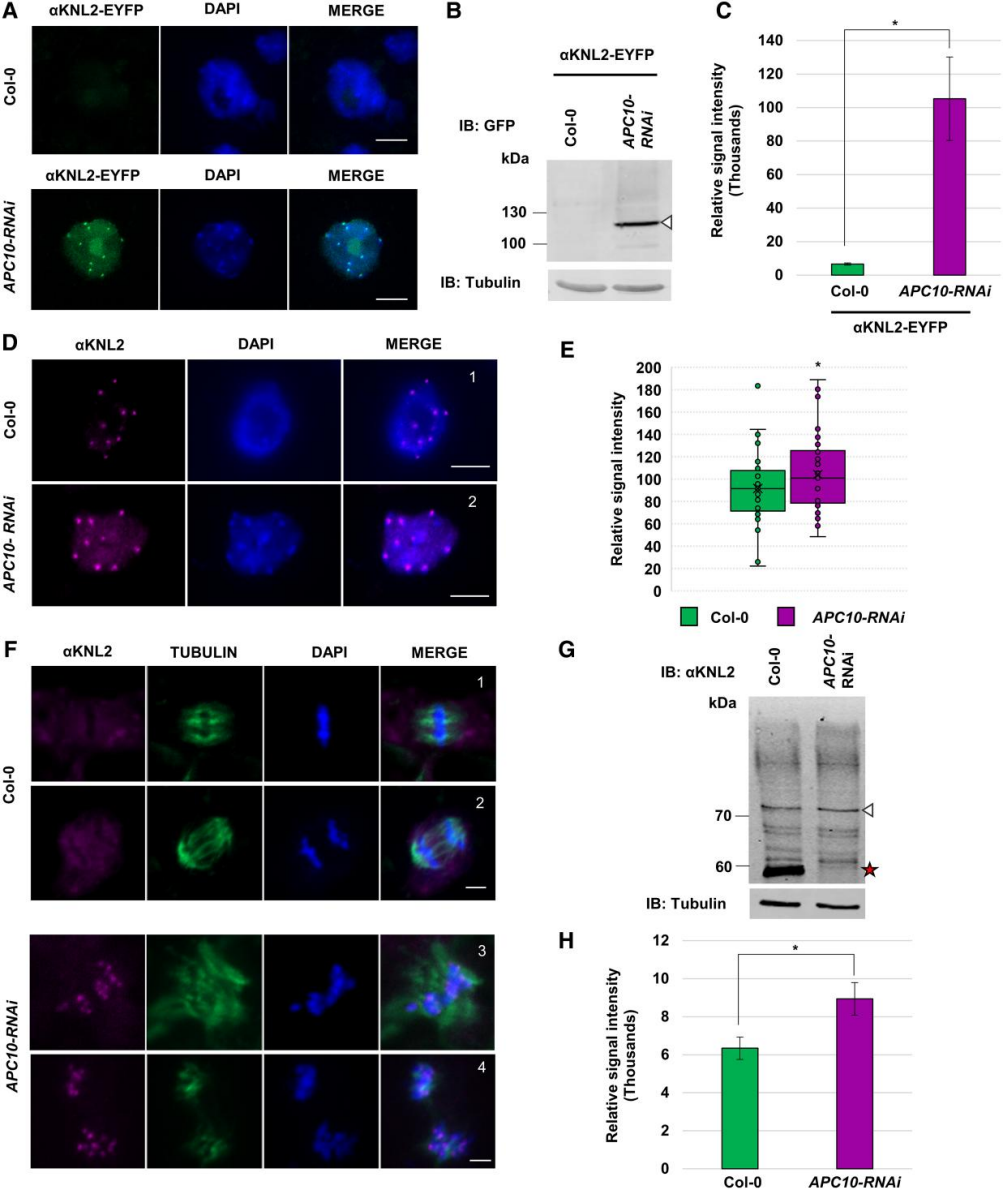
αKNL2 is a target of the APC/C complex in *A. thaliana.*  **A)** Immunostaining of meristematic nuclei from *APC10-RNAi* lines and wild-type plants transformed with the αKNL2-EYFP construct using anti-GFP antibodies. Scale bars represent 5 *µ*m. **B and C)** Analysis of αKNL2 protein levels in *APC10-RNAi* and wild-type plants expressing αKNL2-EYFP. **B)** Western blot analysis of nuclear protein extracts from *APC10-RNAi* and wild-type plants expressing αKNL2-EYFP, using an anti-GFP antibody. Tubulin was used as a loading control. The triangle symbol (▵) indicates the MW of αKNL2. **C)** Quantification of αKNL2 protein levels from **B)**, with band intensities normalized to tubulin. Data are presented as mean values ± SEM (*n* = 3). Significant differences between groups were assessed using Welch's *t*-test and are marked by * (*P* < 0.5). **D)** Immunostaining of meristematic nuclei of Arabidopsis wild-type (1) and the *APC10-RNAi* lines (2) using anti-αKNL2 antibodies. Scale bars are 5 *μ*m. **E)** Relative intensity measurements of αKNL2 signals on nuclei from *APC10-RNAi* and wild type. Boxplots show the distribution of fluorescence intensities (*n* = 35 per group). The centerline indicates the median, and box limits represent the upper and lower quartiles (Q1 and Q3); whiskers extend to 1.5× the interquartile range. Significant differences between groups were assessed using Welch's *t*-test and are indicated by * (*P* < 0.5). **F)** The mitotic localization of αKNL2 in root meristem tissues of wild-type (1,2) and *APC10-RNAi* (3,4) using αKNL2 antibodies (magenta). αKNL2 localizes to centromeres during mitosis in *APC10-RNAi*. DAPI-stained chromosomes are shown in blue, and anti-α-tubulin immunosignals are shown in green. (1, 3) metaphase; (2, 4) anaphase. Scale bars are 5 *μ*m. **G and H)** Analysis of endogenous αKNL2 protein levels in wild type and *APC10-RNAi*. **G)** Western blot analysis of nuclear protein extracts from wild type, and *APC10-RNAi* lines, detected using αKNL2-specific antibody. Tubulin was used as a loading control. The white triangle (▵) indicates the MW of αKNL2, and the red star (★) denotes the absence of degradation products. **H)** Quantification of αKNL2 protein levels from **G)**, with band intensities normalized to tubulin. Data are shown as mean values ± SEM (*n* = 3). Significant differences between groups were assessed using Welch's *t*-test and are indicated by * (*P* < 0.5).

If αKNL2 is a target of the APC/C, plants with impaired APC/C activity are expected to accumulate more αKNL2 than wild-type plants. Therefore, to assess αKNL2 protein levels, the αKNL2-EYFP construct was introduced into the hypomorphic RNAi lines targeting the APC/C subunit (APC10) (Marrocco et al. 2009; Pan et al. 2023), which exhibited reduced *APC10* mRNA expression (Supplementary Fig. S8A). The immunostaining with an anti-GFP antibody on *APC10-RNAi* lines expressing the αKNL2-EYFP construct revealed distinct centromere-specific αKNL2-EYFP signals in the nuclei of meristematic root tips. In contrast, wild-type plants transformed with the same construct showed no detectable fluorescence (Fig. 4A). Supporting these observations, IB analysis showed a higher accumulation of αKNL2-EYFP in the *APC10-RNAi* lines compared to the wild-type background (Fig. 4, B and C). These findings, together with similar *αKNL2* transcript levels in both backgrounds (Supplementary Fig. S8B), indicate that the APC/C complex regulates αKNL2 posttranslationally. Additionally, CENH3 gene expression and protein levels were mildly elevated in *APC10-RNAi* compared to wild-type plants (Supplementary Fig. S8, B to D).

Next, to determine the stability of endogenous αKNL2 in *APC10-RNAi*, immunostaining with αKNL2-specific antibodies was performed on the meristematic root tip nuclei from both *APC10-RNAi* and wild-type plants. The analysis showed a 1.2-fold increase in αKNL2 signal intensity compared to the wild-type, suggesting enhanced stability in the *APC10-RNAi* lines (Fig. 4, D and E). Previously, Lermontova et al. (2013) reported that αKNL2 protein is absent during the early stages of mitosis. In contrast to wild-type plants, in the *APC10-RNAi* line, αKNL2 begins to accumulate at the centromeres from early metaphase to anaphase stages (Fig. 4F; Supplementary Fig. S9A). However, only 20% to 26% of mitotic stages showed αKNL2 accumulation at centromeres, in contrast to the higher incidence of mitotic defects, which were seen in 40% to 45% of *APC10-RNAi* cells (Supplementary Fig. S9, B and C). Consistent with this observation, western blot analysis using αKNL2-specific antibodies on nuclear protein extracts revealed increased accumulation of αKNL2, with no detection of lower MW bands in the *APC10-RNAi* lines compared to wild type (Fig. 4, G and H). This indicates that αKNL2 degradation is impaired in APC/C mutants. Together, these findings suggest that αKNL2 is a target of the APC/C complex, which mediates its degradation during the early mitotic phase.

### APC/C interacts with αKNL2 via D-box1 and mediates its degradation

**Figure 5.**
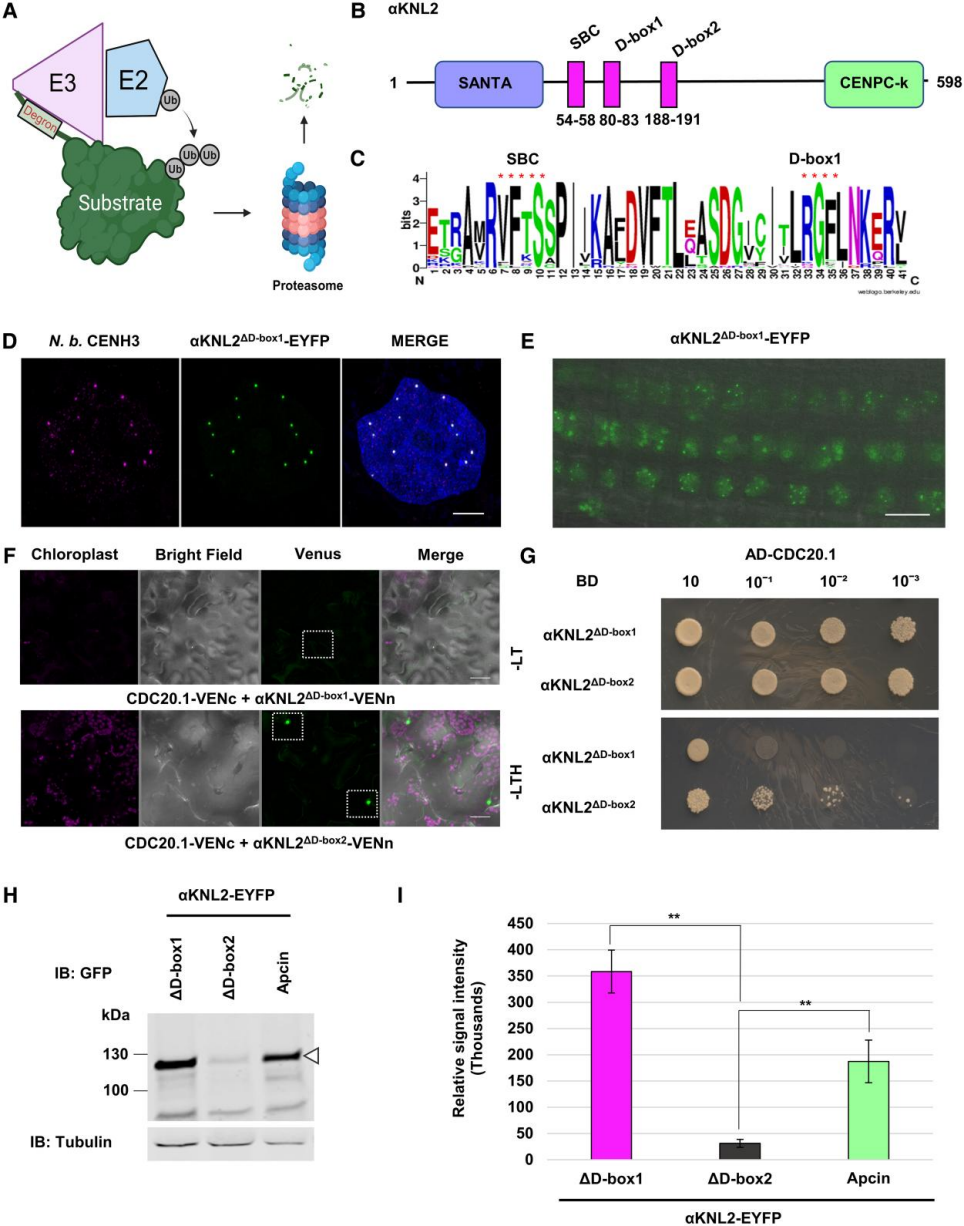
The APC/C D-box1 region at the N-terminus is a functional degron of αKNL2. **A)** Schematic of the ubiquitination process involving E2 conjugation enzymes and E3 ligases, marking substrates with degrons for proteasomal degradation. **B)** Diagram of αKNL2 structure showing SBC, D-box1, and D-box2 degrons. **C)** WebLogo alignment showing conserved αKNL2 sequences (48 to 88 amino acids) in Brassicales, highlighting conserved SBC (VFTSS) and D-box1 (RGFL) domains (red asterisks). **D)** Superresolution SIM image showing colocalization of αKNL2^ΔD-box1^-EYFP fluorescence signals (green) with CENH3 (magenta) in *N. benthamiana* leaves. Scale bars represent 5 *µ*m. **E)** The localization pattern of αKNL2^ΔD-box1^-EYFP in Arabidopsis root tips. Scale bars represent 10 *µ*m. **F)** BiFC analysis shows no interaction between αKNL2^ΔD-box1^-VENn and CDC20.1-VENc (no fluorescence), while αKNL2^ΔD-box2^-VENn and CDC20.1-VENc show nuclear fluorescence. Scale bars represent 5 *µ*m. **G)** Y2H assay shows interaction of αKNL2^ΔD-box2^ but not αKNL2^ΔD-box1^ with CDC20.1. Zygotes were selected on -LT medium (double dropout), and interactions were evaluated on -LTH medium (TDO) using a drop dilution assay. AD, activation domain; BD, binding domain. **H)** Western blot of proteins from plants expressing αKNL2^ΔD-box1^-EYFP, αKNL2^ΔD-box2^-EYFP, and αKNL2-EYFP treated with Apcin, detected with anti-GFP antibody. The triangle (▵) represents αKNL2 MW. **I)** Quantification of protein levels from **H)**, normalized to tubulin, shown as mean ± SEM (*n* = 3). Significant differences between groups were assessed using Welch's *t*-test and are indicated by ** (*P* < 0.05).

Ubiquitin E3 ligases recognize their substrates through specific degrons for the ubiquitination and degradation of the substrate via the proteasomal pathway (Fig. 5A). Interestingly, αKNL2 contains 3 predicted degrons: 2 destruction boxes (D-box1, D-box2) and SPOP-binding consensus (SBC) (Fig. 5B). The SBC is a domain where the Cullin E3 ligase actively binds. To understand the diversification and evolution of the *αKNL2* gene, sequences of αKNL2 homologs identified in Brassicales genomes were aligned (Zuo et al. 2022). The alignment analysis demonstrated that the D-box1 (80 to 83 aa) and SBC (54 to 58 aa) regions of αKNL2 are conserved in Brassicales, unlike the D-box2 (188 to 191 aa) (Fig. 5C). To determine which degron is responsible for the degradation of αKNL2 and its interaction with APC/C, mutant versions of αKNL2 were generated by deleting the identified degrons (αKNL2^ΔD-box1^, αKNL2^ΔD-box2^, and αKNL2^ΔSBC^). The resulting constructs were fused to EYFP and expressed under the control of a 35S promoter. First, *N. benthamiana* leaves were infiltrated with *A. tumefaciens* containing these constructs and the results demonstrated that αKNL2^ΔD-box1^-EYFP localized in the nucleoplasm and formed small speckles corresponding to centromeres as shown by colocalization with *N. benthamiana* CENH3 (Fig. 5D). Conversely, fewer or no fluorescent signals were found for αKNL2^ΔD-box2^-EYFP and αKNL2^ΔSBC^-EYFP. The number of fluorescent nuclei was quantified in 80 mm^2^ leaf discs of *N. benthamiana*, and the results indicated that the αKNL2^ΔD-box1^-EYFP mutant exhibited a significantly greater number of fluorescent nuclei compared to the αKNL2^ΔD-box2^-EYFP, αKNL2^ΔSBC^-EYFP, and αKNL2-EYFP variants (Supplementary Fig. S10, A to C).

Based on these results, we generated Arabidopsis plants stably expressing αKNL2^ΔD-box1^-EYFP and αKNL2^ΔD-box2^-EYFP fusion constructs to study the localization pattern of mutagenized αKNL2 protein variants. The results were similar to those observed in *N. benthamiana*, where centromere-specific signals were detected in the root tips of at least 3 independent T2 transgenic lines expressing the αKNL2^ΔD-box1^-EYFP in contrast to T2 lines expressing αKNL2^ΔD-box2^-EYFP and αKNL2^ΔSBC^-EYFP (Fig. 5E; Supplementary Fig. S10D). Further, to assess whether the deletion of D-boxes affects the interaction of αKNL2 with the APC/C activator CDC20.1, BiFC and Y2H assays were conducted using mutated αKNL2 and CDC20.1. Our results indicated that the deletion of D-box1, but not D-box2, abolished the interaction between αKNL2 and CDC20.1 in both assays (Fig. 5, F and G). Thus, these results indicate that APC/C interacts with the conserved D-box1 region of αKNL2, which acts as a functional degron for the proteasomal degradation of αKNL2 in *A. thaliana*.

Apcin is a novel cell-permeable APC/C inhibitor that binds to CDC20 and competitively inhibits the ubiquitination of D-box-containing substrates (Gao et al. 2018). This drug was recently shown to act as a CDC20 inhibitor in plants as well (Pan et al. 2023). Treatment of Arabidopsis seedlings stably expressing αKNL2-EYFP with Apcin resulted in more pronounced αKNL2-specific signals compared to the DMSO control, and similar results were observed in *N. benthamiana* (Supplementary Fig. S11, A to D). Additionally, αKNL2 protein levels were compared in total protein extracts from *N. benthamiana* leaves expressing the D-box deletion constructs of αKNL2 fused with EYFP and unmutagenized αKNL2-EYFP treated with Apcin inhibitor. IB analysis revealed that αKNL2 protein levels were higher in samples with the D-box1 deletion and Apcin inhibitor treatment compared to those with the D-box2 deletion (Fig. 5, H and I). Next, an IP experiment was conducted employing a GFP trap and immunoblotted with anti-GFP or anti-ubiquitin antibody. Western blot analysis using anti-GFP on input samples from αKNL2^ΔD-box2^-EYFP and αKNL2^ΔSBC^-EYFP showed elevated αKNL2 protein levels when treated with bortezomib. In contrast, protein levels in αKNL2^ΔD-box1^-EYFP and αKNL2-EYFP samples treated with Apcin remained unaltered, likely due to the inhibition of αKNL2 interaction with the APC/C ligase. Interestingly, the αKNL2^ΔSBC^-EYFP sample treated with bortezomib exhibited polyubiquitination comparable to that of αKNL2-EYFP (Fig. 1E), whereas αKNL2^ΔD-box2^-EYFP displayed partial polyubiquitination. In contrast, αKNL2^ΔD-box1^-EYFP and αKNL2-EYFP samples treated with Apcin showed reduced ubiquitin detection (Supplementary Fig. S12). These results suggest that D-box1 removal or Apcin treatment impairs αKNL2 ubiquitination by the APC/C-CDC20.

### Ubiquitination at lysine 336 and 339 directs αKNL2 degradation by the proteasome

**Figure 6.**
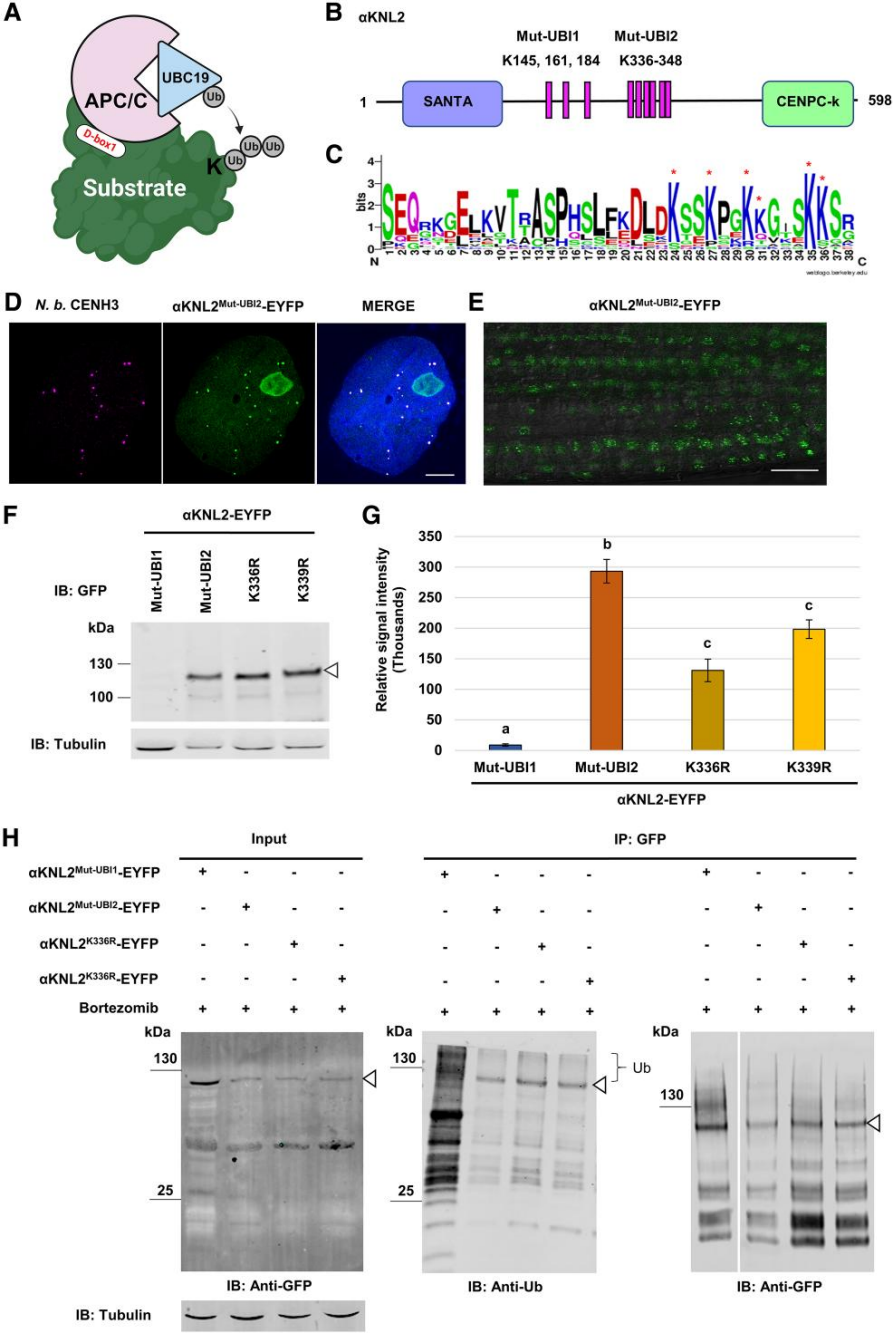
αKNL2 ubiquitination sites and their role in protein stability. **A)** Model illustrating the ubiquitination process, where ubiquitin is transferred to lysine residues on a substrate after its interaction with the E2–E3 complex. **B)** Schematic representation of αKNL2 protein containing 3 conserved lysine (K) residues at positions 145, 161, and 184 (Mut-UBI1 site) and 6 conserved K residues at positions 336, 339, 342, 343, 347, and 348 (Mut-UBI2 site) that were substituted with arginine. **C)** The WebLogo show the relative frequency of each amino acid at both Mut-UBI sites based on the alignment of KNL2 homologs from Brassicales species. Asterisks indicate the positions of highly conserved KNL2 lysine residues. **D)** SIM image showing the colocalization of αKNL2^Mut-UBI2^-EYFP (green) with CENH3 (magenta) in *N. benthamiana* leaves, indicating centromere-specific signals. Scale bars represent 5 *µ*m. **E)** Expression pattern of αKNL2^Mut-UBI2^-EYFP in Arabidopsis root tips. Scale bars represent 10 *µ*m. **F and G)** Analysis of αKNL2 protein levels in *N. benthamiana* leaves transiently expressing ubiquitin site–mutagenized variants of αKNL2. **F)** Western blot analysis of total protein extracts from leaves expressing αKNL2^Mut-UBI1^-EYFP, αKNL2^Mut-UBI2^-EYFP, αKNL2^K336R^-EYFP, and αKNL2^K339R^-EYFP, using a monoclonal anti-GFP antibody. The triangle symbol (▵) denotes the MW of αKNL2. **G)** Quantification of αKNL2 protein levels among the different ubiquitin site–mutagenized constructs **F)**. Protein levels were normalized to tubulin. Data are presented as mean ± SEM (*n* = 3). Significant differences are marked by lowercase letters based on ANOVA and Tukey's multiple comparison tests (*P* < 0.05). **H)** Ubiquitination assay of ubiquitin site–mutagenized αKNL2 variants. Total protein extracts from plants expressing αKNL2^Mut-UBI1^-EYFP, αKNL2^Mut-UBI2^-EYFP, αKNL2^K336R^-EYFP, and αKNL2^K339R^-EYFP, treated with bortezomib, were immunoprecipitated using a GFP trap. Input samples were immunoblotted with monoclonal anti-GFP antibody (left panel), while immunoprecipitated samples were probed with monoclonal antibodies against ubiquitin (Ub) (center panel) or GFP (right panel). Tubulin was used as a loading control. The triangle symbol (▵) (central and right panel) indicates the size of mutagenized αKNL2 in fusion with EYFP, and the bracket marks the ubiquitinated form of αKNL2 (middle panel). IB, immunoblot; IP, immunoprecipitation.

We assumed that αKNL2 requires ubiquitination by the APC/C at specific lysine (K) residues for its degradation by the proteasome (Fig. 6A). Therefore, we aimed to identify the potential ubiquitin binding sites of αKNL2 protein using in silico analysis. Nine highly conserved lysine residues (K145, K161, K184, K336, K339, K342, K343, K347, K348) at the N-terminal part of αKNL2 were found by sequence alignment of αKNL2 homologs from Brassicales genomes (Fig. 6, B and C). Two mutant variants of *A. thaliana* αKNL2 containing simultaneous substitutions of lysine to arginine (K to R) either at 3 positions K145, K161, and K184 (αKNL2^Mut-UBI1^) or at 6 positions in the K336-K348 region (αKNL2^Mut-UBI2^) were generated. Both variants were fused to EYFP and subsequently expressed in *N. benthamiana* plants under the 35S promoter. The localization analysis of the mutagenized αKNL2 variants demonstrated the nuclear and centromere-specific signal of the αKNL2^Mut-UBI2^-EYFP and its colocalized with CENH3 (Fig. 6D), whereas αKNL2^Mut-UBI1^-EYFP did not show any fluorescence like αKNL2-EYFP (control). These findings suggest that the lysine residues in the K336-K348 region could be involved in the polyubiquitination of αKNL2.

To determine the precise ubiquitination site(s), which enabled observation of αKNL2^Mut-UBI2^-EYFP in centromeres, site-directed mutagenesis individually targeting 1 of 6 lysine residues in αKNL2^Mut-UBI2^-EYFP was carried out and the obtained mutagenized constructs were introduced into *N. benthamiana* leaves. As a result, only 2 variants, αKNL2^K336R^-EYFP and αKNL2^K339R^-EYFP, displayed nuclear and centromeric localization (Supplementary Fig. S13, A and B). By counting the number of fluorescent nuclei in 80 mm^2^ leaf discs of *N. benthamiana*, αKNL2^Mut-UBI2^-EYFP, αKNL2^K336R^-EYFP, and αKNL2^K339R^-EYFP showed a higher number of fluorescent nuclei compared to other ubiquitin-mutagenized variants (Supplementary Fig. S13C). To investigate the in vivo localization patterns of the ubiquitin-mutagenized αKNL2 protein variants, we generated stable transformants of Arabidopsis expressing the αKNL2^Mut-UBI2^-EYFP, αKNL2^K336R^-EYFP, and αKNL2^K339R^-EYFP fusion constructs. Analysis of the root tip of at least 3 T2-independent transgenic Arabidopsis lines expressing the αKNL2^Mut-UBI2^-EYFP, αKNL2^K336R^-EYFP, and αKNL2^K339R^-EYFP showed a centromeric localization pattern for αKNL2 (Fig. 6E; Supplementary Fig. S13, D and E).

To determine if mutations at potential ubiquitination sites can prevent αKNL2 protein degradation and lead to its accumulation, we conducted a western blot analysis using anti-GFP antibodies on total protein extracts from *N. benthamiana* leaves transiently expressing the mutated constructs. This analysis revealed higher αKNL2 protein levels in αKNL2^Mut-UBI2^-EYFP, αKNL2^K336R^-EYFP, and αKNL2^K339R^-EYFP compared to the αKNL2^Mut-UBI1^-EYFP samples (Fig. 6, F and G). In addition, to compare the level of ubiquitination of the mutagenized αKNL2 protein variants, an IP experiment was performed. Western blot against anti-GFP of input samples from αKNL2^Mut-UBI1^-EYFP showed high accumulation of αKNL2 protein levels when treated with bortezomib, whereas αKNL2^Mut-UBI2^-EYFP, αKNL2^K336R^-EYFP, and αKNL2^K339R^-EYFP protein levels were unchanged due to the inhibition of KNL2 ubiquitination. Further, immunoprecipitated samples were analyzed by western blot using anti-ubiquitin and anti-GFP antibodies showed reduced ubiquitination in the αKNL2^Mut-UBI2^-EYFP as well as the K336R and K339R variants, compared to αKNL2^Mut-UBI1^-EYFP (Fig. 6H), supporting that K336 and K339 are likely the receptor sites in αKNL2 for ubiquitination by the APC/C-CDC20.

### Nondegradable Arabidopsis αKNL2 affects fertility and mitosis

**Figure 7.**
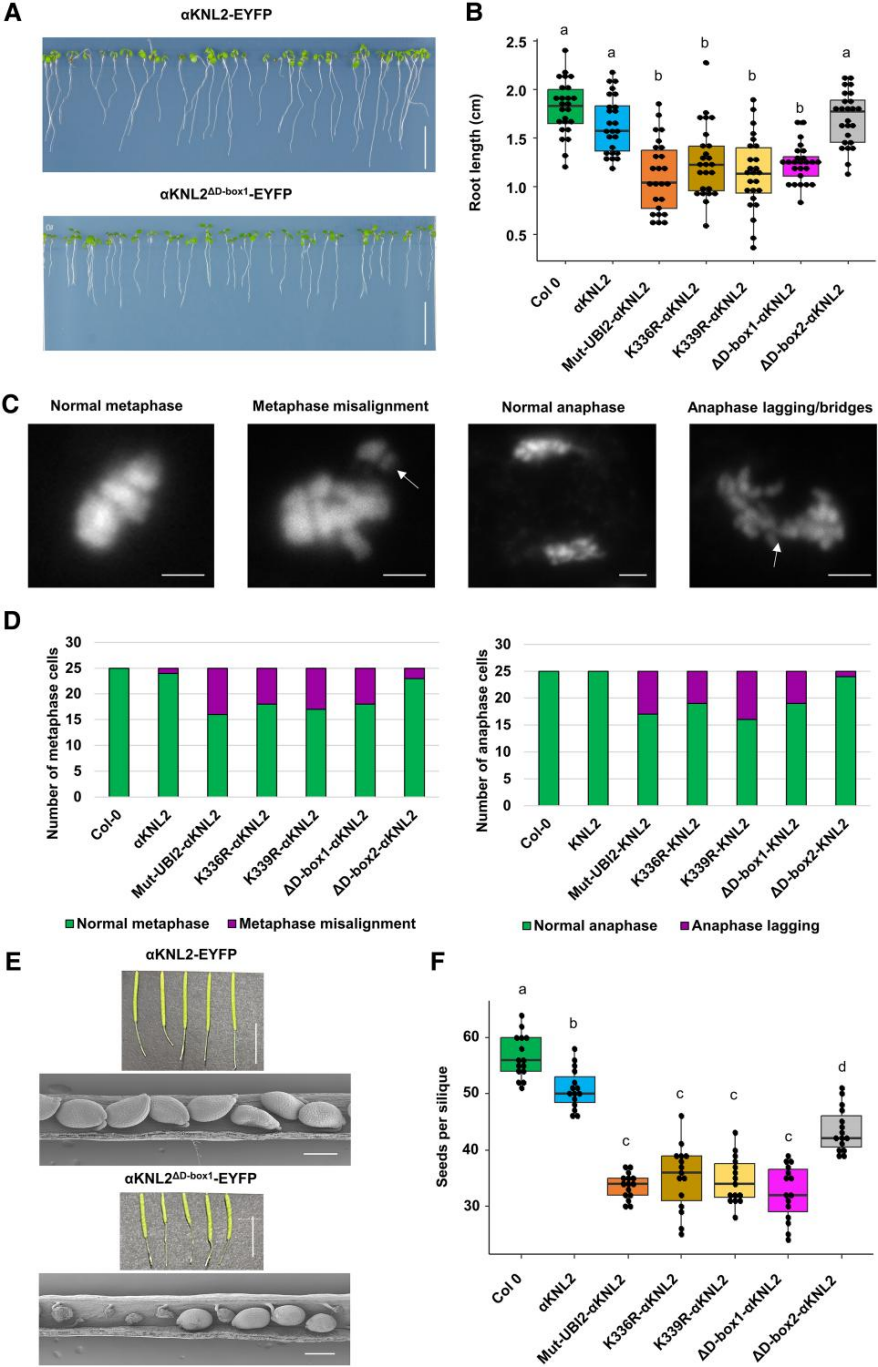
The phenotype of degradation-resistant αKNL2 lines. **A)** Root growth phenotype of 7-day-old Arabidopsis seedlings expressing the αKNL2^ΔD-box1^-EYFP and αKNL2-EYFP constructs. Scale bars represent 1 cm. **B)** Box plot illustrating the primary root length of wild-type, αKNL2-EYFP, and degradation-resistant lines of αKNL2 grown for 7 d on 0.5× MS medium containing 1% sucrose. Data represent 25 seedlings per construct. The centerline indicates the median, and box limits represent the upper and lower quartiles (Q1 and Q3); whiskers extend to 1.5× the interquartile range. Significant differences are indicated by lowercase letters according to ANOVA followed by Tukey's multiple comparison tests (*P* < 0.05). **C)** Mitotic metaphases and anaphases of wild-type and transgenic Arabidopsis plants expressing either αKNL2-EYFP or degradation-resistant αKNL2 variants, showing misaligned and lagging chromosomes (indicated by white arrows). Scale bars represent 5 *µ*m. **D)** Quantification of abnormal metaphases and anaphases in plants expressing degradation-resistant αKNL2 variants, in comparison to wild-type and αKNL2-EYFP-expressing plants. For each variant, 30 metaphase and anaphase cells were examined. In lines expressing degradation-resistant αKNL2, 24% to 32% of metaphases were misaligned and 28% to 36% of anaphases exhibited bridged and/or lagging chromosomes. **E)** Silique size comparison between αKNL2^ΔD-box1^-EYFP- and αKNL2-EYFP-expressing plants (upper panel). Scale bars represent 1 cm. Scanning electron microscopy images of respective siliques are shown (lower panel). Scale bars represent 20 *µ*m. **F)** Analysis of seed setting in Arabidopsis wild-type and transgenic plants expressing either αKNL2-EYFP or degradation-resistant αKNL2 variants. Box plot showing the average number of seeds per silique for 15 plants per construct, with 10 siliques analyzed per plant. The centerline indicates the median, while the box limits denote the lower (Q1) and upper (Q3) quartiles. Whiskers extend to 1.5× the interquartile range. Significant differences are marked by lowercase letters based on ANOVA and Tukey's multiple comparison tests (*P* < 0.05).

We found that the deletion of D-box1 or the mutation of ubiquitin sites (UBI2, K336R, K339R) causes an increase in αKNL2 levels by inhibiting its degradation (degradation-resistant αKNL2 lines). To differentiate between transcriptional and posttranslational regulation, we analyzed total *αKNL2* transcript levels in plants expressing unmodified αKNL2-EYFP, degradation-resistant αKNL2-EYFP variants, and wild-type plants. The results revealed that *αKNL2* mRNA levels were similar between αKNL2-EYFP lines lacking detectable fusion protein fluorescence and degradation-resistant lines displaying clear fluorescence signals. However, both exhibited higher *αKNL2* mRNA levels than wild-type plants (Supplementary Fig. S14A). These findings suggest that αKNL2 accumulation is primarily regulated at the posttranslational level rather than through transcriptional control. To test whether overaccumulation of αKNL2 protein by impaired degradation impacts plant growth and development, transgenic Arabidopsis lines expressing the αKNL2^Mut-UBI2^-EYFP, αKNL2^K336R^-EYFP, αKNL2^K339R^-EYFP, αKNL2^ΔD-box1^-EYFP, and αKNL2^ΔD-box2^-EYFP mutant constructs were tested. The root growth of 7-day-old Arabidopsis seedlings of lines expressing degradation-resistant αKNL2 was compared with seedlings expressing αKNL2-EYFP and nontransgenic wild-type seedlings. The αKNL2^Mut-UBI2^-EYFP, αKNL2^K336R^-EYFP, αKNL2^K339R^-EYFP, and αKNL2^ΔD-box1^-EYFP-expressing lines showed root length reduced by about 60% when compared to αKNL2^ΔD-box2^-EYFP, αKNL2-EYFP, and wild-type plants (Fig. 7, A and B; Supplementary Fig. S14B).

Mitosis in root tip meristems of plants over accumulating αKNL2 revealed abnormalities such as 24% to 32% of misaligned metaphases and 28% to 36% of anaphases (out of 25 cells) with bridges and/or lagging chromosomes (Fig. 7, C and D). We hypothesized that these defects might result from impaired centromere function or disruptions in spindle assembly checkpoint (SAC) complex. To test this, we analyzed the expression of key centromere and *SAC* genes (*CENH3*, *MAD2*, *BUBR1*, and *BUB3.1*) using RT-qPCR in seedlings of wild-type plants and those expressing either unmodified or degradation-resistant αKNL2-EYFP variants. Gene expression levels were similar between wild-type plants and unmodified αKNL2-EYFP or αKNL2^ΔD-box2^-EYFP constructs. In contrast, plants expressing degradation-resistant αKNL2-EYFP variants showed a slight increase in *CENH3* transcript levels and a pronounced increase in *SAC* gene transcripts (Supplementary Fig. S14C). Additionally, we assessed CENH3 protein levels through immunostaining, which also revealed a slight increase in CENH3 levels in degradation-resistant αKNL2-EYFP variants compared to αKNL2-EYFP plants (Supplementary Fig. S14, D and E). This upregulation of kinetochore components might contribute to the mitotic defects observed in plants with αKNL2 accumulation.

In addition, we noticed differences in the vegetative growth and development of the plants expressing degradation-resistant αKNL2 compared to the αKNL2^ΔD-box2^-EYFP and αKNL2-EYFP (Supplementary Fig. S14, F and G). These plants also displayed shorter siliques and reduced seed setting per silique. Upon expression of degradation-resistant αKNL2, the silique size was reduced compared to αKNL2^ΔD-box2^-EYFP and αKNL2-EYFP-expressing lines. In 150 siliques of the lines expressing degradation-resistant αKNL2, such as αKNL2^Mut-UBI2^-EYFP, αKNL2^K336R^-EYFP, αKNL2^K339R^-EYFP, and αKNL2^ΔD-box1^-EYFP, an average of 40.5%, 37.9%, 38.8%, and 42.6% of seeds were aborted, respectively (Fig. 7, E and F; Supplementary Fig. S14H). Thus, the expression of degradation-resistant αKNL2 affects plant growth, mitosis, and fertility.

## Discussion

**Figure 8.**
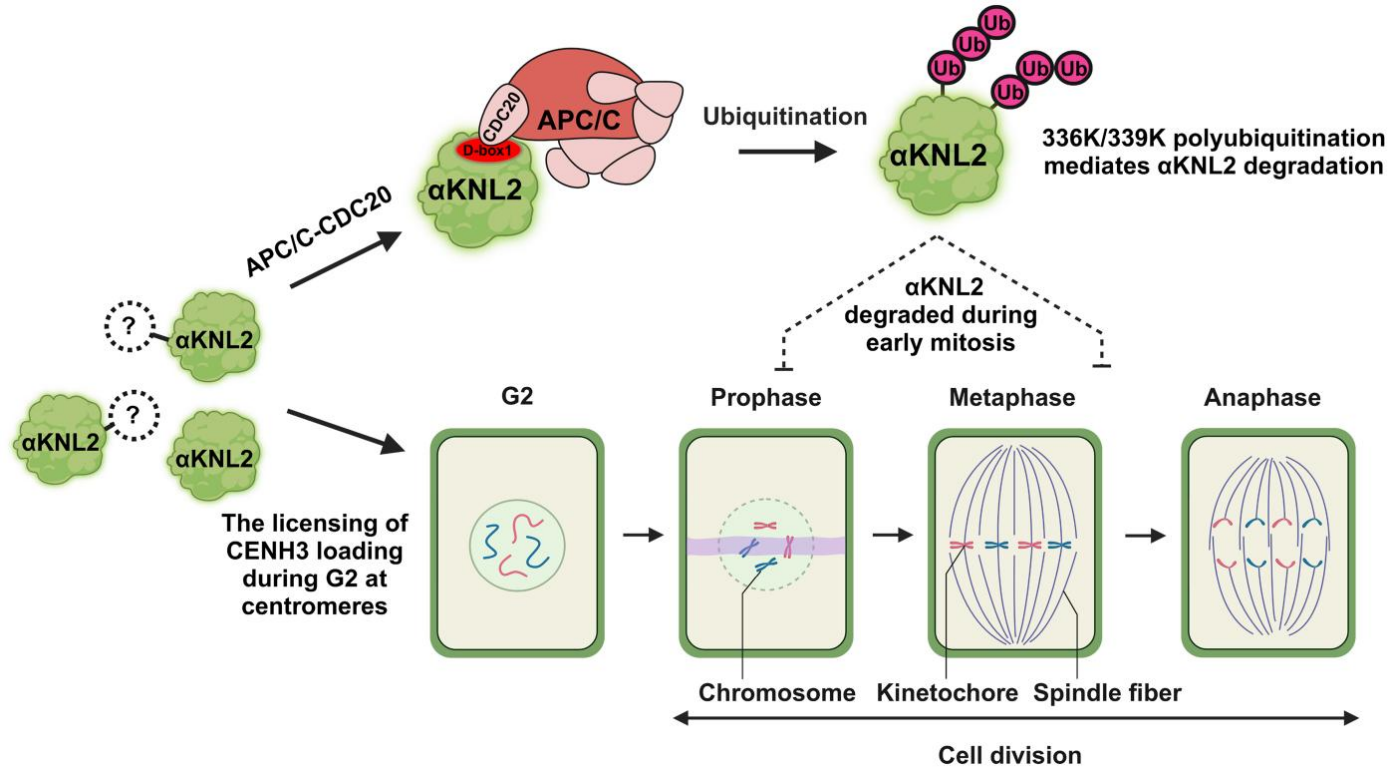
Model of APC/C-mediated ubiquitination and degradation of αKNL2 in Arabidopsis. When αKNL2 accumulates excessively, the APC/C E3 ubiquitin ligase, activated by E1 and E2 enzymes, targets it through the D-box1 motif (shown in red). This recognition triggers polyubiquitination at lysine residues 336 and 339, directing the protein to proteasomal degradation (upper part). αKNL2 is shown in green, while white circles with question marks indicate unknown regulatory mechanisms. Ubiquitin molecules are depicted as dark pink circles labeled “Ub,” and the APC/C–CDC20 complex is shown in light red-pink. During early mitosis, αKNL2 is also degraded through the APC/C–CDC20 pathway (dashed inhibitory arrows), ensuring proper SAC function and promoting cell cycle progression (lower part). This model was created using BioRender.com.

CENH3, a centromeric histone H3 variant, is crucial for kinetochore assembly, maintenance, and function during cell division. Excessive accumulation of kinetochore proteins significantly affects chromosome segregation and genome stability (Amato et al. 2009). Therefore, it is crucial to tightly regulate the levels of CENH3 and kinetochore components, which can be achieved through controlled protein degradation. αKNL2 is an essential kinetochore protein that plays a crucial role in CENH3 loading and cell division. In our current study, we uncovered molecular mechanisms that regulate the accumulation of αKNL2 via the ubiquitin-proteasome pathway. APC/C^CDC20^ E3 ligase complex facilitates the ubiquitination of αKNL2 through its D-box1, leading to the polyubiquitination and subsequent degradation of αKNL2 (Fig. 8). In contrast to CENH3, which remains stably associated with centromeres throughout the cell cycle, αKNL2 localizes to both the centromeres and nucleoplasm during interphase but disappears during mitosis (Lermontova et al. 2013). In our study, we showed that αKNL2 levels are regulated in a cell cycle-dependent manner through targeted degradation mediated by APC/C complex. In *C. elegans*, KNL-2 is associated with centromeres throughout the cell cycle (Maddox et al. 2007). However, complete removal of KNL-2 from chromatin occurs in the germline at the mitosis-to-meiosis transition, and this removal has previously been linked to ubiquitin-mediated degradation (Mohammad et al. 2018).

Protein degradation is crucial for many cellular processes and involves a complex network of proteolytic pathways. Among these, the UPS is the most utilized pathway in both the nucleus and cytosol. Interestingly, our findings demonstrate that αKNL2 is degraded by the UPS, as indicated by the effects of 26S proteasomal inhibitors, MG115 and bortezomib. Western blotting with an anti-ubiquitin antibody revealed high MW bands (corresponding to poly-Ub conjugated forms), indicating that αKNL2 is polyubiquitinated. Various types of ubiquitin chain formations can lead to substrate polyubiquitination, with Lys-48-linked polyubiquitination typically targeting proteins for degradation via the 26S proteasome.

Identification of proteins that interact with KNL2 is necessary for deepening our understanding of kinetochore functions in plants and animals. By analyzing the interaction networks of Arabidopsis αKNL2 and *Ce*KNL-2, we identified several orthologous proteins associated with the ubiquitin-proteasome pathway that potentially interact with KNL2, further supporting evidence of its degradation in plants and animals. Reactome enrichment analysis revealed that αKNL2 and *Ce*KNL-2 interactors are mostly involved in similar pathways, including protein translation modifications, metabolism, RNA Pol II transcription, and the cell cycle. These findings suggest a conserved mechanism of KNL2 regulation across species. In addition, GO analysis of αKNL2 and *Ce*KNL-2 interactors showed that proteins precipitated with KNL2 belong to functional categories such as cellular transport, nucleoplasmic transport, stress response, and development. This agrees with the proposed involvement of the SANTA domain of KNL2 in protein–protein interactions (Zhang et al. 2006). Moreover, GO analysis showed that KNL2 interactors demonstrated their role in biological processes already known to involve cell division and kinetochore components in *A. thaliana* and *C. elegans*. In Arabidopsis, 6 interactors were identified in both Y2H and AP-MS assays, including RADIATION SENSITIVE23 (RAD23A/D), heat-shock chaperones (DJA5/6), Calnexin homolog 1, and Voltage-dependent anion-selective channel 1 (VDAC1). In Arabidopsis, the nuclear-enriched RAD23 proteins bind ubiquitin conjugates, particularly those linked through Lys-48 chain formation, and associate with the ubiquitin receptor of the 26S proteasome system (Farmer et al. 2010). These findings strongly support our conclusion that αKNL2 undergoes polyubiquitination and is degraded via the UPS in Arabidopsis. The interaction of αKNL2 with DJA5/6, Calnexin homolog 1, and VDAC1 may be related to the necessity of αKNL2 protein transport in plants. Nevertheless, further detailed analysis is needed to explore the functional relationships between these proteins and αKNL2.

E3 ubiquitin ligases play a significant role in substrate degradation during the cell cycle, with APC/C and Cullins being major classes involved in protein degradation and cell cycle progression (Frescas and Pagano 2008; Pesin and Orr-Weaver 2008; Mocciaro and Rape 2012; Genschik et al. 2014; Willems and De Veylder 2022). During mitosis, the APC/C promotes the separation of sister chromatids and the transition out of mitosis by degrading mitotic cyclins and Securin (Zachariae and Nasmyth 1999; Pesin and Orr-Weaver 2008). Although its activity is generally more prominent during the transition from metaphase to anaphase, APC/C can degrade certain proteins such as cyclin A (Fry and Yamano 2006) and NIMA-related kinase 2A (Nek2A) (Singh et al. 2014) at the onset of mitosis. We have demonstrated that αKNL2 directly interacts with the APC/C component APC10 and its activator CDC20.1 similarly to several mitotic regulators, including cyclin A, B1, and Nek2A (Hayes et al. 2006; Guo et al. 2016). Moreover, we showed that αKNL2 levels in planta were increased upon application of the APC/C inhibitor Apcin, which competitively inhibits APC/C-dependent ubiquitination by binding to CDC20 (Sackton et al. 2014; Richeson et al. 2020).

During the mitotic cell cycle, αKNL2 is present during interphase but disappears at the early stages of mitosis (Lermontova et al. 2013). Our findings indicate that αKNL2 localizes to centromeres during the prometaphase to anaphase stages in *APC10-RNAi* lines, suggesting that its degradation occurs at the onset of mitosis, similar to cyclin A. We suggest that cell cycle-dependent degradation of αKNL2 occurs through APC/C and is crucial for proper mitotic progression. Interestingly, previous studies in humans have shown that MIS18BP1/KNL2 levels decrease on metaphase centromeres and rise in late anaphase/telophase (Jansen et al. 2007; Erhardt et al. 2008), aligning with CENP-A loading. In Arabidopsis, we propose that αKNL2 is needed for CENH3 loading during the late G2 phase of the cell cycle and is subsequently degraded at the onset of mitosis through APC/C-mediated proteolysis (Fig. 8).

CDC20 can bind substrates with destruction boxes (D-boxes), facilitating polyubiquitination of the substrate. Notably, the APC10 subunit also directly contributes toward D-box recognition of substrates (Carroll et al. 2005; da Fonseca et al. 2011). Our data show that αKNL2 is a direct target of the APC/C-CDC20 complex, with the D-box1 of αKNL2 playing a critical role in its recognition and degradation. The mutation or deletion of this region leads to an accumulation of αKNL2, resulting in severe mitotic defects, as evidenced by increased rates of misaligned metaphases and anaphase bridges in root tip meristems in Arabidopsis. Moreover, the deletion of the SBC domain, recognized for binding Cullins, confirmed that Cullin E3 ligases are not responsible for αKNL2 degradation. APC/C complex degrades several substrates which is critical for metaphase to anaphase transition (Genschik et al. 1998; Hayes et al. 2006; Guo et al. 2016). APC/C-specific degrons, such as D-boxes, KEN, and ABBA motifs, have been identified in the KNL2/M18BP1 protein across various species (Supplementary Table S3). Additionally, our data suggested interactions between *Ce*KNL-2 and the APC/C components MAT-1, EMB27, MAT-3, and FZY-1 in *C. elegans* through the KEN and ABBA motifs. These findings suggest that APC/C-mediated degradation of KNL2 may be a conserved mechanism critical for proper cell division. However, the mechanism of KNL2 degradation must be studied in individual organisms to understand its specific role in centromere regulation.

Although E2 conjugating enzymes were identified in the αKNL2 interactome and BiFC analysis, the Y2H analysis did not confirm their direct interaction with αKNL2. This is consistent with the fact that E2 enzymes interact via E3 ligases rather than directly with substrates. In yeast, the UbcH10 E2 enzyme (homolog of UBC19) was proposed to regulate the selection of APC/C substrates. Nonetheless, even with more than a 90% reduction in UbcH10 levels, the processes of mitosis and cyclin degradation are not disrupted (Summers et al. 2008; Walker et al. 2008). Therefore, the BiFC interaction of UBC19/20 with αKNL2 may represent a weak or transient association during the ubiquitination process in plant cells. Ubiquitination occurs via lysine (Lys) residues of the substrate where the ubiquitin protein binds (Sadanandom et al. 2012). We identified lysine residues K336 and K339 as critical sites for αKNL2 polyubiquitination in both in vitro and in vivo. αKNL2^Mut-UBI2^ showed more specific inhibition of αKNL2 polyubiquitination due to mutations of both Lys336 and Lys339. Similarly, in humans, polyubiquitination sites Lys49 and Lys124 are crucial for proper phosphorylation-dependent degradation of CENPA, and mutations lead to mislocalization of CENP-A (Wang et al. 2021).

Expression of constructs containing αKNL2 mutated at ubiquitination sites or with deleted D-box1 in Arabidopsis abolished its degradation and resulted in protein overaccumulation. Consequently, it resulted in reduced root and plant growth, mitotic abnormalities, and seed abortion. Similar effects were observed when αKNL2 was knocked out (Lermontova et al. 2013). Depletion of KNL2 in humans, mice, or *C. elegans* results in mitotic defects (Maddox et al. 2007; Kim et al. 2012), likely due to impaired function of SAC components. Studies in *C. elegans* lacking KNL2 showed improper localization of the BUB-1 protein to chromosomes (Maddox et al. 2007). In line with the data, the overaccumulation of αKNL2 due to disrupted selective degradation led to increased expression of *CENH3* and SAC genes (prolonged SAC activation), which may contribute to mitotic cell cycle defects. Thus, it is crucial to maintain appropriate levels of αKNL2, avoiding both its reduction and overaccumulation. This highlights the importance of critical balance between protein synthesis and degradation in cellular homeostasis.

Our study opens several avenues for future research and one area of interest is investigating the role of other posttranslational modifications in regulating αKNL2 stability could provide a more comprehensive understanding of its regulation. Additionally, Ahmadli et al. (2023) reported that the *αknl2* mutant can serve as a haploid inducer when pollinated by the wild type, with temperature stress enhancing its haploid induction efficiency 10-fold. Therefore, examining the impact of degradation-resistant αKNL2 lines on haploid induction could have significant potential for crop improvement and agricultural applications. In summary, our results provide insights into the regulatory mechanisms controlling KNL2 stability via APC/C^CDC20^-mediated degradation. This regulation is conserved in *A. thaliana* and *C. elegans*, and it is essential for proper chromosome segregation, emphasizing the critical role of targeted protein degradation in cellular function. Future studies should aim to expand our understanding of these conserved processes, with potential applications in plant breeding and medicine.

## Materials and methods

### Plasmid construction

The entire ORFs of *UBC19*, *UBC20*, *APC2*, *CUL1*, *CUL3*, *APC10*, *CDH1.1*, and *CDC20.1* fragments were amplified by RT-PCR using RNA isolated from flower buds of Arabidopsis (*A. thaliana*) wild type with primers listed in Supplementary Table S4. Obtained fragments were cloned into the pDONR221 backbone via the Gateway BP reaction (Invitrogen). The αKNL2, αKNL2-N, and αKNL2-C clones in the pDONR221 vector were generated previously (Lermontova et al. 2013).

From the pDONR221 clones, the resulting fragments were recombined into Gateway-compatible destination vectors for downstream applications: pGWB641 (http://shimane-u.org/nakagawa/gbv.htm) was used for in vivo subcellular localization studies; pGBKT7 and pGADT7 vectors were used for Y2H screening and cotransformation; 3′Venus-N-pBAR GW and 3′Venus-C-pBAR GW contain cMYC and HA tags for BiFC and Co-IP analysis (Yadala et al. 2024). For affinity purification, the coding sequences of the αKNL2-N and αKNL2-C fragments were amplified from pDONR221 entry clones using primer pairs with the added SalI and BamHI restriction sites (Supplementary Table S4) and cloned into the SalI/BamHI sites of the pCambia 2300 vector containing the C-terminal GS tag.

αKNL2 full-length fragments mutated simultaneously at Mut-UBI1 and Mut-UBI2 ubiquitination sites were synthesized and provided as clones in Gateway-compatible pENTR-TOPO vector by Twist Bioscience (https://www.twistbioscience.com/). Domain deletion/site-specific mutant variants of αKNL2 were generated in pDONR221 vector carrying full-length αKNL2 using a site-directed mutagenesis protocol (Phusion Site-Directed Mutagenesis Kit, Thermo Scientific). Mutagenized αKNL2 fragments were recombined from pDONR221 or pENTR-TOPO clones into the Gateway-compatible pGWB641 vector via the Gateway LR reaction (Invitrogen).

### Plant transformation and cultivation

The transient transformation of *N. benthamiana* plants with *Agrobacterium* (*A. tumefaciens*) was performed according to the method outlined by Walter et al. (2004). Fluorescence was assessed in the lower epidermal cell layers of *N. benthamiana* leaves 2 d after infiltration, with each expression plasmid being transformed into *N. benthamiana* in at least 3 independent infiltration experiments.

Plasmids used for the transformation of Arabidopsis were transferred into the *A. tumefaciens* strain GV3101 by electroporation. Plants of *A. thaliana* accession Columbia-0 (Col-0) ecotype were transformed according to the flower dip method (Clough and Bent 1998). T1 transformants were selected on ½ Murashige and Skoog medium (Murashige and Skoog 1962) containing 50 mg/L phosphinothricin or 50 mg/L of kanamycin and 50 mg/L hygromycin. After obtaining transgenic lines, the plant morphology and GFP signals, resulting from the expression of each plasmid, were examined by using at least 3 independent single-insertion plant lines.

*A. thaliana* and *N. benthamiana* plants, used for localization studies, BiFC analysis, and nuclei extraction, were grown under specific temperature conditions*. A. thaliana* was grown under 16 and 8 h photoperiod with a day/night temperature regime of 20/18 °C, with 100 *μ*mol m^−2^ s^−1^ light intensity, whereas *N. benthamiana* plants were grown under 12 h photoperiod at a constant temperature of 26 °C.

### Nematode maintenance and strain generation

Nematode (*C. elegans*) strains were maintained using standard conditions at 20 °C unless otherwise noted. N2 was used as a wild-type strain, and all of the genetic modifications were performed in this background. A C-terminal double-HA tag was inserted at the *knl-2* endogen using CRISPR/Cas-9 technology as described in Arribere et al. (2014) to create strain FAS111 *knl-2*(uge70[*knl-2*::2xHA]).

### Affinity purification-mass spectrometry

Arabidopsis suspension-cultured PSB-D cells were maintained and transformed using recombinant Agrobacteria essentially as previously described (Van Leene et al. 2015). Protein isolation and purification were essentially performed as previously described (Antosz et al. 2017; Michl-Holzinger et al. 2022). In brief, after sonification of 15 g cells in extraction buffer (25 mm HEPES-KOH pH 7.4, 0.05% IGEPAL CA-630, 1 mm DTT, 100 mm NaCl, 2 mm MgCl_2_, 5 mm EGTA, 10% glycerol, proteinase inhibitor cocktail, 1 mm PMSF), the unspecific endonuclease Benzonase (50 μ/mL extract) was added to degrade DNA and RNA, facilitating the detection of protein interactions. GS-tagged proteins were affinity-purified from Benzonase-treated protein extracts using IgG-coupled magnetic beads and eluted proteins were analyzed by SDS-PAGE and digested with trypsin. Mass spectrometry was performed as previously described (Antosz et al. 2017). In brief, proteins were separated on a NuPAGE 4% to 12% Bis-Tris gel (Invitrogen), and gel lanes were then cut into slices; washed with 50 mm NH_4_HCO_3_, 50 mm NH_4_HCO_3_/acetonitrile (3/1), and 50 mm NH_4_HCO_3_/acetonitrile (1/1); and lyophilized. After a reduction/alkylation step with DTT and iodoacetamide, respectively, washing steps and lyophilization were repeated. Proteins were subjected to in-gel digest with trypsin (Trypsin Gold, mass spectrometry grade, Promega) overnight at 37 °C. Peptides were extracted twice with 100 mm NH_4_HCO_3_ followed by an elution step with 50 mm NH_4_HCO_3_ in 50% acetonitrile.

After lyophilization, peptides were reconstituted in 1% formic acid prior to LC-MS/MS analysis. Peptide separation was carried out by an UltiMate 3000 RSLCnano System (Thermo Fisher Scientific) equipped with a C18 Acclaim Pepman100 preconcentration column (100 *µ*m i.d. ×20 mm, Thermo Fisher Scientific). A linear gradient of 4% to 40% acetonitrile in 0.1% formic acid over 90 min at a flow rate of 300 nL/min was used. The LC-system was on-line coupled to a maXis plus UHR-QTOF system (Bruker Daltonics) via a CaptiveSpray nanoflow electrospray source (Bruker Daltonics). Data-dependent acquisition of MS/MS spectra after collision-induced dissociation fragmentation was performed at a minimum resolution of 60,000. For the precursor scan, the MS spectra rate was 2 Hz processing a mass range between m/z 175 and m/z 2000. A dynamic method with a fixed cycle time of 3 s was applied via the Compass 1.7 acquisition and processing software (Bruker Daltonics). Raw data processing was performed in Data Analysis 4.2 (Bruker Daltonics) and Protein Scape 3.1.3 (Bruker Daltonics) in connection with Mascot 2.5.1 (Matrix Science) was used to search against the TAIR10 database. Search parameters were as follows: enzyme specificity trypsin with 1 missed cleavage allowed precursor tolerance 0.02 Da, MS/MS tolerance 0.04 Da, acetylation of protein N-term, carbamidomethylation or propionamide modification of cysteine, oxidation of methionine, deamidation of asparagine, and glutamine were set as variable modifications. The mascot peptide ion-score cutoff was set at 15. Protein list compilation was done using the Protein Extractor function of Protein Scape. A MASCOT score of a minimum of 60 and the presence of the candidate protein in at least 2 purifications were considered criteria for reliable identification of the protein as a component of the αKNL2 interaction network. The experimental background (proteins copurified with the unfused GS tag) and nonspecific interactions (proteins known to be copurified regardless of the bait used) were considered false-positive hits and excluded from further analysis.

### Y2H library screening

The cDNAs encoding αKNL2 full-length protein or its N-terminal (aa 1 to 363) and C-terminal (364 to 599) fragments were cloned into the pGBKT7 vector as baits. The pGBKT7 vectors were transformed by electroporation into the yeast (*Saccharomyces cerevisiae*) AH109 strain following the manufacturer's instructions of the MatchMaker Gold Yeast Two-Hybrid System and used to screen the 2-hybrid cDNA library (Mate & Plate Library—Universal Arabidopsis [Normalized], from Clontech and TaKaRa; https://www.takarabio.com).

αKNL2 binding proteins were identified by growth on high stringency quadruple dropout medium (-Leu/-Trp/-His/-Ade) after incubation for 3 to 5 d at 30 °C. More than 1 million diploids were screened for each of the 3 bait constructs. Positive colonies were transferred to a fresh selection medium, and the cDNA present in the pGADT7 prey vector was determined after colony PCR and Sanger sequencing. After inspection of the clones, we selected only in-frame fusions corresponding to 51, 85, and 173 positive clones for the αKNL2, αKNL2-N, and αKNL2-C constructs that correspond to 38, 54, and 76 different proteins, respectively.

### IP-MS of KNL2 from *C. elegans*

Synchronized young gravid adult worms were washed 3 times with M9, and embryos were obtained by hypochlorite treatment. Embryos were resuspended in lysis buffer (50 mm Tris-HCl [pH 7.4], 500 mm NaCl, 0.25% deoxycholate, 10% glycerol, 1% NP-40, 2 mm DTT, 1× EDTA-free protease inhibitor cocktail [Roche, Switzerland], 1×PhosSTOP [Roche]) and frozen in liquid nitrogen. Samples were sonicated with a Bioruptor Pico (Diagenode, Belgium—15 cycles, 30-s sonication, 30-s rest, with snap freezing every 5 cycles) and spun down (30 min, max speed) to pellet debris. The supernatant was collected, and prewashed Pierce Anti-HA Magnetic Beads (Thermo Fisher Scientific, USA) were added. Following overnight incubation on a rotator at 4 °C, the beads were collected using a magnetic stand and washed according to manufacturer's instructions. Beads were then boiled in Pierce Lane Marker Non-Reducing Sample Buffer (Thermo Fisher Scientific). The eluates were analyzed by the Proteomics Facility at the Functional Genomics Center Zurich, Switzerland. Samples were processed according to standard procedures used by the facility. The proteins were precipitated with trichloroacetic acid, washed with acetone, resuspended, and digested with trypsin. Samples were then dried and dissolved in 0.1% formic acid, and ∼10% of the sample was injected into the liquid chromatography and tandem mass spectrometry (LC-MS/MS) system.

### BiFC assay

For visualization of protein interactions in vivo, BiFC was performed as described (Yadala et al. 2022). Leaves of 2- to 4-week-old *N. benthamiana* plants were coinfiltrated with the *A. tumefaciens* strain GV3101 carrying 2 BiFC expression vectors encoding the proteins of interest. The HC-Pro, a suppressor of RNA silencing, was used. Each BiFC combination was tested in at least 3 independent infiltrations using 3 different plants (*n* = 3, biological replicates).

### Y2H cotransformation

To check the interaction between 2 selected proteins, pGADT7 and pGBKT7 vectors carrying cDNA encoding the proteins of interest, were cotransformed into *S. cerevisiae* Y2H Gold strain following Matchmaker Gold Y2H System protocol (Takara Bio, Cat #630489). The transformed cells were grown on –LT agar plates for 3 to 5 d at 30 °C. Picked colonies were then serially diluted (1/10, 1/100, 1/1000) and dropped in parallel onto –LT (SD/-Leu/-Try), and –LTH (SD/-Leu/-Try/-His) selective media from which the positive interactions were scored. Cotransformations and interaction validations were performed in at least 2 independent experiments.

### Chemical treatments and in vivo protein degradation

For chemical treatments, 26S proteasome inhibitors, MG115 (MedChemExpress) and bortezomib (Selleckchem), and translation inhibitor, CHX (Selleckchem), were dissolved in DMSO and used at a concentration of 100 *μ*M. An APC/C inhibitor, Apcin (MedChemExpress), was used in different concentrations such as 5, 10, 15, 25, and 50 *µ*M.

For analysis of αKNL2 degradation by the 26S proteasome, a plasmid carrying αKNL2-EYFP was infiltrated into the *N. benthamiana* plants and treated with the indicated concentrations of CHX, MG115, bortezomib, Apcin, or DMSO (control). Similarly, 1-week-old transgenic Arabidopsis seedlings transformed with αKNL2-EYFP were incubated in liquid Murashige and Skoog medium (Duchefa Biochemie) with Apcin or DMSO for 16 h. EYFP fluorescence was detected by microscopy.

### Protein extraction, immunoprecipitation, and immunoblot analyses

Total protein extracts were isolated from *N. benthamiana* leaves infiltrated with constructs expressing αKNL2-EYFP treated with MG115, bortezomib, Apcin, or αKNL2 variants mutagenized at D-box or ubiquitin sites using phenol extraction method as described previously (Hurkman and Tanaka 1986). The nuclear protein extracts were isolated from wild type and *APC10-RNAi* according to Huang et al. (2021). Protein extracts were isolated in triplicate for each genotype or treatment across 3 independent experiments. IP and Co-IP were conducted according to the manufacturer's instructions using a GFP- and HA-trap kit (Chromotek, https://www.chromotek.com). The samples were further used for immunoblotting analyses.

For IB analyses, SDS sample buffer (2×) was added to each protein sample and boiled for 10 min. The protein samples were separated by SDS-PAGE in a 10% acrylamide gel and electro-transferred to a PVDF transfer membrane (Thermo Scientific). The membranes were blocked with 5% (w/v) low-fat milk powder dissolved in phosphate-buffered saline (PBS) for 1 h at room temperature. The membranes were incubated with a mouse monoclonal anti-ubiquitin (1:2000, Santa Cruz, #P4D1), mouse anti-GFP (1:1000, JL8, Living Colors), mouse anti-HA (1:10000), mouse anti-cMYC (1:500), rabbit anti-αKNL2 (1:1000), or mouse anti-tubulin (1:1000, Sigma, #T9026) antibodies for 12 h at 4 °C in 1% BSA/PBS. Bound antibodies were detected by incubation with anti-mouse or anti-rabbit antibodies (1:5000) conjugated to IRDye 800CW and visualized using an LI-COR Odyssey scanner. The signal intensities were quantified with the Image Studio software (Version 3.1, LI-COR Biosciences).

### RNA isolation and RT-qPCR analysis

Total RNA was isolated from 7-day-old seedlings of both wild-type and degradation-resistant αKNL2 plants using the TRIzol reagent. The extracted RNA was treated with DNase, and the first-strand cDNA synthesis was performed using a Genaxxon Scriptase RT cDNA synthesis kit with an oligo(dT)18 primer and 2 *µ*g of total RNA as the template. Quantitative real-time RT-PCR was conducted on an Applied Biosystems QuantStudio 6 Flex Real-Time PCR System using SYBR Green Supermix (Genaxxon). Each transcript was quantified in triplicate across 3 independent biological replicates. ACTIN and UBQ cDNA were amplified for normalization. PCR reactions (10 *μ*L) were set up using cDNA to amplify *ACTIN*, *UBQ*, *KNL2*, *CENH3*, *MAD2*, *BUBR1*, and *BUB3.1*. The thermal cycling conditions for all transcripts consisted of an initial denaturation at 95 °C for 5 min, followed by 40 cycles of 15 s denaturation at 95 °C, 30 s annealing at 62 °C, and 30 s elongation at 72 °C.

### Immunostaining

The samples were prepared from *N. benthamiana* leaves infiltrated by a plasmid carrying a gene encoding with protein of interest tagged to EYFP. The extraction of nuclei from the infiltrated leaves was performed as described previously (Doležel et al. 2007). With the sample preparation described above, the protein expression with EYFP fluorescence remains intact, and colocalization analysis was performed by immunostaining with *N. benthamiana*–specific CENH3. In *A. thaliana*, the nuclei/chromosome preparations for mitotic analysis were prepared as described (Lermontova et al. 2006). Immunostaining of nuclei/chromosomes was performed as described (Jasencakova et al. 2000). Primary antibodies used included rabbit *N. benthamiana*–specific CENH3 (1:1000), rabbit anti-αKNL2 (1:1000), mouse anti-GFP (1:1000), and mouse anti-tubulin (1:1000), along with an anti-rabbit rhodamine-conjugated (1:300) and anti-mouse FITC secondary antibody (1:300). 4′,6-Diamidino-2-phenylindole (DAPI) (Vector Laboratories, USA) was used as a counterstain.

### Microscopy analysis of fluorescent signals

Transformed *N. benthamiana* leaves and *A. thaliana* seedlings were used for EYFP fluorescence detection with a confocal LSM 780 laser scanning microscope (Carl Zeiss GmbH), employing a 40 × NA 1.2 water objective. EYFP was excited with a 488-nm laser line and the specific fluorescence recorded with a 505- to 550-nm band-pass filter in combination with a 37 *µ*m pinhole, pixel dwell time 1.58 *µ*s and 0,104 m scaling. Images were recorded as Z-stacks with ensuing maximum intensity projection. To examine the colocalization of CENH3 and αKNL2-EYFP signals in *N. benthamiana* nuclei at a resolution of 120 nm (superresolution achieved with a 488 nm laser excitation), spatial structured illumination microscopy (3D-SIM) was performed with a 63×/1.4 Oil Plan-Apochromat objective of an Elyra PS.1 microscope system and the software ZENBlack (Carl Zeiss GmbH). Images were captured separately for each fluorochrome using the 561 (100 mW), 488 (100 mW), and 405 (50 mW) nm laser lines for excitation and appropriate emission filters (Weisshart et al. 2016; Kubalová et al. 2021). The SIM image stack raw data were acquired with the following settings: 13% power of all lasers; Andor EM-CCD camera iXon DU 885 gain 30; and exposure time 100 ms.

### Seed set analysis and evaluation of plant fertility

For the seed set analysis, siliques were fixed in ethanol-acetic acid solution (9:1) overnight, followed by dehydration in 70% and 90% ethanol for 1 h each. The samples were cleared in chloral hydrate solution (chloral hydrate: water: glycerol = 8:2:1) overnight at 4 °C. Seed counts within the siliques were conducted under a binocular microscope (Carl Zeiss GmbH, Germany). Scanning electron microscopy was performed to assess plant fertility in αKNL2 degradation-resistant lines. Freshly isolated siliques were fixated with 4% formaldehyde in 50 mm phosphate buffer pH 7.0 for 16 h at 8 °C. After brief washing with distilled water, samples were dehydrated in an ascending ethanol series of 30%, 50% 70%, 90%, and 100% 2 times each, followed by critical point drying in a Quorum K850 critical point dryer (Quorum Technologies Ltd.; https://www.quorumtech.com). Dried samples were placed onto carbon adhesive discs, gold coated in an Edwards S150B sputter coater (Edwards High Vacuum Inc.; http://www.edwardsvacuum.com), and examined in a Zeiss Gemini300 scanning electron microscope (Carl Zeiss Microscopy GmbH; https://www.zeiss.de) at 5 kV acceleration voltage. Images were stored as TIFF files.

### Bioinformatic analysis

VENNY 2.1 tool (https://bioinfogp.cnb.csic.es/tools/venny/) was used for comparing lists of common αKNL2 interactors identified by Y2H and AP-MS screening. Cytoscape v3.8.2 software (https://cytoscape.org/) was used for visualization and analysis of interaction networks. Metascape (http://metascape.org/) web tool was used for GO and network analysis. Database for Annotation, Visualization, and Integrated Discovery (DAVID), a functional annotation tool (https://davidbioinformatics.nih.gov/), was used to analyze the orthologs in Arabidopsis. AlphaFold 3 (https://alphafoldserver.com/) was employed for structural predictions. The input sequences were retrieved from UniProt, and the template and MSA search settings were configured according to the parameters described in the Abramson et al. (2024). GPS-ARM (https://arm.biocuckoo.org/) and Eukaryotic Linear Motif (ELM; http://elm.eu.org/) web sources were used to identify the degrons present in αKNL2.

### Quantification and statistical analysis

The primary root lengths and intensity of immunosignals were measured using the ImageJ software. The mean ± SEM from a minimum of 3 separate experiments or samples was represented by error bars. For pairwise comparisons, Welch's *t*-test was used to account for unequal variances and was performed using Excel's built-in T.TEST function (2-tailed, unequal variance). For comparisons involving more than 2 groups, 1-way ANOVA followed by Tukey's post hoc multiple comparison test was conducted using GraphPad Prism software. Statistical significance was defined as follows: the *P* < 0.5 was taken to be significant (*), and *P* < 0.05 was deemed very significant (**), while *P* < 0.005 was deemed highly significant (***) for all performed tests. All statistical outputs are provided in Supplementary Data Set 2.

### Accession numbers

Sequence data from this article can be found in the GenBank/EMBL data libraries under the following accession numbers: AT5G02520 (*αKNL2*), AT1G01370 (*CENH3*), AT3G20060 (*UBC19*), AT1G50490 (*UBC20*), AT2G04660 (*APC2*), AT4G02570 (*CUL1*), AT1G26830 (*CUL3*), AT2G18290 (*APC10*), AT4G22910 (*CDH1.1*), AT4G33270 (*CDC20.1*), AT3G25980 (*MAD2*), AT2G33560 (*BUBR1*), AT3G19590 (*BUB3.1*), AT3G01280 *(VDAC1*), AT1G16190 (*RAD23A*), AT5G38470 (*RAD23D*), AT4G39960 (*DJA5*), AT2G22360 (*DJA6*), and AT5G61790 (*CNX1*).

## Supplementary Material

koaf164_Supplementary_Data

## Data Availability

The mass spectrometry proteomics data have been deposited to the ProteomeXchange Consortium via the PRIDE partner repository with the data set identifier PXD051741 and 10.6019/PXD051741.

## Supplementary data

The following materials are available in the online version of this article.

**Supplementary Figure S1.** KNL2 interactors detected by both Y2H screening and AP-MS analysis in Arabidopsis.

**Supplementary Figure S2.** Gene annotation enrichment analysis of KNL2 interaction partners identified by Y2H and AP-MS in Arabidopsis and by IP-MS in *C. elegans*.

**Supplementary Figure S3.** The protein–protein interaction networks involving αKNL2 interactors and their functional annotation in Arabidopsis.

**Supplementary Figure S4.** The protein–protein interaction networks involving αKNL2 interactors and their functional annotation in *C. elegans*.

**Supplementary Figure S5.** BiFC interaction analysis of selected candidates with αKNL2 and quantitative analysis of BiFC assay.

**Supplementary Figure S6.** Y2H assay showing interactions between αKNL2 (bait) and screened E2 and E3 enzymes.

**Supplementary Figure S7.** AlphaFold 3 interaction of *Ce*KNL-2 with MAT-1 or EMB-27.

**Supplementary Figure S8.** Analysis of *APC10*, *αKNL2*, and *CENH3* levels in *APC10-RNAi* lines.

**Supplementary Figure S9.** The localization of αKNL2 during mitosis in cells of *APC10-RNAi* lines.

**Supplementary Figure S10.** D-box2 and SBC were not the active degrons in αKNL2.

**Supplementary Figure S11.** Apcin inhibits the interaction of αKNL2 and APC/C complex.

**Supplementary Figure S12.** The deletion of D-box1 and treatment with Apcin affects the αKNL2 interaction with ubiquitin conjugates.

**Supplementary Figure S13.** 336 and 339 K of αKNL2 are the targets for binding ubiquitin protein.

**Supplementary Figure S14.** The phenotype characteristics of degradation-resistant plants of αKNL2 in Arabidopsis.

**Supplementary Table S1.** BiFC interaction analysis of selected candidates with αKNL2.

**Supplementary Table S2.** Y2H interaction analysis of selected candidates with αKNL2.

**Supplementary Table S3.** APC/C-specific degrons present in KNL2 protein across species.

**Supplementary Table S4.** Primers used in this study.

**Supplementary Data Set 1.** Proteins identified by IP-MS of KNL-2 in *C. elegans*.

**Supplementary Data Set 2.** Summary of statistical analysis.

## Dive Curated Terms

The following phenotypic, genotypic, and functional terms are of significance to the work described in this paper:

APC2 Gramene: AT2G04660
APC2 Araport: AT2G04660

UBC19 Gramene: AT3G20060
UBC19 Araport: AT3G20060

UBC20 Gramene: AT1G50490
UBC20 Araport: AT1G50490

VDAC1 Gramene: AT3G01280
VDAC1 Araport: AT3G01280

KNL2 Gramene: AT5G02520
KNL2 Araport: AT5G02520

CUL1 Gramene: AT4G02570
CUL1 Araport: AT4G02570

MAD2 Gramene: AT3G25980
MAD2 Araport: AT3G25980

CDC20.1 Gramene: AT4G33270
CDC20.1 Araport: AT4G33270

BUBR1 Gramene: AT2G33560
BUBR1 Araport: AT2G33560

BUB3.1 Gramene: AT3G19590
BUB3.1 Araport: AT3G19590

CDH1.1 Gramene: AT4G22910
CDH1.1 Araport: AT4G22910

CUL3 Gramene: AT1G26830
CUL3 Araport: AT1G26830

APC10 Gramene: AT2G18290
APC10 Araport: AT2G18290

RAD23A Gramene: AT1G16190
RAD23A Araport: AT1G16190

RAD23D Gramene: AT5G38470
RAD23D Araport: AT5G38470

DJA5 Gramene: AT4G39960
DJA5 Araport: AT4G39960

DJA6 Gramene: AT2G22360
DJA6 Araport: AT2G22360

CNX1 Gramene: AT5G61790
CNX1 Araport: AT5G61790
