# Supplementary material for: Ubiquitin-dependent proteolysis of KNL2 driven by APC/CCDC20 is critical for centromere integrity and mitotic fidelity
Source: Plant Cell. 2025 Jun 25;37(7):koaf164. doi: 10.1093/plcell/koaf164 (PMC12231567; doi:10.1093/plcell/koaf164)
Supplement: koaf164_Supplementary_Data [file koaf164_supplementary_data.zip › Supplementary Data.pdf]

A

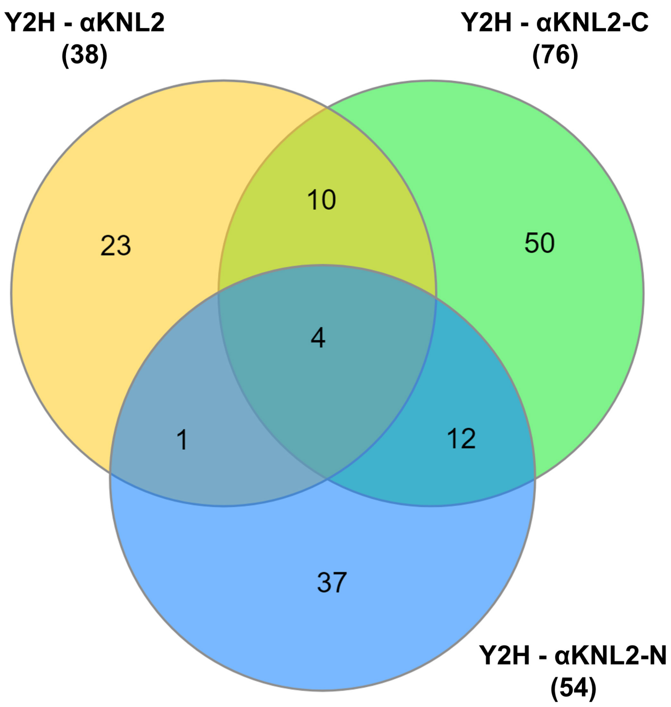

B

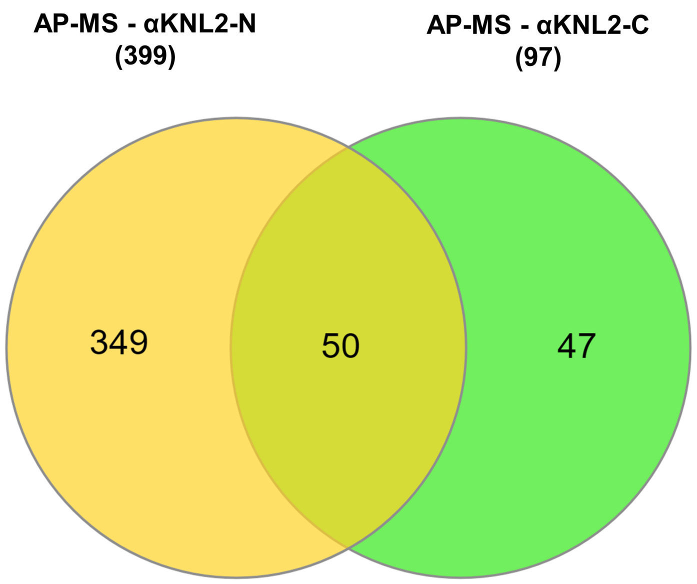

C

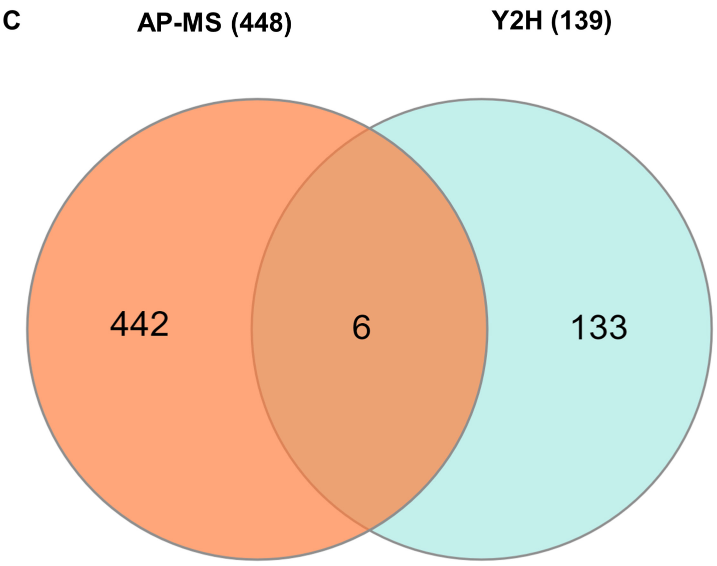

D

ID	Name
AT3G01280	Voltage-dependent anion-selective channel 1 (VDAC1)
AT5G61790	Calnexin homolog 1 (CNX1)
AT2G22360	DnaJ protein (DJA6)
AT4G39960	DnaJ protein (DJA5)
AT1G16190	Ubiquitin receptor (RAD23A)
AT5G38470	Ubiquitin receptor (RAD23D)

Supplementary Figure S1. KNL2 interactors detected by both Y2H screening and AP-MS analysis in Arabidopsis (Supports Figure 2)

(A-C) Venn plots showing the number of common interactors for αKNL2, αKNL2-N, and αKNL2-C fragments revealed by **(A)** Y2H screening, **(B)** AP-MS analysis, **(C)** both AP-MS and Y2H techniques. **(D)** The list of six αKNL2 interactors identified by both AP-MS and Y2H. Y2H - Yeast-two hybrid; AP-MS - Affinity purification-mass spectrometry.

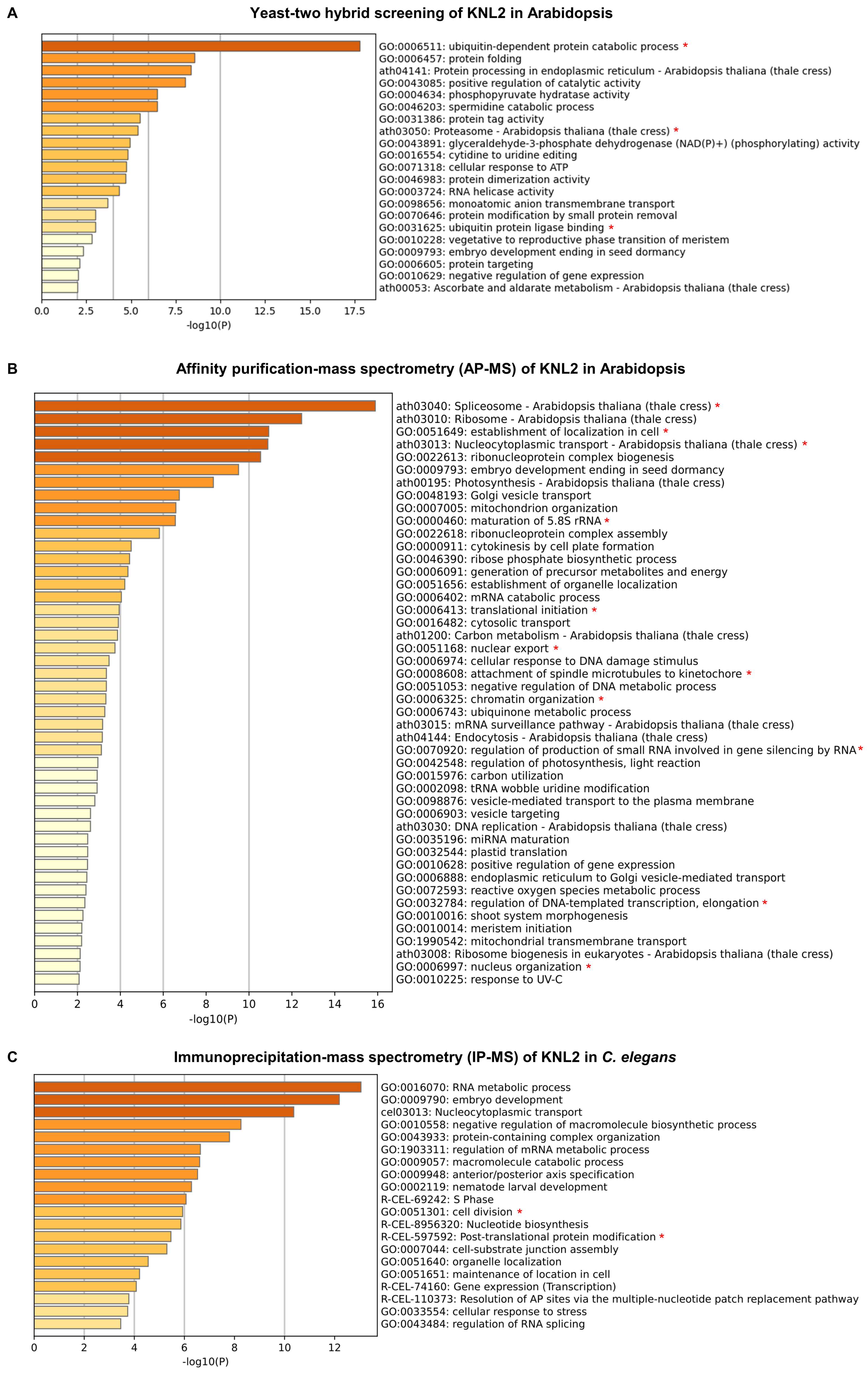
A
Yeast-two hybrid screening of KNL2 in Arabidopsis
GO:0006511: ubiquitin-dependent protein catabolic process *
GO:0006457: protein folding
ath04141: Protein processing in endoplasmic reticulum - Arabidopsis thaliana (thale cress)
GO:0043085: positive regulation of catalytic activity
GO:0004634: phosphopyruvate hydratase activity
GO:0046203: spermidine catabolic process
GO:0031386: protein tag activity
ath03050: Proteasome - Arabidopsis thaliana (thale cress) *
GO:0043891: glyceraldehyde-3-phosphate dehydrogenase (NAD(P)+) (phosphorylating) activity
GO:0016554: cytidine to uridine editing
GO:0071318: cellular response to ATP
GO:0046983: protein dimerization activity
GO:0003724: RNA helicase activity
GO:0098656: monoatomic anion transmembrane transport
GO:0070646: protein modification by small protein removal
GO:0031625: ubiquitin protein ligase binding *
GO:0010228: vegetative to reproductive phase transition of meristem
GO:0009793: embryo development ending in seed dormancy
GO:0006605: protein targeting
GO:0010629: negative regulation of gene expression
ath00053: Ascorbate and aldarate metabolism - Arabidopsis thaliana (thale cress)
0.0 2.5 5.0 7.5 10.0 12.5 15.0 17.5
-log10(P)
B
Affinity purification-mass spectrometry (AP-MS) of KNL2 in Arabidopsis
ath03040: Spliceosome - Arabidopsis thaliana (thale cress) *
ath03010: Ribosome - Arabidopsis thaliana (thale cress)
GO:0051649: establishment of localization in cell *
ath03013: Nucleocytoplasmic transport - Arabidopsis thaliana (thale cress) *
GO:0022613: ribonucleoprotein complex biogenesis
GO:0009793: embryo development ending in seed dormancy
ath00195: Photosynthesis - Arabidopsis thaliana (thale cress)
GO:0048193: Golgi vesicle transport
GO:0007005: mitochondrion organization
GO:0000460: maturation of 5.8S rRNA *
GO:0022618: ribonucleoprotein complex assembly
GO:0000911: cytokinesis by cell plate formation
GO:0046390: ribose phosphate biosynthetic process
GO:0006091: generation of precursor metabolites and energy
GO:0051656: establishment of organelle localization
GO:0006402: mRNA catabolic process
GO:0006413: translational initiation *
GO:0016482: cytosolic transport
ath01200: Carbon metabolism - Arabidopsis thaliana (thale cress)
GO:0051168: nuclear export *
GO:0006974: cellular response to DNA damage stimulus
GO:0008608: attachment of spindle microtubules to kinetochore *
GO:0051053: negative regulation of DNA metabolic process
GO:0006325: chromatin organization *
GO:0006743: ubiquinone metabolic process
ath03015: mRNA surveillance pathway - Arabidopsis thaliana (thale cress)
ath04144: Endocytosis - Arabidopsis thaliana (thale cress)
GO:0070920: regulation of production of small RNA involved in gene silencing by RNA *
GO:0042548: regulation of photosynthesis, light reaction
GO:0015976: carbon utilization
GO:0002098: tRNA wobble uridine modification
GO:0098876: vesicle-mediated transport to the plasma membrane
GO:0006903: vesicle targeting
ath03030: DNA replication - Arabidopsis thaliana (thale cress)
GO:0035196: miRNA maturation
GO:0032544: plastid translation
GO:0010628: positive regulation of gene expression
GO:0006888: endoplasmic reticulum to Golgi vesicle-mediated transport
GO:0072593: reactive oxygen species metabolic process
GO:0032784: regulation of DNA-templated transcription, elongation *
GO:0010016: shoot system morphogenesis
GO:0010014: meristem initiation
GO:1990542: mitochondrial transmembrane transport
ath03008: Ribosome biogenesis in eukaryotes - Arabidopsis thaliana (thale cress)
GO:0006997: nucleus organization *
GO:0010225: response to UV-C
0 2 4 6 8 10 12 14 16
-log10(P)
C
Immunoprecipitation-mass spectrometry (IP-MS) of KNL2 in C. elegans
GO:0016070: RNA metabolic process
GO:0009790: embryo development
cel03013: Nucleocytoplasmic transport
GO:0010558: negative regulation of macromolecule biosynthetic process
GO:0043933: protein-containing complex organization
GO:1903311: regulation of mRNA metabolic process
GO:0009057: macromolecule catabolic process
GO:0009948: anterior/posterior axis specification
GO:0002119: nematode larval development
R-CEL-69242: S Phase
GO:0051301: cell division *
R-CEL-8956320: Nucleotide biosynthesis
R-CEL-597592: Post-translational protein modification *
GO:0007044: cell-substrate junction assembly
GO:0051640: organelle localization
GO:0051651: maintenance of location in cell
R-CEL-74160: Gene expression (Transcription)
R-CEL-110373: Resolution of AP sites via the multiple-nucleotide patch replacement pathway
GO:0033554: cellular response to stress
GO:0043484: regulation of RNA splicing
0 2 4 6 8 10 12
-log10(P)

Supplementary Figure S2. Gene annotation enrichment analysis of KNL2 interaction partners identified by Y2H, AP-MS, in Arabidopsis and by IP-MS in *C. elegans* (Supports Figure 2)

(A-C) Graphs showing the major functional pathways and biological processes with which KNL2 interactors detected by Y2H screening (A), AP-MS approach in Arabidopsis (B), and IP-MS approach in *C. elegans* (C). The functional terms related to ubiquitin-dependent process, proteasome-mediated degradation and processes known to involve kinetochore components are marked with red asterisks. The analysis was performed using Metascape software. Y2H - Yeast-two hybrid; AP-MS - Affinity purification-mass spectrometry; IP-MS - Immunoprecipitation-mass spectrometry.

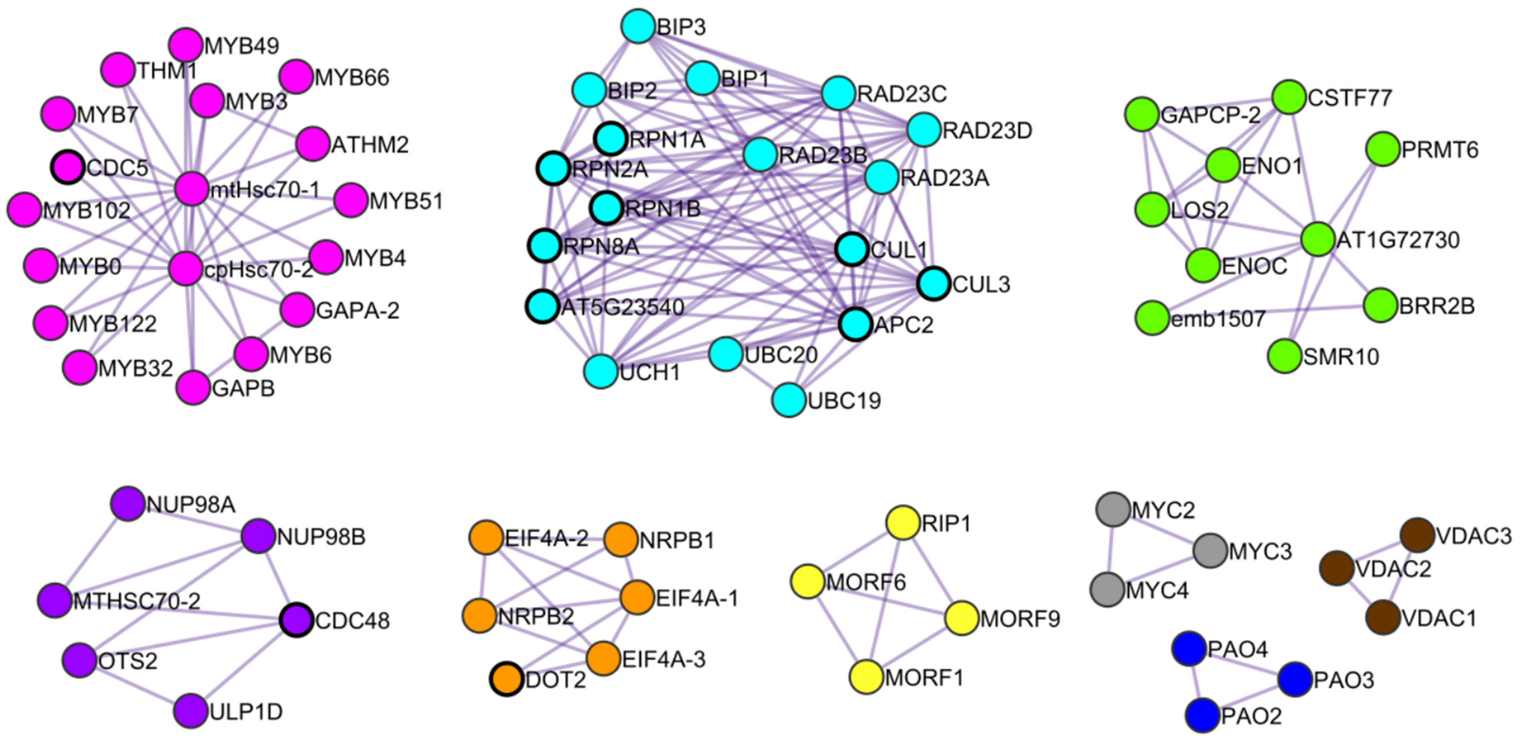

Color	MCODE	GO	Description	Log10(P)
	MCODE_1	GO:0000976, GO:0001067	Transcription cis-regulatory region binding, transcription regulatory region nucleic acid binding	-7.1
	MCODE_2	GO:0030163	Ubiquitin-dependent protein catabolic process	-19.7
	MCODE_3	GO:0008186, GO:0003724	RNA helicase activity, ATP-dependent activity	-10
	MCODE_4	GO:0031386, GO:0016925	Protein SUMOylation, Protein tag activity	-9.1
	MCODE_5	GO:0140640, GO:0006396	Catalytic activity, acting on a nucleic acid, RNA processing	-3.2
	MCODE_6	GO:0016553, GO:1900864	Base conversion or substitution editing, mitochondrial RNA modification	-11.7
	MCODE_7	GO:0071318, GO:0106167	Cellular response to ATP, extracellular ATP signaling	-10.4
	MCODE_8	GO:0046203	Spermidine catabolic process, polyamine oxidase activity	-12.2
	MCODE_9	GO:0008308	Voltage-gated monoatomic anion channel activity	-8.9

Supplementary Figure S3. The protein-protein interaction networks involving αKNL2 interactors, and their functional annotation in Arabidopsis (Supports Figure 2)

The networks including proteins detected by Y2H and AP-MS approaches were generated. All circles represent αKNL2 interaction partners revealed by the Y2H screening, circles with bold outlines indicate proteins discovered by AP-MS. Functional annotation of networks was performed using Metascape software. MCODE represents groups of proteins that form interaction networks, depicted in the corresponding colors. Y2H - Yeast-two hybrid; AP-MS - Affinity purification-mass spectrometry.

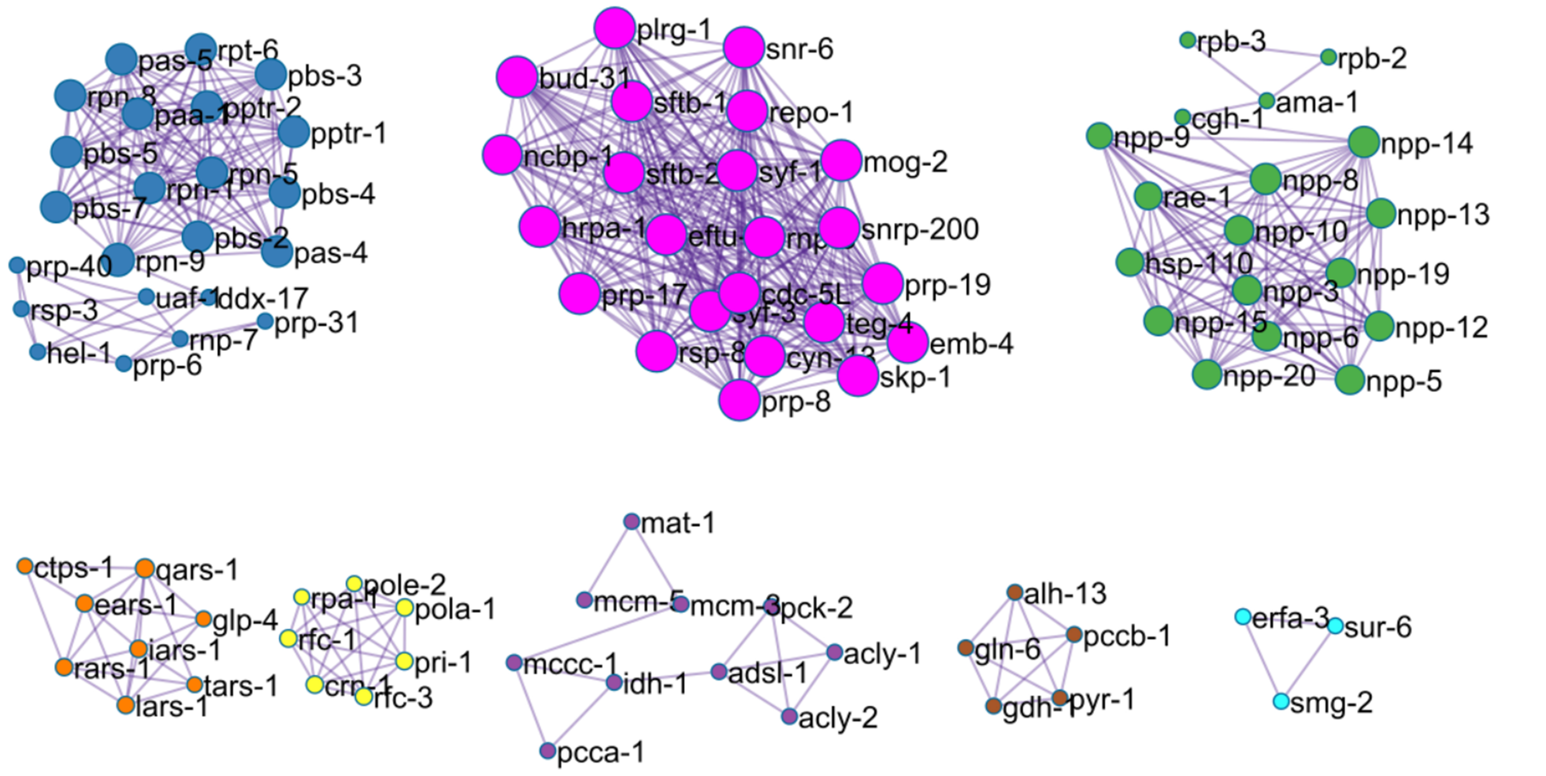

Color	MCODE	GO	Description	Log10(P)
Blue	MCODE_1	CEL03040, GO:0008380	Spliceosome, RNA splicing	-47.1
Magenta	MCODE_2	CEL-195253, CEL-1257604	Degradation of beta-catenin by the destruction complex	-31.5
Green	MCODE_3	CEL03013, GO:0017056	Nucleocytoplasmic transport, structural constituent of nuclear pore	-25.4
Orange	MCODE_4	CEL00020, GO:0072350	Citrate cycle (TCA cycle)	-8.0
Yellow	MCODE_5	GO:0006418, GO:0016875	Ligase activity, forming carbon-oxygen bonds	-16.9
Purple	MCODE_6	CEL03030, GO:0006260	DNA replication	-18.0
Brown	MCODE_7	GO:0009064, CEL-8964539	Glutamate and glutamine metabolism	-9.7
Light blue	MCODE_8	CEL-975957, CEL03015	Nonsense-Mediated Decay (NMD)	-6.6

Supplementary Figure S4. The protein-protein interaction networks involving αKNL2 interactors, and their functional annotation in *C. elegans* (Supports Figure 2)

The networks including proteins detected by IP-MS approach were generated. All nodes (circles) represent αKNL2 interaction partners revealed by the IP-MS with edges represent the interactions. Functional annotation of networks was performed using Metascape software. MCODE represents groups of proteins that form interaction networks, depicted in the corresponding colors. IP-MS - Immunoprecipitation-mass spectrometry.

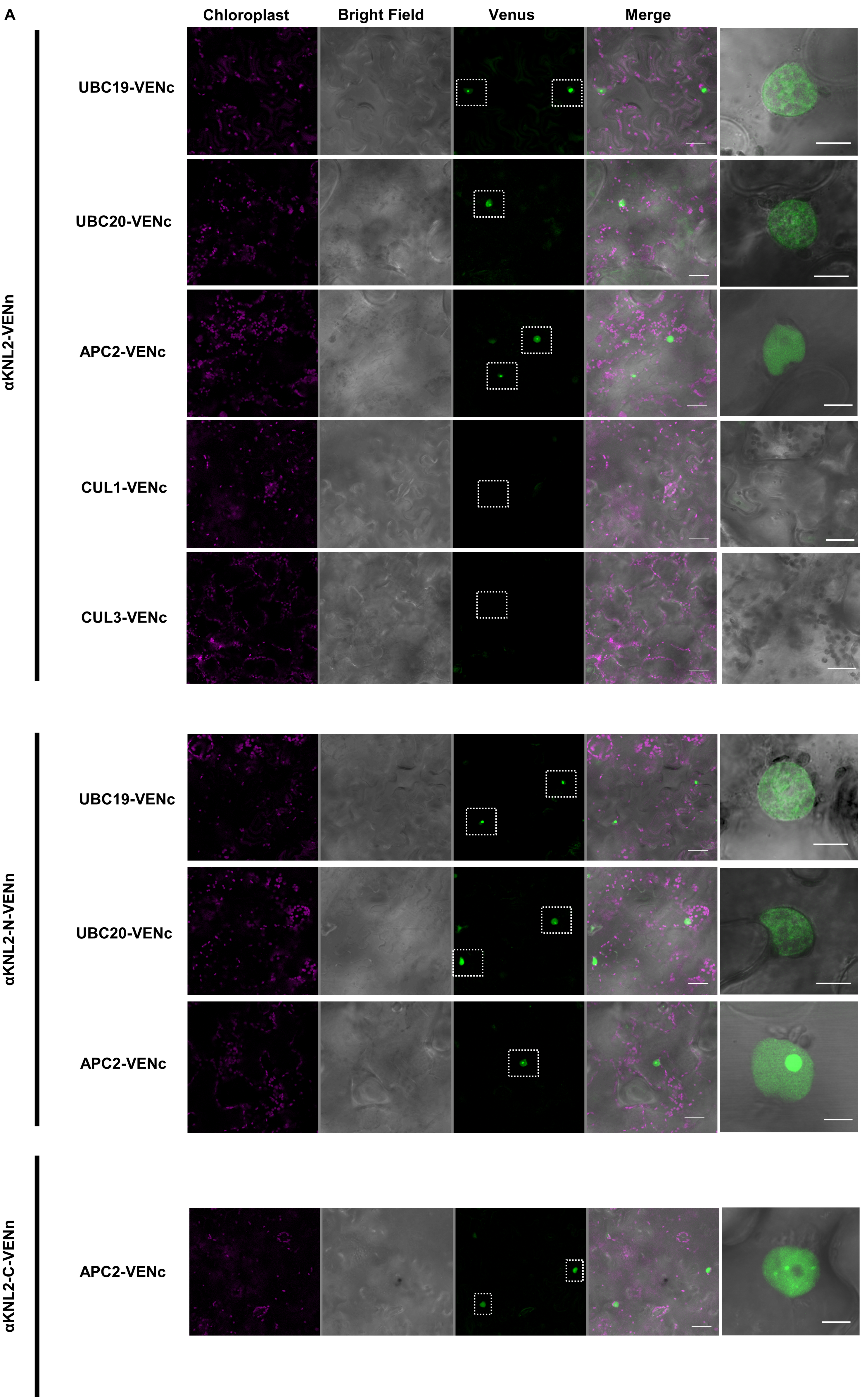
A
Chloroplast
Bright Field
Venus
Merge
αKNL2-VENn
UBC19-VENc
UBC20-VENc
APC2-VENc
CUL1-VENc
CUL3-VENc
αKNL2-N-VENn
UBC19-VENc
UBC20-VENc
APC2-VENc
αKNL2-C-VENn
APC2-VENc

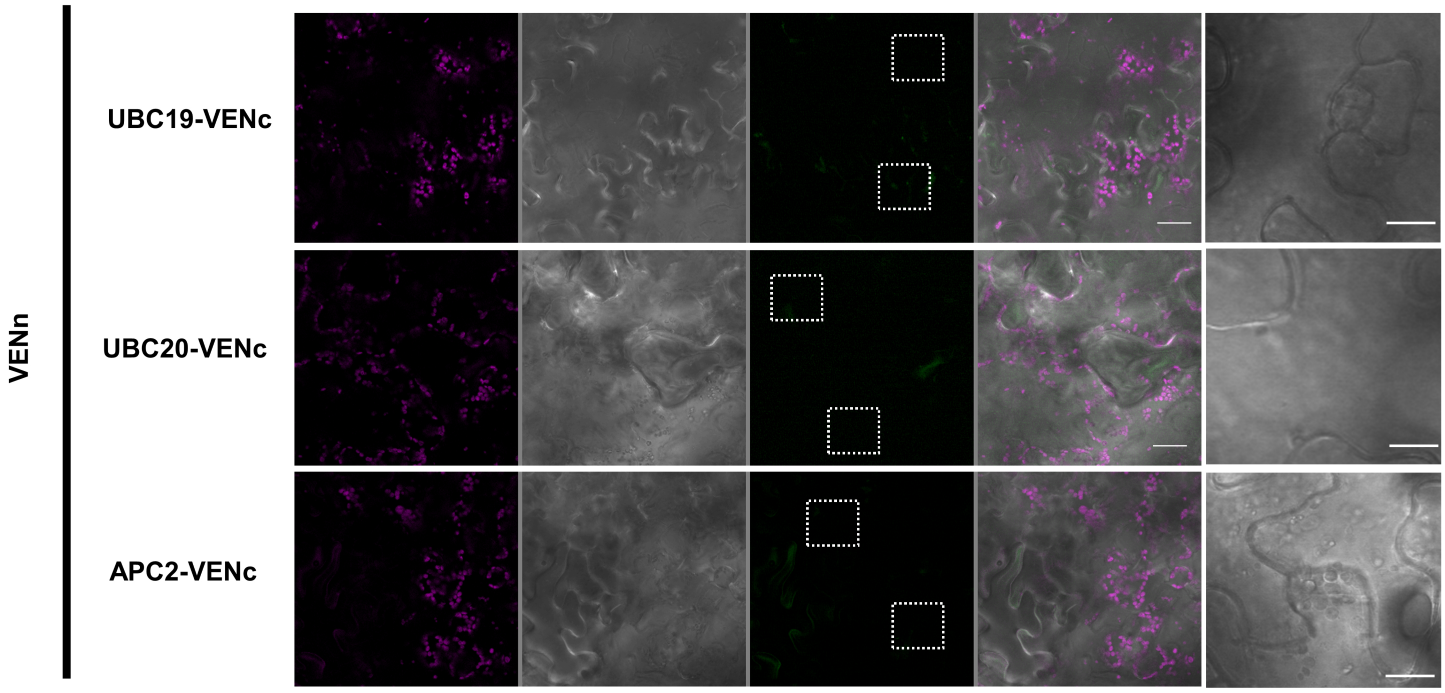

B

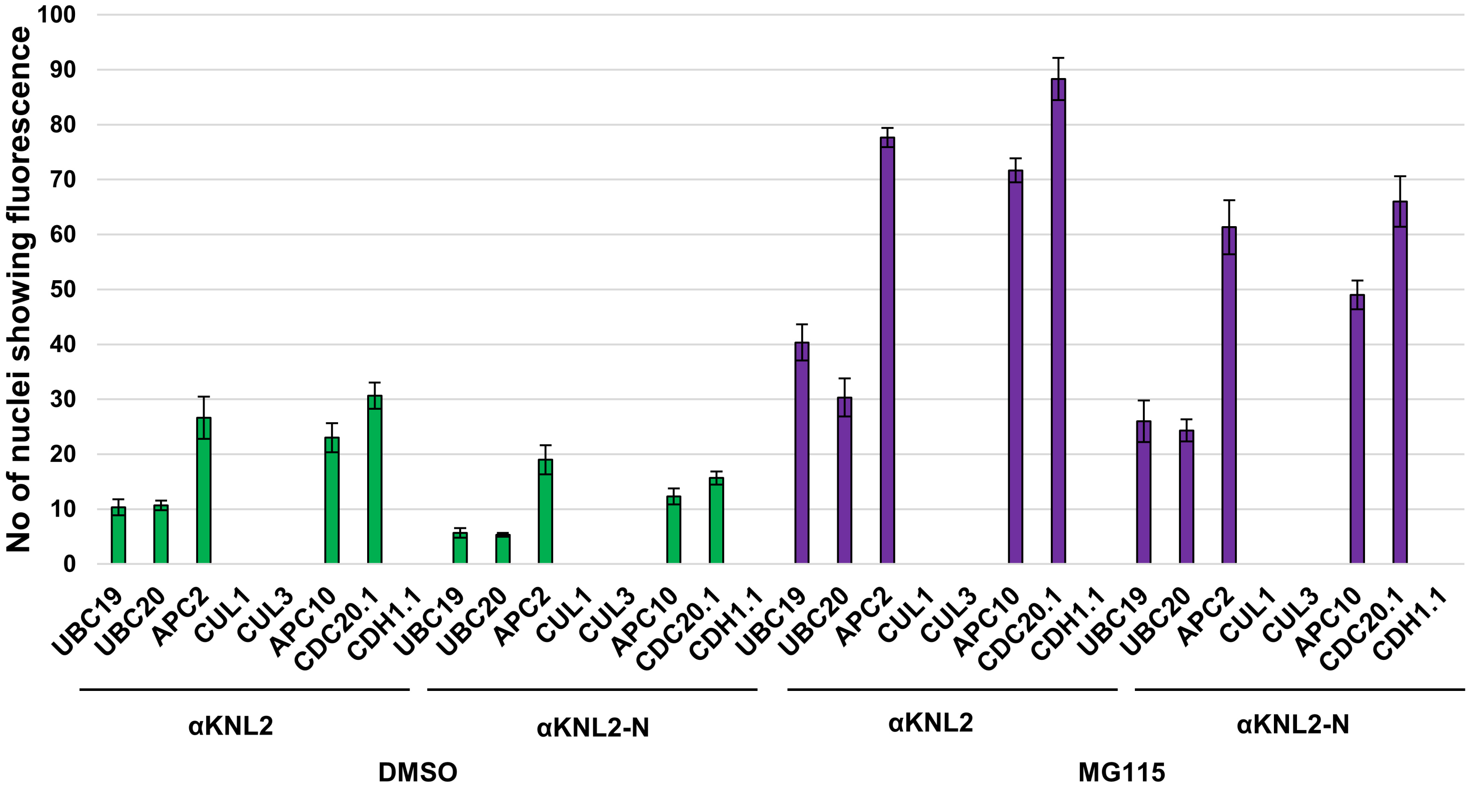

Supplementary Figure S5. BiFC interaction analysis of selected candidates with αKNL2 and quantitative analysis of BiFC assay (Supports Figure 3)

(A) BiFC analysis showing interactions between UBC19/UBC20, APC2 with αKNL2 in the nucleus, whereas CUL1/CUL3 did not show interaction with αKNL2. Venus fluorescence were shown in white dotted boxes. Scale bars represent 50 µm. The right panel shows an enlarged image of the corresponding BiFC signals in the nucleus. Scale bars represent 5 µm. **(B)** BiFC quantitative measurements of the interactions of αKNL2 and αKNL2-N are plotted based on total Venus fluorescence in DMSO and MG115 infiltrations (Area = 80 mm^2). The data are shown in mean ± SEM, n = 3.

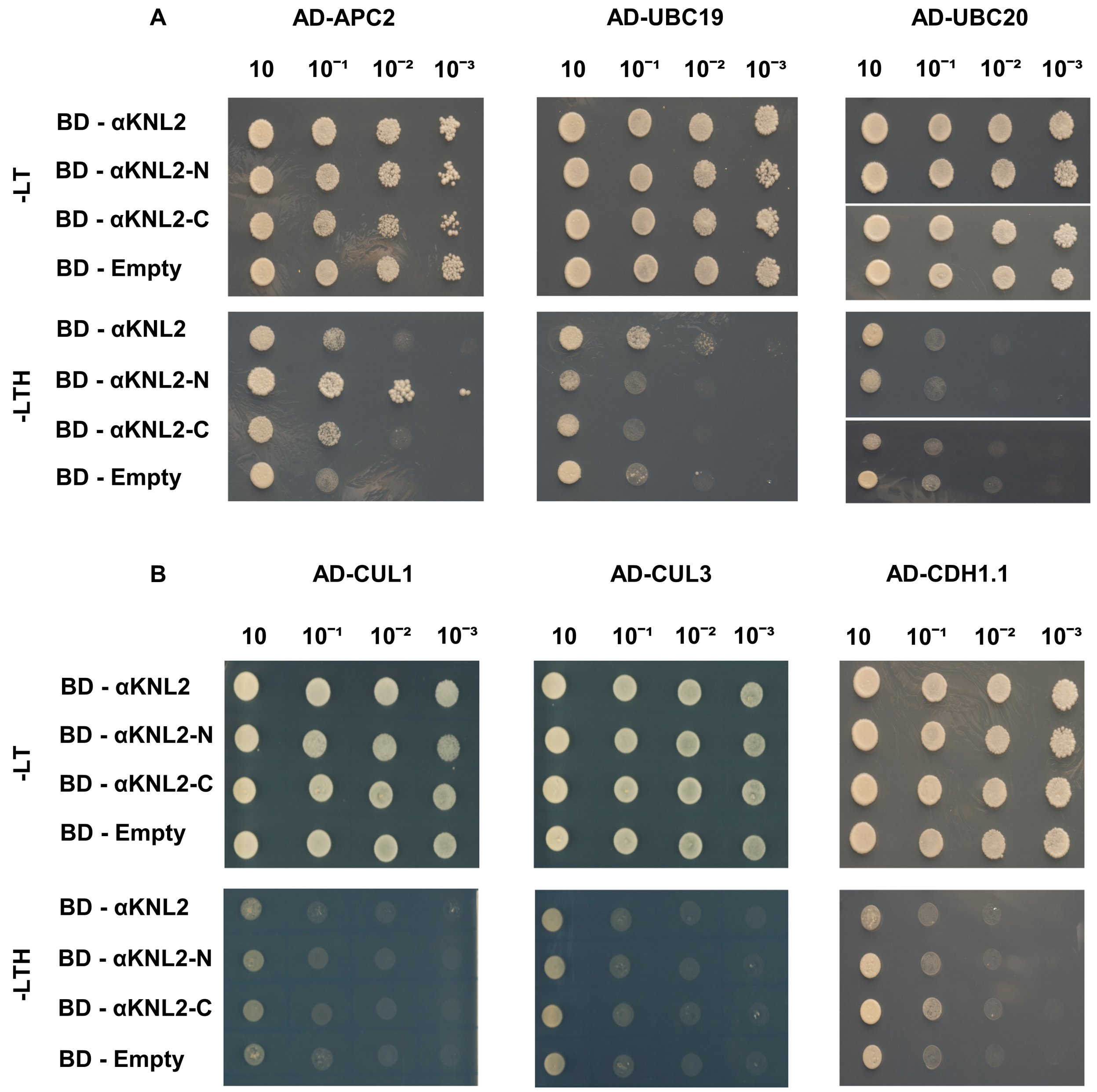

Supplementary Figure S6. Yeast two-hybrid assay showing interactions between αKNL2 (bait) and screened E2 and E3 enzymes (prey) (Supports Figure 3)

(A, B) Zygotes expressing both prey APC2, UBC19, and UBC20 (A), CUL1, CUL3, and CDH1.1 (B), and bait (αKNL2/αKNL2-N/αKNL2-C) are selected on -LT (Double dropout: YNB without Leu and Trp). Protein-protein interactions are assessed on -LTH (Triple dropout: YNB without Leu, Trp, and His). The strength of the protein-protein interactions was evaluated by drop dilution test. AD, activating domain; BD, binding domain.

***Ce*KNL-2 and *Ce*MAT-1**

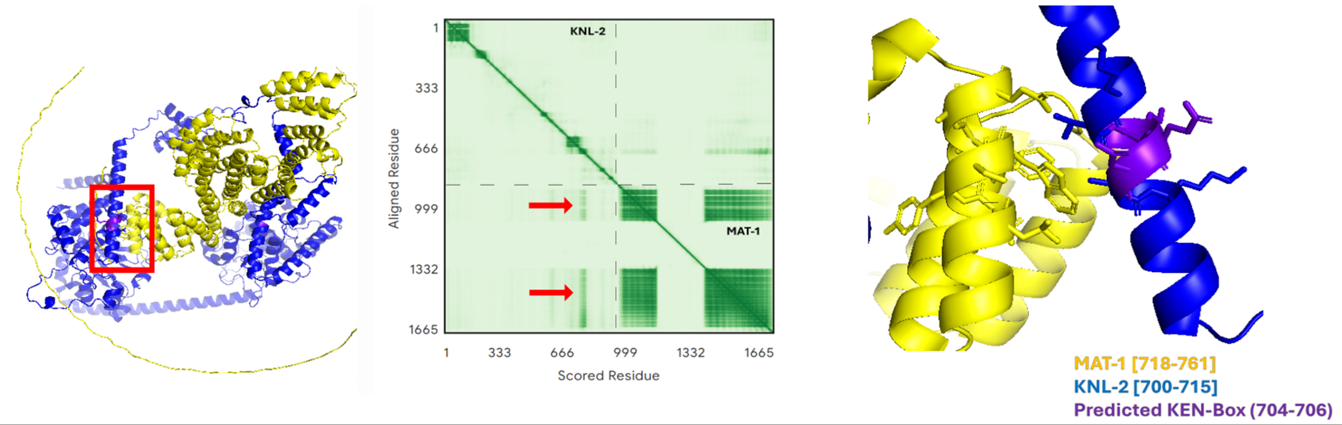

***Ce*KNL-2 and *Ce*EMB-27**

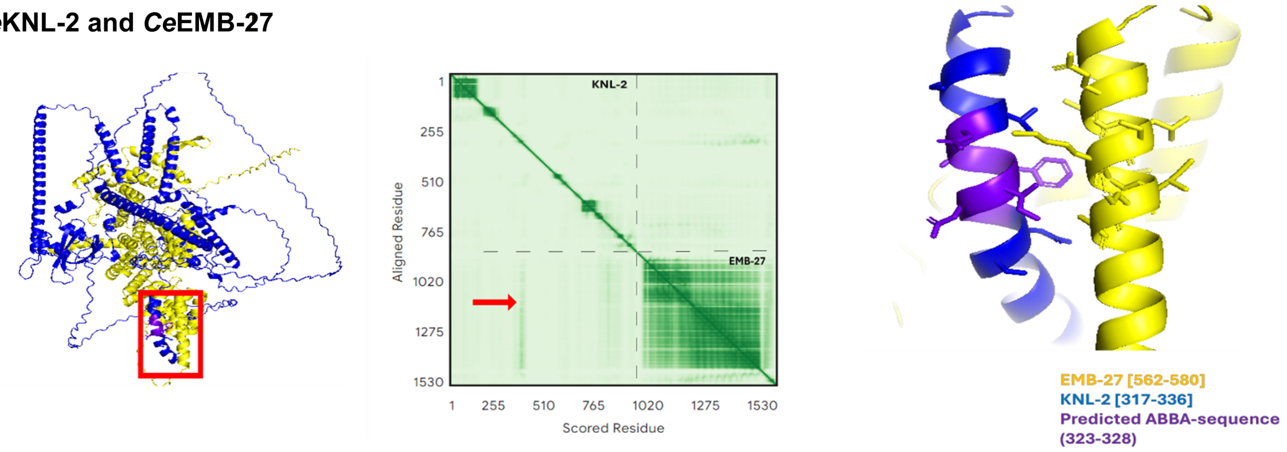

Supplementary Figure S7. AlphaFold3 interaction of *Ce*KNL-2 with MAT-1 or EMB-27 (Supports Figure 3)

AlphaFold3 predictions illustrate the interactions between *C. elegans* KNL-2 (blue) and either MAT-1 or EMB-27 (yellow). The left panels depict the predicted complexes formed by these protein pairs, with red boxes marking the interaction regions. The middle panels present heat maps of these interactions, where arrows point to the specific interaction sites. The right panels zoom in on the red-boxed areas from the left, providing a detailed view of the predicted interaction sites. Notably, MAT-1 and EMB-27 are predicted to interact with one of the APC/C-specific degron motifs (purple).

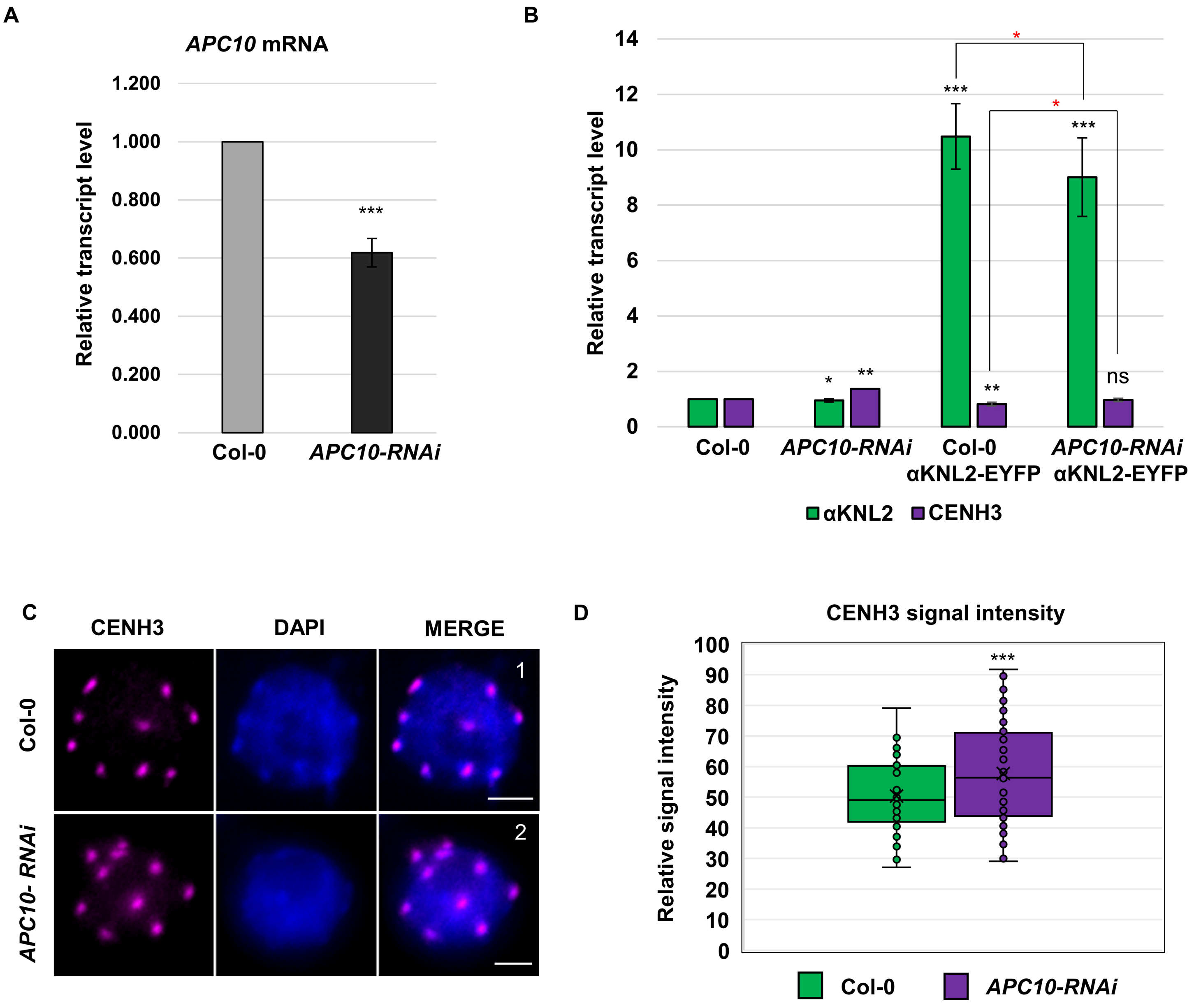

Supplementary Figure S8. Analysis of *APC10*, *αKNL2*, and *CENH3* levels in *APC10-RNAi* lines (Supports Figure 4)

(A) *APC10* mRNA levels in wild-type (Col-0) and *APC10-RNAi* plants were analyzed by RT-qPCR. Relative mRNA levels in seedlings were normalized to *ACTIN2* and *UBQ10* mRNA. Data represent the mean ± SEM of three independent experiments. Asterisks indicate the significant differences between groups, as determined by Welch's t-test (***: P < 0.005). **(B)** RT-qPCR analysis of *αKNL2* and *CENH3* mRNA levels in wild-type (Col-0), *APC10-RNAi*, and αKNL2-EYFP transformants in both the Col-0 and *APC10-RNAi* backgrounds. Relative mRNA levels in seedlings were normalized to *ACTIN2* and *UBQ10* mRNA. Data represent the mean ± SEM of three independent experiments. Asterisks indicate the significant differences between groups compared with Col-0 (*: P < 0.5; **: P < 0.05; ***: P < 0.005; ns = not significant). Red asterisks indicate significant differences comparisons between Col-0 and *APC10-RNAi* within αKNL2-EYFP groups (*: P < 0.5). Statistical analysis was performed using Welch's t-test. **(C)** Immunostaining of meristematic nuclei of Arabidopsis wild-type (1) and *APC10-RNAi* lines (2) using anti-CENH3 antibodies (magenta). Scale bars represent 5 µm. **(D)** Relative intensity measurements of CENH3 immunostaining on nuclei from *APC10-RNAi* and wild-type plants. Boxplots show the distribution of fluorescence intensities (n = 40 per group). The center line indicates the median and box limits represent the upper and lower quartiles (Q1 and Q3); whiskers extend to 1.5× the interquartile range (IQR). Statistical significance was determined using Welch's t-test (***: P < 0.005).

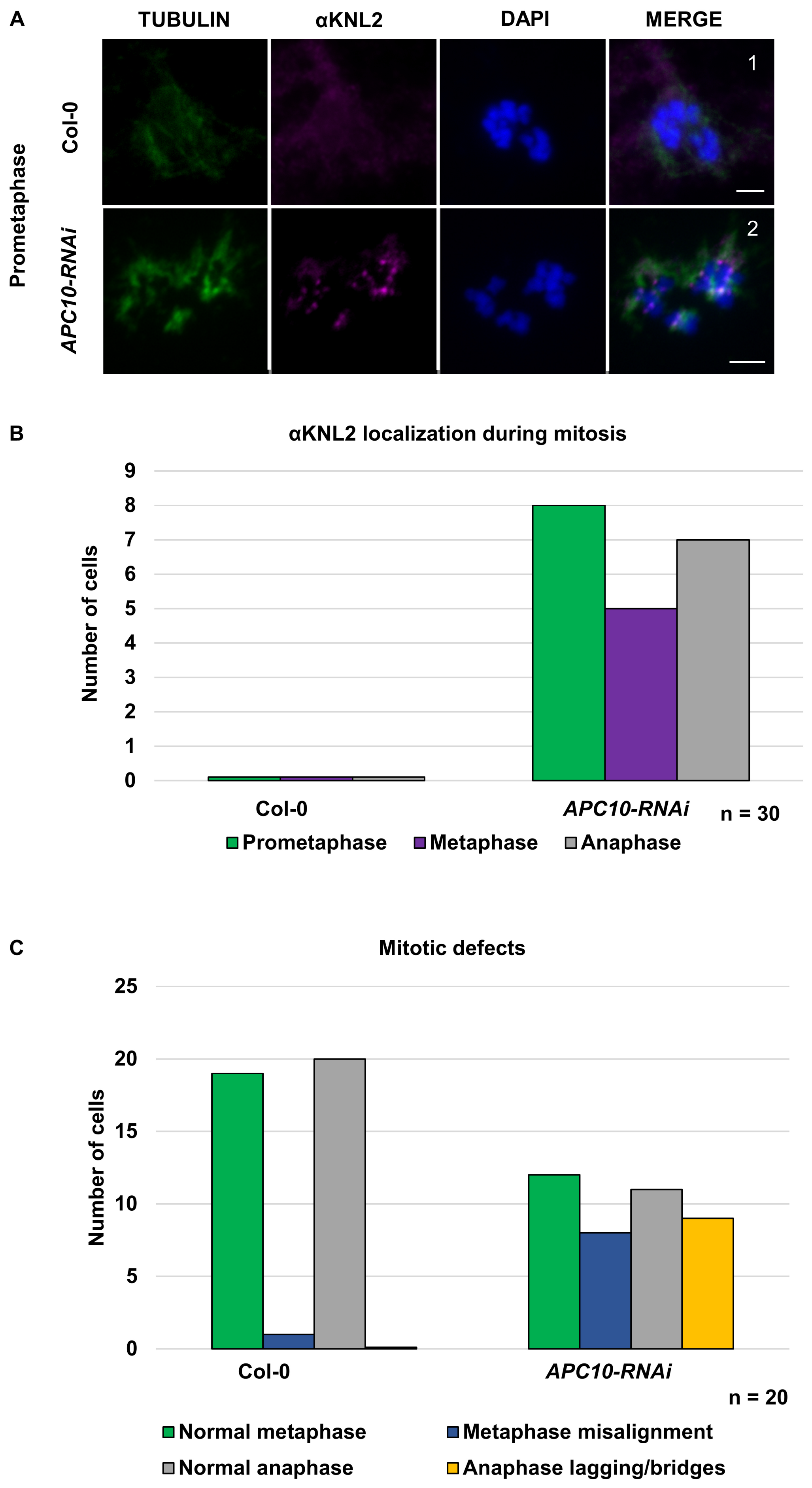

Supplementary Figure S9. The localization of αKNL2 during mitosis in cells of *APC10-RNAi* lines (Supports Figure 4)

(A) The localization of αKNL2 during prometaphase in root meristem tissues of wild-type (1) and *APC10*-RNAi (2) using anti-αKNL2 antibodies (magenta). DAPI-stained chromosomes are shown in blue, and α-tubulin are shown in green. Scale bar represent 5 µm. **(B)** The number of cells showing αKNL2 localization during prometaphase, metaphase, and anaphase was quantified in both *APC10-RNAi* and wild-type plants, with a sample size of 30 cells analyzed. **(C)** Quantification of abnormal metaphases and anaphases in *APC10-RNAi* plants comparison to wild-type. Out of 20 metaphase and anaphase cells, 40-45% of misaligned metaphases and lagging chromosomes were detected.

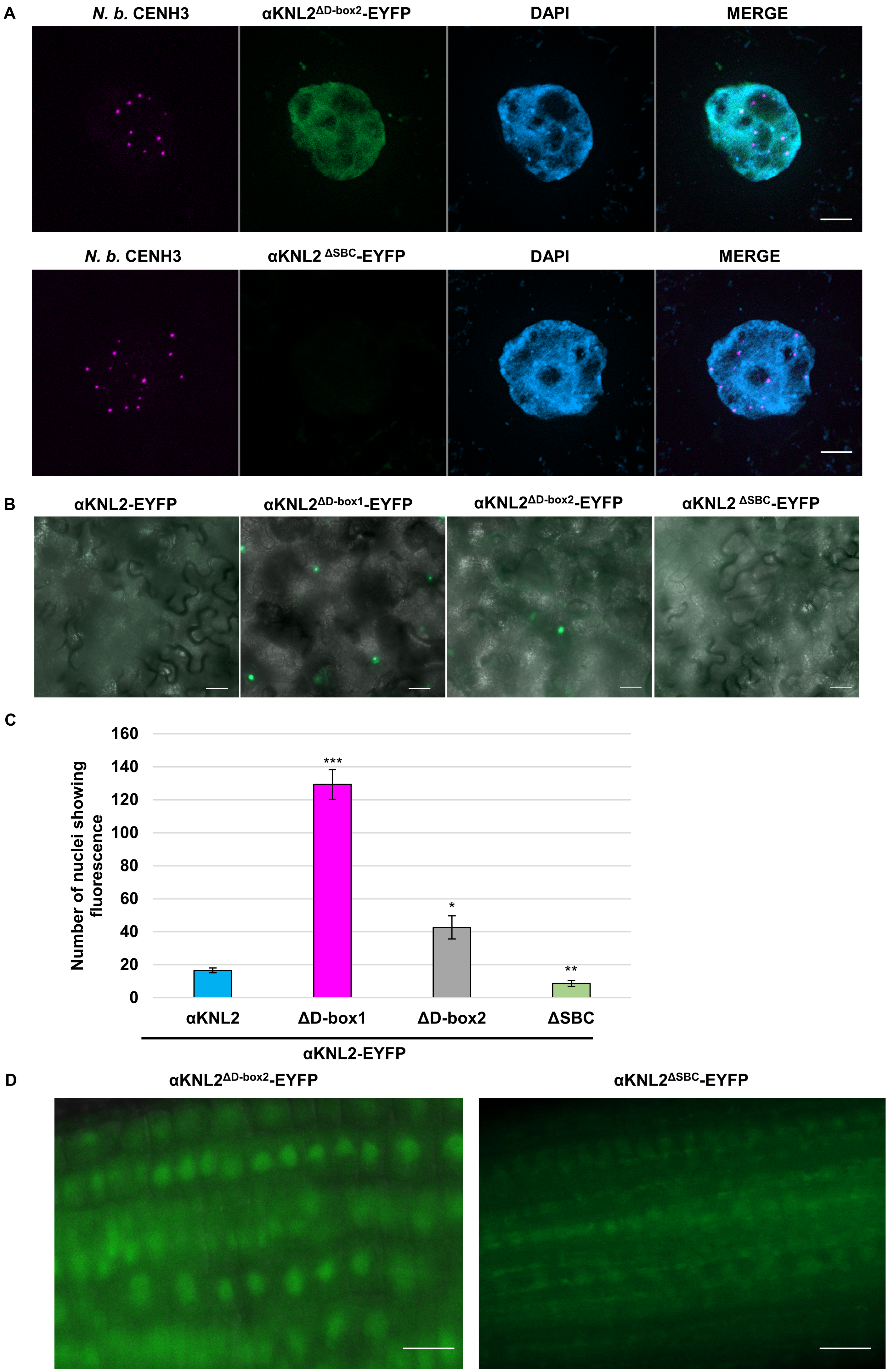

Supplementary Figure S10. D-box2 and SBC were not the active degrons in αKNL2 (Supports Figure 5)

(A) Immunolocalization of αKNL2$^{\Delta D\text{-}box2}$-EYFP and αKNL2$^{\Delta SBC}$-EYFP (green) and with *N. benthamiana* CENH3 (magenta). Scale bar represent 5 µm. **(B)** The *N. benthamiana* leaves showing the localization of αKNL2-EYFP, αKNL2$^{\Delta D\text{-}box1}$-EYFP, αKNL2$^{\Delta D\text{-}box2}$-EYFP and αKNL2$^{\Delta SBC}$-EYFP fusion constructs. Scale bar represent 20 µm. **(C)** The bar graph demonstrates the total nuclei showing fluorescence for the expression of three deletion constructs of degrons compared to αKNL2-EYFP (Area = 80 mm^2). The data are shown in mean values ± SEM, n = 3. Statistical significance between groups was evaluated using Welch's t-test and is marked by asterisks (*: $P < 0.5$; **: $P < 0.05$; ***: $P < 0.005$). **(D)** The expression of αKNL2$^{\Delta D\text{-}box2}$-EYFP and αKNL2$^{\Delta SBC}$-EYFP in Arabidopsis. Scale bar represent 10 µm.

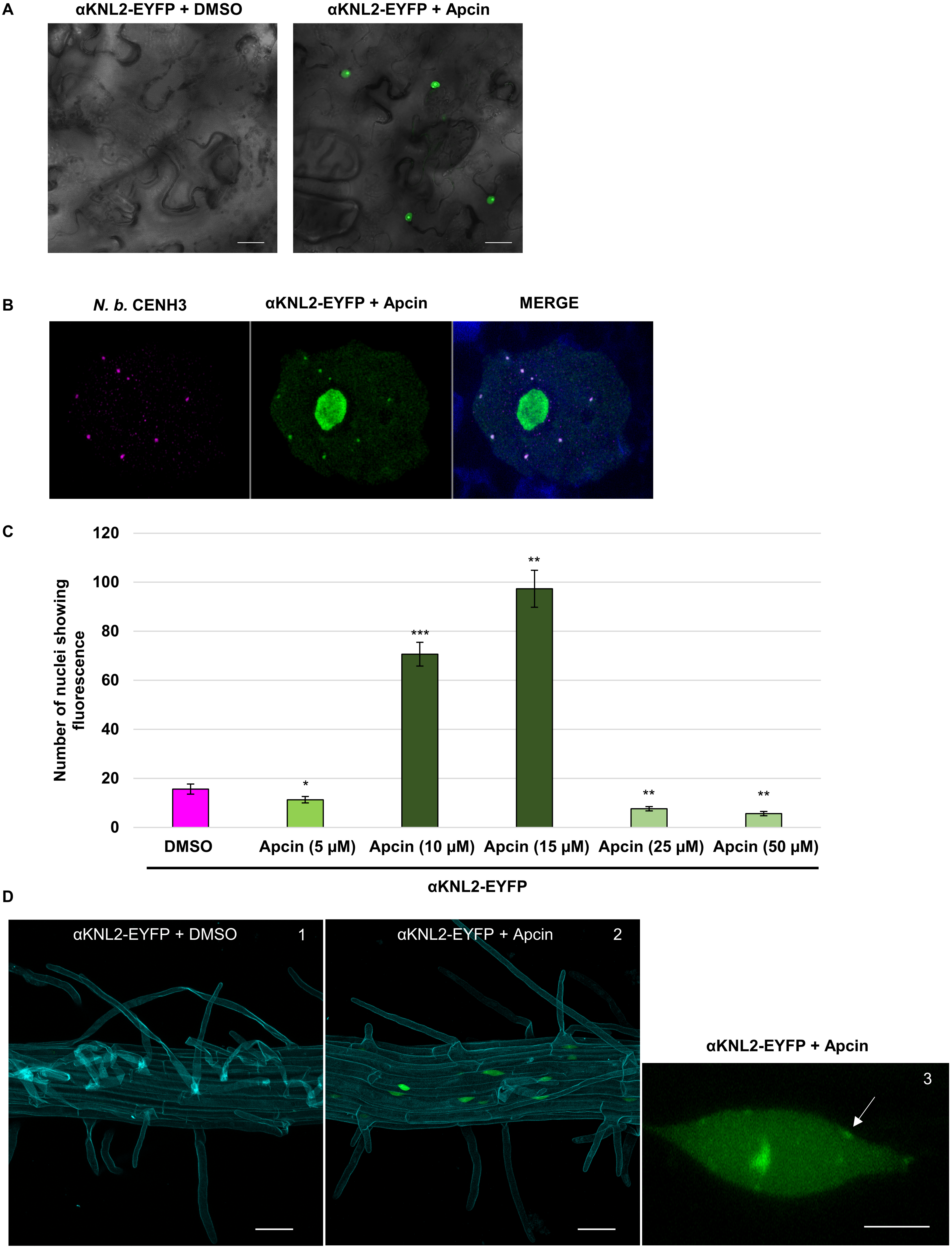

Supplementary Figure S11. Apcin inhibits the interaction of αKNL2 and APC/C complex (Supports Figure 5)

(A) Expression of αKNL2-EYFP in *N. benthamiana* treated with either DMSO (control) or Apcin, an APC/C inhibitor. Scale bar represent 50 µm. **(B)** αKNL2-EYFP treated with Apcin in *N. benthamiana* leaves showed centromere signals (green) and co-localized with CENH3 (magenta) as visualized by 3D-SIM. Scale bar represent 5 µm. **(C)** The graph representing the total nuclei showing fluorescence after treating *N. benthamiana* with different concentrations of Apcin (Area = 80 mm^2). The data are shown in mean ± SEM, n = 3. Statistical significance between groups was evaluated using Welch's t-test and are indicated by asterisks (*: $P < 0.5$; **: $P < 0.05$). **(D)** Expression of αKNL2-EYFP in Arabidopsis root tips treated with DMSO (1) or Apcin (2). Post-Apcin, αKNL2-EYFP localizes in the nucleoplasm and chromocenters shown in white arrows (3). Scale bars represent 50 µm.

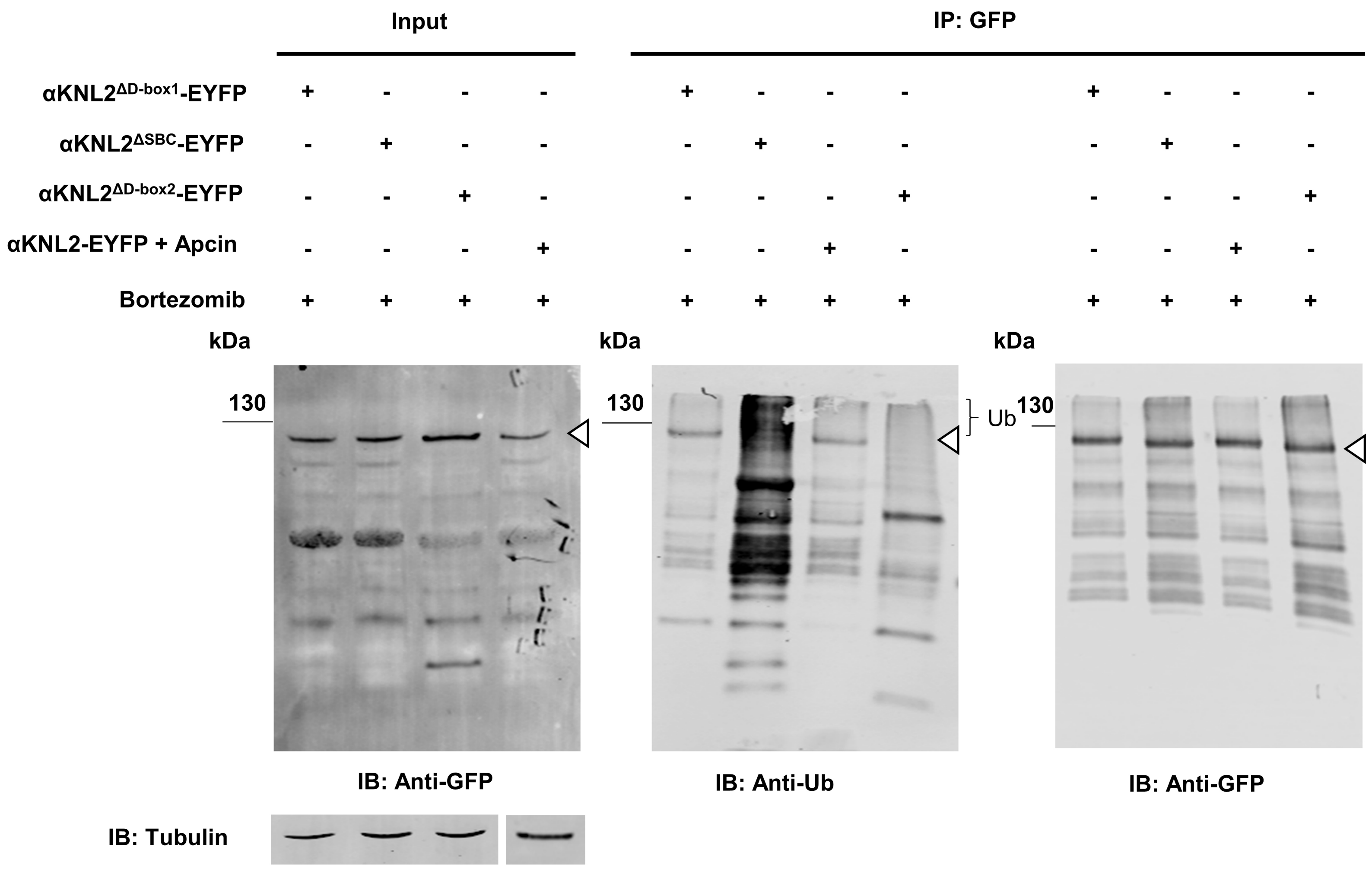

Supplementary Figure S12. The deletion of D-box1 and treatment with Apcin affects the αKNL2 interaction with ubiquitin conjugates (Supports Figure 5)

IP and IB analysis of proteins from plants expressing $\alpha KNL2^{\Delta D\text{-}box1}$-EYFP, $\alpha KNL2^{\Delta SBC}$-EYFP, $\alpha KNL2^{\Delta D\text{-}box2}$-EYFP, and αKNL2-EYFP treated with Apcin. All samples were treated with Bortezomib. Samples were immunoprecipitated with GFP beads. Input samples were blotted with anti-GFP (left), and IP samples were probed with anti-ubiquitin (Ub) (center) or anti-GFP (right) antibodies. Tubulin was used as a loading control. Triangle (▵) denotes unmodified αKNL2, while the bracket indicates its ubiquitinated form. Abbreviations: IB, Immunoblot; IP, Immunoprecipitation.

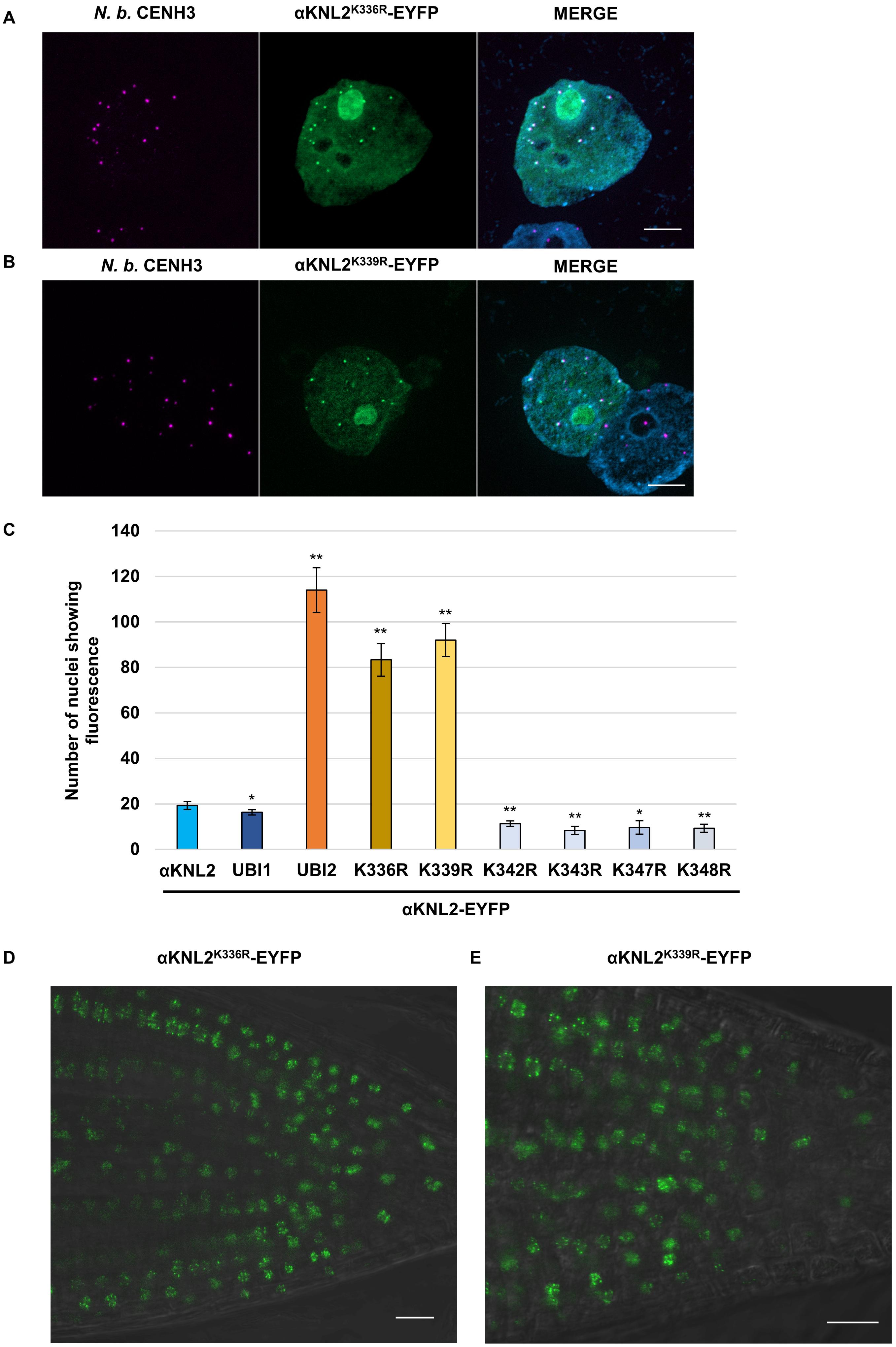

Supplementary Figure S13. 336K and 339K of αKNL2 are the targets for binding ubiquitin protein (Supports Figure 6)

(A, B) The localization of αKNL2^{K336R}-EYFP and αKNL2^{K339R}-EYFP demonstrates centromere-specific signals of αKNL2 (green) and co-localized with CENH3 (magenta) in *N. benthamiana.* Scale bars represent 5 µm. **(C)** The graph plot shows the total number of nuclei showing fluorescence for the expression of ubiquitin mutagenesis constructs compared to αKNL2-EYFP (Area = 80 mm^2). The data are shown in mean ± SEM, n = 3. Statistical significance between groups was evaluated using Welch's t-test and are shown by asterisks (*: P < 0.5; **: P < 0.05). **(D, E)** Arabidopsis root tips showing the expression of αKNL2^{K336R}-EYFP and αKNL2^{K339R}-EYFP at centromeres, similar to *N. benthamiana*. Scale bars represent 10 µm.

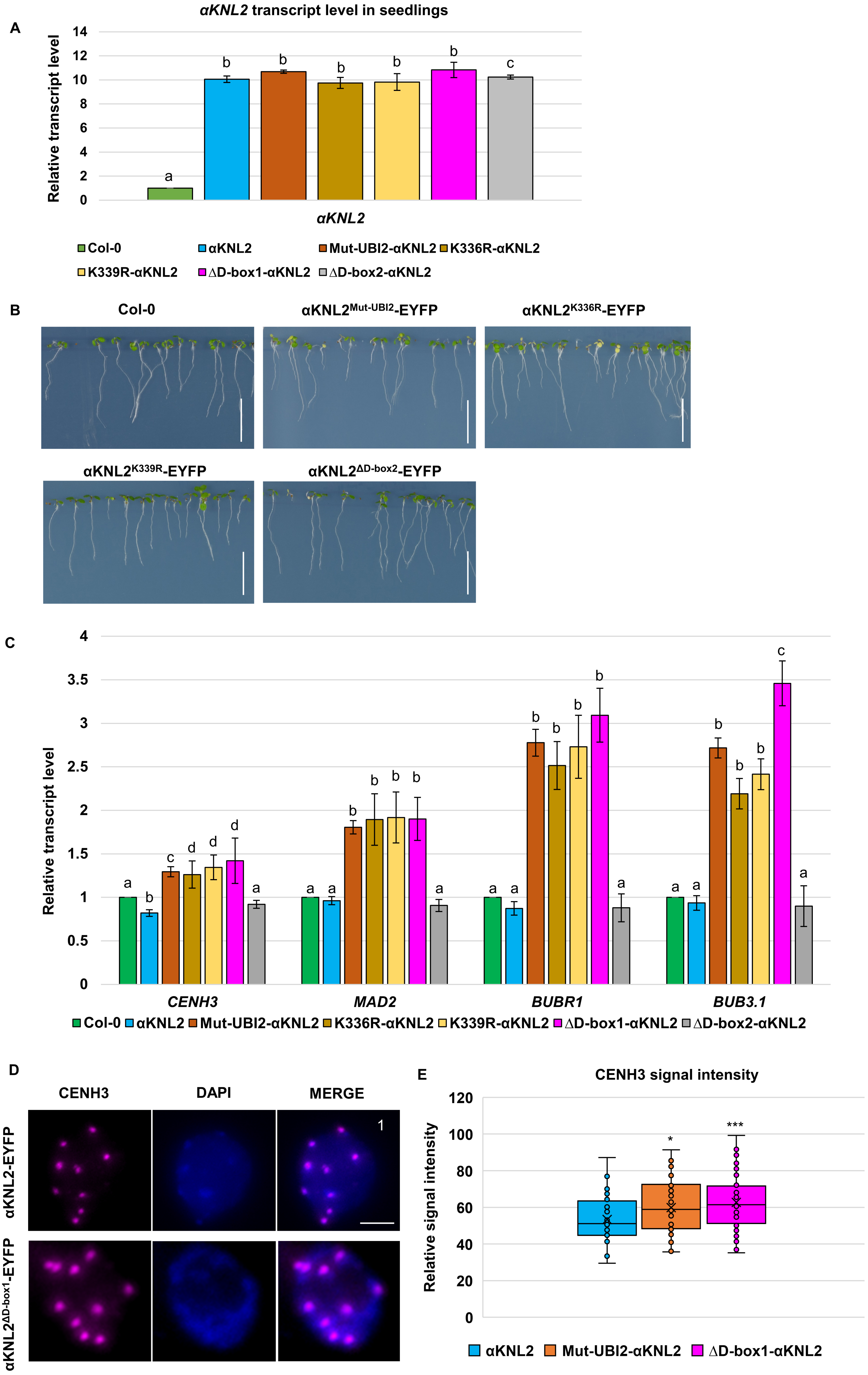
A
αKNL2 transcript level in seedlings
Relative transcript level
14
12
10
8
6
4
2
0
a
b
b
b
b
b
c
αKNL2
Col-0
αKNL2
Mut-UBI2-αKNL2
K336R-αKNL2
K339R-αKNL2
ΔD-box1-αKNL2
ΔD-box2-αKNL2
B
Col-0
αKNL2Mut-UBI2-EYFP
αKNL2K336R-EYFP
αKNL2K339R-EYFP
αKNL2ΔD-box2-EYFP
C
Relative transcript level
4
3.5
3
2.5
2
1.5
1
0.5
0
CENH3
MAD2
BUBR1
BUB3.1
Col-0
αKNL2
Mut-UBI2-αKNL2
K336R-αKNL2
K339R-αKNL2
ΔD-box1-αKNL2
ΔD-box2-αKNL2
D
CENH3
DAPI
MERGE
αKNL2-EYFP
αKNL2ΔD-box1-EYFP
1
E
CENH3 signal intensity
Relative signal intensity
120
100
80
60
40
20
0
*
***
αKNL2
Mut-UBI2-αKNL2
ΔD-box1-αKNL2

F

G

H

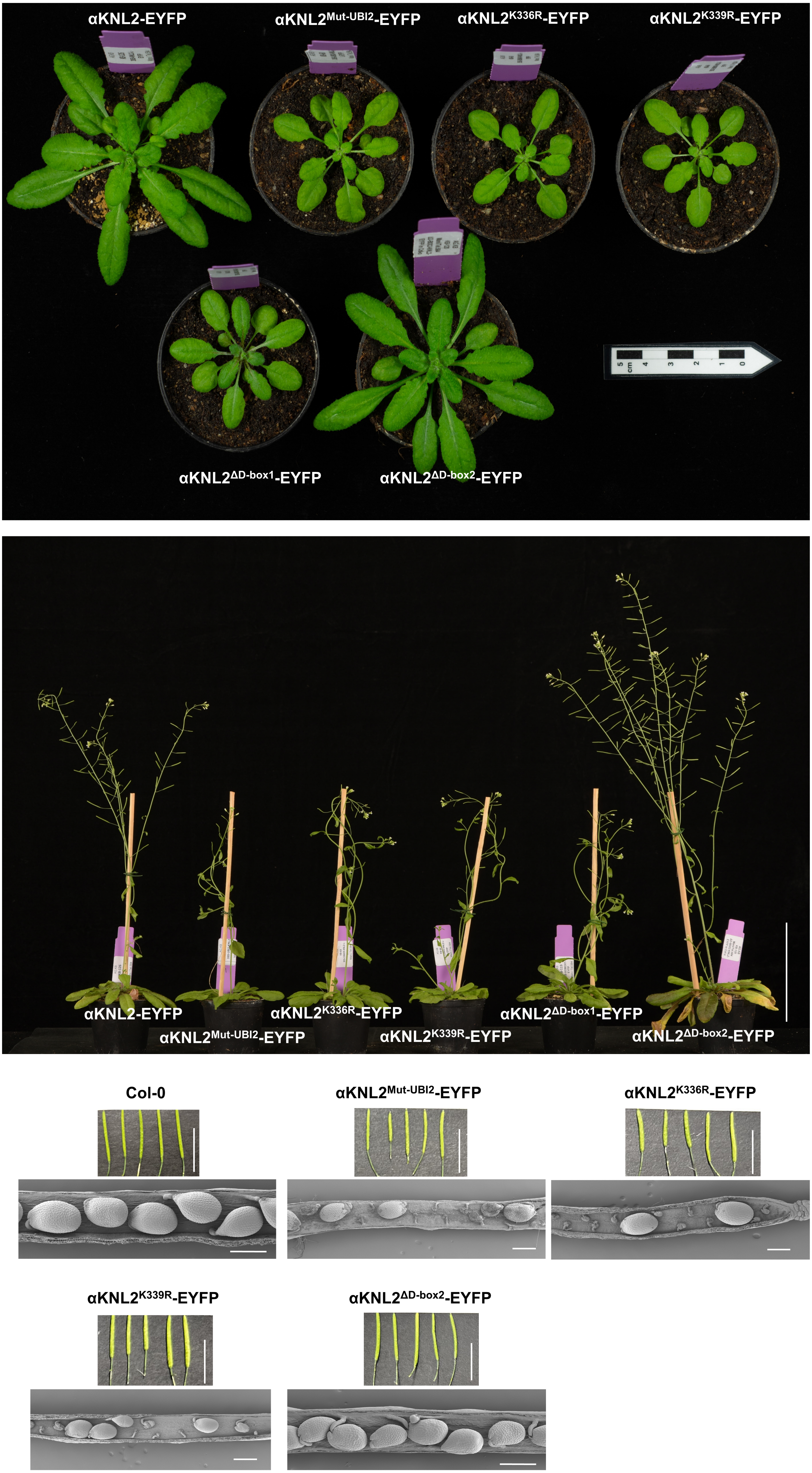

αKNL2-EYFP
αKNL2$^{Mut-UBI2}$-EYFP
αKNL2^{K336R}-EYFP
αKNL2^{K339R}-EYFP
αKNL2$^{ΔD-box1}$-EYFP
αKNL2$^{ΔD-box2}$-EYFP
cm
5 4 3 2 1 0
αKNL2-EYFP
αKNL2$^{Mut-UBI2}$-EYFP
αKNL2^{K336R}-EYFP
αKNL2^{K339R}-EYFP
αKNL2$^{ΔD-box1}$-EYFP
αKNL2$^{ΔD-box2}$-EYFP
Col-0
αKNL2$^{Mut-UBI2}$-EYFP
αKNL2^{K336R}-EYFP
αKNL2^{K339R}-EYFP
αKNL2$^{ΔD-box2}$-EYFP

Supplementary Figure S14. The phenotype characteristics of degradation-resistant plants of αKNL2 in Arabidopsis (Supports Figure 7)

(A) *αKNL2* mRNA levels in wild-type (Col-0), αKNL2-EYFP, αKNL2$^{Mut\text{-}UBI2}$-EYFP, αKNL2^{K336R}-EYFP, αKNL2^{K339R}-EYFP, αKNL2$^{\Delta D\text{-}box1}$-EYFP, and αKNL2$^{\Delta D\text{-}box2}$-EYFP plants were analyzed by RT-qPCR. Relative mRNA levels in seedlings were normalized to *ACTIN2* and *UBQ10* mRNA. Data represent the mean ± SEM of three independent experiments. Significant differences are marked by lowercase letters based on ANOVA and Tukey's multiple comparison tests ($p < 0.05$). Col-0 is used as the reference group and designated as "a". **(B)** The root growth phenotype of 7-day-old Arabidopsis seedlings with the comparison of the phenotype of degradation-resistant lines of αKNL2 mutant, and wild-type plants. Scale bars represent 1 cm. **(C)** RT-qPCR analysis of *CENH3, MAD2, BUBR1, BUB3.1* in wild-type (Col-0), αKNL2-EYFP, αKNL2$^{\Delta D\text{-}box2}$-EYFP, and degradation-resistant αKNL2 plants. Relative mRNA levels in seedlings were normalized to *ACTIN2* and *UBQ10* mRNA. Data represent the mean ± SEM of three independent experiments. Significant differences are marked by lowercase letters based on ANOVA and Tukey's multiple comparison tests ($p < 0.05$). Col-0 is used as the reference group and designated as "a". **(D)** Immunostaining of meristematic nuclei of Arabidopsis αKNL2-EYFP (1), and αKNL2$^{\Delta D\text{-}box1}$-EYFP (2) using anti-CENH3 antibodies. Scale bars represent 5 µm. **(E)** Relative intensity measurements of CENH3 immunostaining on nuclei from αKNL2-EYFP, αKNL2$^{Mut\text{-}UBI2}$-EYFP, and αKNL2$^{\Delta D\text{-}box1}$-EYFP. Boxplots show the distribution of fluorescence intensities ($n = 40$ per group). The center line indicates the median and box limits represent the upper and lower quartiles (Q1 and Q3); whiskers extend to 1.5× the interquartile range (IQR). Statistical significance between groups was evaluated using Welch's t-test and are indicated by asterisks (*: $P < 0.5$; ***: $P < 0.005$). **(F, G)** Phenotype comparison of αKNL2$^{Mut\text{-}UBI2}$-EYFP, αKNL2^{K336R}-EYFP, αKNL2^{K339R}-EYFP, αKNL2$^{\Delta D\text{-}box1}$-EYFP, αKNL2$^{\Delta D\text{-}box2}$-EYFP, with that of αKNL2-EYFP. Plants were grown for 4 weeks **(F)** or 8 weeks **(G)** on soil. Scale bars represent 10 cm. **(H)** Silique size of degradation-resistant lines of αKNL2 compared to wild-type (upper panel). Scale bars represent 1 cm, and scanning electron microscopy images of respective siliques (lower panel). Scale bars represent 20 µm.

Supplementary Table S1. BiFC interaction analysis of selected candidates with αKNL2

VENc \ VENn	αKNL2	αKNL2-N	αKNL2-C	APC2	CUL1	CUL3	APC10	CDC20.1	CDH1.1
αKNL2				Interaction	No Interaction	No Interaction	Interaction	Interaction	No Interaction
αKNL2-N				Interaction	No Interaction	No Interaction	Interaction	Interaction	No Interaction
αKNL2-C				Interaction	No Interaction	No Interaction	Interaction	Interaction	No Interaction
UBC19	Interaction	Interaction	No Interaction						
UBC20	Interaction	Interaction	No Interaction						
APC2	Interaction	Interaction	Interaction						
CUL1	No Interaction	No Interaction	No Interaction						
CUL3	No Interaction	No Interaction	No Interaction						
APC10	Interaction	Interaction	Interaction						
CDC20.1	Interaction	Interaction	Interaction						
CDH1.1	No Interaction	No Interaction	No Interaction						

Interaction (green) — No Interaction (red)

Supplementary Table S2. Y2H interaction analysis of selected candidates with αKNL2

BD \ AD	αKNL2	αKNL2-N	αKNL2-C	UBC19	UBC20	APC2	CUL1	CUL3	APC10	CDC20.1	CDH1.1
αKNL2				No Interaction	No Interaction	No Interaction	No Interaction	No Interaction	No Interaction	Interaction	No Interaction
αKNL2-N				No Interaction	No Interaction	Interaction	No Interaction	No Interaction	No Interaction	Interaction	No Interaction
αKNL2-C				No Interaction	No Interaction	No Interaction	No Interaction	No Interaction	Interaction	Interaction	No Interaction
UBC19	Auto-activation	Auto-activation	Auto-activation								
UBC20	No Interaction	No Interaction	No Interaction								
APC2	Auto-activation	Auto-activation	Auto-activation								
CUL1	No Interaction	No Interaction	No Interaction								
CUL3	No Interaction	No Interaction	No Interaction								
APC10	Auto-activation	Auto-activation	Auto-activation								
CDC20.1	Interaction	Interaction	Interaction								
CDH1.1	No Interaction	No Interaction	No Interaction								

Interaction (green) — No Interaction (red) — Auto-activation (yellow)

Supplementary Table S3. APC/C-specific degrons present in KNL2 protein across species

Species	KNL2 / MIS18BP1		
	D-box	KEN	ABBA
Homo sapiens	8	1	-
Mus musculus	4	-	-
Danio rerio	4	-	1
Caenorhabditis elegans	4	1	2
Triticum aestivum	2	-	-
Arabidopsis thaliana	2	-	-
Raphanus sativus	2	-	-
Brassica napus	3	-	-
Brassica rapa	2	-	-
Glycine max	2	-	-
Solanum tuberosum	1	-	-
Solanum lycopersicum	1	-	-

Supplementary Table S4. Primers used in this study

S. no	Gene	Forward primer	Reverse primer
	**Amplification of genes using attB primers**		
1	UBC19 (AT3G20060)	ATGGCGACGGTTAATGGGTAC	TCATGCGTTTAAAGGCTTGTA
2	UBC20 (AT1G50490)	ATGGCCGCCGTAAATGGATA	TCATGCACTTGGAGGCTTGTA
3	APC2 (AT2G04660)	ATGGAAGCTTTAGGTTCCTCTG	CTTCTTTAGCAAATACATACCATC
4	CUL1 (AT4G02570)	ATGGAGCGCAAGACTATTGAC	AGCCAAGTACCTAAACATG
5	CUL3 (AT1G26830)	ATGAGTAATCAGAAGAAGAGG	GGCTAGATAGCGGTAAAGTTTC
6	APC10 (AT2G18290)	ATGGCGACAGAGTCATCGG	TCTCAGTGTTGAATAAGTGAG
7	CDH1.1 (AT4G22910)	ATGGCATCGCCACAGAGTAC	TCGGATCTGTGTCCTCCCC
8	CDC20.1 (AT4G33270)	ATGGATGCAGGTATGAACAAC	ACGAATACGATTCACGTGAG
	**Primers used to confirm the positive entry and destination clones**		
9	attB1	GGGGACAAGTTTGTACAAAAAAGCAGGCTTC	
10	attB2	GGGGACCACTTTGTACAAGAAAGCTGGGTC	
	**Primers used for PCR-based site-directed mutagenesis**		
11	ΔSPOP-αKNL2	CCAATCACCAAAGCTTTGGATGTTT	ACGCATTGCTCTTGTCTCGACGGAC
12	ΔD-box1-αKNL2	AATGGATTTAACCCTGAGATTTCTC	AGATGCTAAGAGTGTGAAAACATCC
13	ΔD-box2-αKNL2	CTTGAATCGAGTAAAGTCCGGAAGA	TGCTCCAGAACCACCTTTATCATTA
14	K336R-αKNL2	AGACTTAGCCAGGAGTAGCAAACC	CTAAGCAGGGAATGTGGAGATGCTG
15	K339R-αKNL2	CAAGAGTAGCAGACCTGAAAAGA	GCTAAGTCTTTAAGCAGGGAATGTG
16	K342R-αKNL2	CAAACCTGAAAGGAAAGGAATAT	CTACTCTTGGCTAAGTCTTTAAGC
17	K343R-αKNL2	ACCTGAAAAGAGAGGAATATCCA	TTGCTACTCTTGGCTAAGTCTTTAA
18	K347R-αKNL2	AGGAATATCCAGGAAAAGTGGCA	TTCTTTTCAGGTTTGCTACTCTTGG
19	K348R-αKNL2	GAATATCCAAGAGAAGTGGCAAGA	CTTTCTTTTCAGGTTTGCTACTCTT
	**Primers used for generation of constructs for AP-MS screening**		
20	αKNL2-N	αKNL2_N_SalIf<br>TGGTCGACATGACGGAACCAAATCTCGAC	αKNL2_N_BamHIr<br>CTGGATCCGGGATCTACTACATTGTCGTC
21	αKNL2-C	αKNL2_C_SalIf<br>CGGTCGACATGAATTACTCTGGGACGAAAG	αKNL2_C_BamHIr<br>CCGGATCCTTTGATTTTCAAGTTTCTTCG
	**Primers used for RT-qPCR analysis**		
22	qαKNL2	TCGACTTTGATGTGGAGGTAACAC	GAATCAGTAGACGCCGCATTGG
23	qCENH3	GCAGGTCCAACTACGACCC	GCTGGTGAAGTTGTAGGATTTGT
24	qMAD2	GCTTGAAGCTGGGAAGTTGCAG	ATCACTCTTCTCCCTCGACACACC
25	qBUBR1	TCTCTAGGGATGCAAAGCCAGTG	GTTGGACCTTCTCATCGTTCTCAC
26	qBUB3.1	TCCTCGTTTCTTCATGGGATAAGC	TCTCCTTTCAACGAATTGGTGCTC
27	qAPC10	TGTCAAATCACCTCAACGGGAGAG	AGGTTGGTGCGGAATAGGATTTGG
